# JON 63rd Meeting 2024 Park City ABSTRACTS

**DOI:** 10.2478/jofnem-2024-0036

**Published:** 2024-09-11

## A molecular evolutionary cascade facilitates nematode parasitism of prey carrying toxic cardiac glycosides

Achi, Perla^1^, A. Baniya^1^, C. Goldy^1^, V. Iglesias^1^, P. Christensen^1^, A. Aljidui^1^, D. Godinez-Vidal^1^, R. S. Fonseca^2^, K. Anesko^1^, R. Adrianza^1^, P. M. Douglas^2^, A. R. Dillman^1,3^ and S. C. Groen^1,3^

^1^Department of Nematology, University of California, Riverside, CA

^2^Dept. Molecular Biology and Hamon Center for Regenerative Science and Medicine, University of Texas Southwestern Medical Center, Dallas, TX

^3^Center for Infectious Disease and Vector Research, Institute for Integrative Genome Biology, University of California, Riverside, CA

### Abstract

Target-site insensitivity (TSI) is an important mechanism of animal resistance to natural and man-made toxins. TSI evolved in parallel in the monarch butterfly and other insects specializing in milkweeds and is thought to have facilitated the sequestration of cardiac glycosides (CGs) that may protect these insects from predation and parasitism. Substitution N122H in the CG-binding pocket of the molecular target, the Na^+^/K^+^-ATPase alpha subunit (ATPα), strongly enhanced TSI and evolved in parallel in CG-sequestering insects across six orders. Upon performing a genetic screening of the Na^+^/K^+^-ATPase, we recently identified N122H in the entomopathogenic nematode (EPN), *Steinernema carpocapsae*, which parasitizes insects around milkweeds. This sets up the possibility that the parallel evolution of N122H may not only have facilitated CG sequestration by insects but also nematode parasitism of CG-carrying insects. Here, we show that N122H is rare among nematodes and that, among species tested for CG tolerance, *S. carpocapsae* showed significantly stronger insensitivity to diverse CGs than nematodes without N122H, including free-living *Caenorhabditis elegans* and parasites of milkweed roots. CRISPR gene editing in *C. elegans* showed N122H is sufficient for overcoming toxicity of CG levels found in sequestering insects. However, N122H was accompanied by costs related to nervous system robustness, potentially explaining its rarity among nematodes. Finally, *S. carpocapsae* was the only EPN tested that was highly successful at infecting CG-carrying insects and that displayed attraction to CGs. Taken together, our results suggest that a molecular evolutionary cascade of parallel substitutions across hosts and parasites, last sharing common ancestry 600 million years ago, may shape multitrophic interactions.

## Cardiac Glycoside sequestration influences multitrophic interactions between herbivorous insects and their nematode parasites around milkweeds

Achi, Perla^1^, L. Bavier^1^, P. Christensen^1^, V. Iglesias^1^, C. McCarthy^1^, R. Pena^1^, C. Goldy^1^, A. P. Hastings^2^, A. A. Agrawal^2,3^, S. C. Groen^1,4,5^ and A. R. Dillman^1,4^

^1^Dept. Nematology, University of California Riverside, Riverside, CA

^2^Dept. Ecology and Evolutionary Biology, Cornell University, Ithaca, NY

^3^Dept. Entomology, Cornell University, Ithaca, NY

^4^Center for Infectious Disease and Vector Research, Institute for Integrative Genome Biology, University of California Riverside, Riverside, CA

^5^Dept. Botany and Plant Sciences, University of California Riverside, Riverside, CA

### Abstract

Plants employ toxins as defense mechanisms against herbivorous animals, compensating for their immobility. The genus Asclepias (milkweeds) plants synthesize toxins known as cardiac glycosides (CGs) that inhibit animal Na^+^/K^+^ATPases, often leading to impeded development, reduced fecundity, or death of herbivores. Insects that have specialized in feeding on milkweed plants employ a variety of mechanisms to tolerate and, in some cases, even sequester these toxins. CGs occur as mixtures with both polar and nonpolar characteristics, the latter typically being more toxic. Insects such as monarch butterflies and milkweed bugs appear to tolerate polar cardenolides better, preferentially sequestering these in different bodily compartments. Here, we test the hypothesis that potential parasites of such specialized herbivores are differentially affected by CGs depending on the diversity and localization patterns of CGs in toxin-sequestering insect hosts. To investigate, parasite infection experiments were conducted on insects that contained either mixtures of milkweed-derived milkweed derived CGs or purified CGs with a range of polarities: ouabain, digoxin, and digitoxin. CG-tolerant adult milkweed bugs, as well as CG-tolerant mutant Drosophila larvae were either fed or injected with these compounds, followed by infection with three insect parasites: the entomopathogenic nematodes *Steinernema carpocapsae*, *S. feltiae*, and *S. hermaphroditum*. The nematode *S. carpocapsae*, which is known to occur in soil near milkweed plants, not only exhibited a competitive advantage in infecting both CG-containing hosts but also demonstrated an attraction to mixtures of CGs in milkweed root extracts and to the purified CG ouabain.

## Assessing the impact of winter cover crops on soil nematode communities in corn-soybean rotations

Akanwari, Jerry^1,2^, P. Liang^1^ and T. Sultana^2^

^1^Brock University, Department of Biological Sciences, ON, L2S 3A1

^2^Agriculture and Agri-Food Canada, London Research and Development Center, Vineland Station, ON, L0R 2E0

### Abstract

The integration of winter cover crops (WC) in corn-soybean rotations has gained popularity among growers in Ontario. This is partly due to the recommendations from the Ontario Ministry of Agriculture, Food and Rural Affairs based on observed positive impacts on crop yield, soil health, pest management, and the environment. While WCs have demonstrated positive effects on free-living nematodes, there is also a risk of increasing the abundance and diversity of plant parasitic nematodes. These contrasting results call for a comprehensive assessment of the impact of WCs in corn-soybean rotations in Ontario and other regions that use similar practices. Thus, the objective of this study is to employ a metabarcoding approach to investigate the impact of WCs on soil nematode community composition and structure relative to conventional fallow. We conducted a study on agricultural sites that have integrated WC in soybean-corn rotation for a minimum of 5 years. The sites had four distinct categories: fallow (no WC); single WC (utilizing only rye), mixture 1 (combination of oats and rye/barley) and mixture 2 (Oats and Rye). Our study shows that a mixture of cover crops had significantly higher nematode species richness compared to conventional fallow, with these systems dominated by diverse omnivores and predators (structure index >50%). Also, the use of cover crops mixture increased the abundance of plant parasitic nematodes (>70%) relative to conventional fallow but has been shown to improve the soil food web conditions (N and C cycling) with a total improvement in the soil health conditions. The conventional fallow had a significantly higher channel index (>58%), suggesting that the decomposition of this system is mediated more by fungi feeders than bacteria feeders. Our study provides insights into the bottom-up effect of cover crops on nematode communities and that the choice of cover crops has the potential to improve soil health conditions, suppress plant parasitic nematodes, and promote free-living nematodes. Therefore, WCs can be incorporated into intensive agricultural production systems to enhance the microbe-mediated ecosystem services and promote sustainable agriculture.

## Top nematode threats to global crop production: past, present and future

Alake, Gideon^1^ and M. Nasamu^2^

^1^Dept. Entomology and Nematology, University of Florida Gainesville, FL, 32608

^2^RSK ADAS Ltd, Soils, Crops and Water, North Yorkshire, UK

### Abstract

Plant-parasitic nematodes are diverse pests that significantly impact global agricultural systems. We conducted a retrospective analysis of nematode research spanning over six decades by extracting and analyzing nematode keywords from a comprehensive corpus of journal articles. We searched the Dimension AI database for keywords such as ‘nematodes,’ *‘Meloidogyne,’* and *‘Heterodera’* and exported the results to Excel files. The dataset included metadata such as titles, abstracts, the journals where the articles were published, funding agency information, authors and affiliations, article citations, and additional information. We used custom Python codes to clean the data and remove any inconsistencies to ensure the accuracy of the results. Before the keyword extraction from the abstracts, we assembled a nematode corpus, a dictionary consisting of over 30 plant-parasitic nematode genera and representative species. We queried the nematode corpus on the abstracts and extracted and counted the frequency of terms found. Subsequently, we employed a set of custom Python and R codes to analyze and visualize the data. Our study aims to elucidate the persistent and evolving challenges posed to global agriculture by the top ten most economically important plant-parasitic nematodes. Based on mentions in scholarly articles, we identified the following top ten nematode genera in the order of importance and research commitments: *Meloidogyne*, *Pratylenchus*, *Heterodera*, *Globodera*, *Bursaphelenchus*, *Xiphinema*, *Helicotylenchus*, *Radopholus*, *Ditylenchus*, and *Aphelenchoides*. The most studied nematode genera, *Meloidogyne*, accounted for over 100,000 mentions in scholarly articles, with *M. incognita* alone appearing in almost half of the studies. This genus exemplifies nematologists’ intensive research efforts to understand and document its impact on agriculture, particularly through plant root infection that significantly reduces crop yields worldwide. The genera *Pratylenchus*, *Heterodera*, and *Globodera* closely followed the trend. The analysis of the regional patterns in nematology research identified the United States, Brazil, China, India, and the United Kingdom as key nematode research hubs. This is consistent with their status as major agricultural producers. These countries still face significant nematode-related challenges even with robust agricultural research frameworks. This comprehensive analysis sheds light on the evolving landscape of nematode research, highlights the crucial roles nematodes play in global agriculture, and emphasizes the positive impact of nematologists in enhancing crop health and improving food security worldwide through consistent research innovations. Additionally, this study provides insights into future research directions that present exciting opportunities for continued progress in nematology.

## Quantification of damage and yield losses and management of *Meloidogyne exigua* in arabica coffee treated with biofertilizers, biopesticides and chemical nematicide

Alves, Fábio Ramos^1^, W. B. Moraes^1^, D. H. S. G. Barbosa^2^, A. S. Xavier^1^, J. M. Pinto^1^, S. C. Gomes^1^ and I. P. Dutra^1^

^1^Federal University of Espírito Santo, Center for Agricultural Sciences and Engineering, Alegre, ES, 29500-000, Brazil

^2^Embrapa Cassava and Tropical Fruit Culture, Cruz das Almas, BA, 44380-000, Brazil

### Abstract

The objective of this work was to verify if two biofertilizers, two biopesticides and a chemical nematicide were able to reduce the population of *M. exigua* in arabica coffee and, consequently, the damage and yield losses caused by the pathogen to the plants and increase the productivity of the coffee bush. The following treatments were used: T1: commercial organic biofertilizer containing *Bacillus subtilis*, *B. licheniformis* and *Lactobacillus* sp. (Bio_1_); T2: Bio_1_ + Bio_1_; T3: Bio_1_ + 100% organic commercial fluid biofertilizer rich in organic matter, humic and fulvic acids (Bio_2_); T3: Bio_1_ + Bio_2_; T4: commercial biopesticide based on *B. subtilis* and *B. licheniformis* (Bio_3_); T5: commercial biopesticide based on *Trichoderma harzianum* (Bio_4_); T6: Fluensulfone (Flu); T7: Bio_1_ + Flu and T8: Control. The experiment was carried out in a field of arabica coffee cv. Catuaí with naturally occurring *M. exigua* in a randomized block design in an 8x6 factorial scheme (8 treatments × 6 collection periods) with four blocks in a total of 192 plots, each consisting of eight plants, of which the six central ones were analyzed. Before applying the treatments, the initial population (Pi) of *M. exigua* was quantified. The other collections were carried out at 120, 180, 240, 360 and 420 days after application of the products (D_AA_). The relative efficiency (RE) of the treatments was also calculated. For the number of nematodes in the roots, the treatments that most reduced the population of *M. exigua* were T3 (47.1); T4 (49.12); T5 (48.32); T6 (48.88) and T7 (50.91), but T1 and T2 were also efficient. For the reduction of the nematode in the soil, T6 was more effective, although all of them reduced the population. The RE ranged from 34.77% at T1 to 45.04% at T3, emphasizing that, except for T1 and T2, all treatments presented RE above 40%. In the evaluation at 180 D_AA_, there was a reduction of the nematode in the roots. In the collections carried out at 240 D_AA_ and 360 D_AA_, there was an increase in the population of *M. exigua*; however, after this period, at 420 D_AA_, there was once again a reduction in the population of the nematode. The highest yields in 2019 were observed in plants treated with T1 (53 sc/ha) and T5 (52.75 sc/ha), whereas in 2020 it was in plants that received T2 (54.5 sc/ha), T6 (58.25 sc/ha) and T7 (62.12 sc/ha). In 2019, except for T6, all treatments resulted in increased productivity and, consequently, profit for the coffee grower, with emphasis on T1 and T5. In 2020, the treatments that brought the most profit to the coffee grower were T6 and T7.

## The effect a *pseudomonas* strain of bacteria isolated from soybean nodules on root-knot egg hatch and juvenile mortality

Ali, Md. Sahadat, F. T. Z. Mony and J. D. Eisenback

School of Plant and Environmental Science, Virginia Tech, Blacksburg, VA 24061

### Abstract

Soybeans are economically important in the U.S. because they are used for food, feed, and export. Root-knot nematodes (RKN) (*Meloidogyne* spp.) harm soybean roots and reduce crop yields which increases the costs of farming. The search for tactics to reduce the effect that nematodes have on crop production necessitates the exploration of novel, effective microbial control agents. This study investigated a *Pseudomonas* strain of bacteria isolated from soybean nodules for its potential as a microbial pesticide. Its ability to suppress egg hatch and reduce the viability of infective second-stage juveniles (J2) was evaluated *in vitro*. Over 21 days, egg hatch was assessed at 1, 3, 4, 6, 8, 12, 15, 18, and 21 days, and revealed that this bacterium suppressed hatch by more than 90% as compared to the control. In a separate experiment conducted over 6 days, it caused 99% mortality of J2 in the first 72 hr. as compared to the control. These findings accentuate the potential of this organism as a biocontrol agent that can be applied as a seed treatment and serve as a sustainable alternative to chemical nematicides. Additional studies to determine the biological mechanisms underlying the suppression of RKN by this bacterium are currently underway.

## Exploring novel immunomodulatory pathways between parasitic nematode excretory/secretory proteins and mammalian host-parasite interactions

Anesko, Kyle^1^, Y. He^2^, J. Jennett^2^, P. Azizpor^1^, A. K. Lima^1^, M. Nair^2^ and A. R. Dillman^1^

^1^Dept. Nematology, University of California, Riverside, CA

^2^Division of Biomedical Sciences, School of Medicine, University of California, Riverside, Riverside, CA

### Abstract

Host-parasite interactions represent a dynamic interplay between the immune system of the host and the strategies employed by parasites to establish infection. We investigate the role of excretory/secretory proteins (ESPs) released by parasitic nematodes, including *Steinernema carpocapsae’s* ShK-domain (Sc-ShK-2 Domain) and *Heligmosomoides polygyrus’s* FAR protein (Hp-FAR-1), in regulating host immune responses. By focusing on the interaction between nematode ESPs and mammalian immune cells, we aim to elucidate the mechanisms by which these proteins modulate immune cell function and to determine host specificity. Through immunoassays such as the LEGENDplex inflammation panel, we assess changes in cytokine profiles and immune cell activation in response to nematode ESPs. Furthermore, we explore the impact of nematode ESPs on the interaction between bone marrow derived macrophages (BMMacs) and *Nippostrongylus brasiliensis* (Nb) larvae. We anticipate that our findings will contribute to a more comprehensive understanding of host-parasite interactions, with implications for the development of novel therapeutic strategies targeting nematode-borne diseases. By elucidating the immunomodulatory role of nematode ESPs, our research opens new avenues for exploring the complexities of nematode pathogenesis and host immune evasion mechanisms.

## Mobility and rootzone retention of Reklemel™ active in a sandy loam soil is a function of its unique combination of lipophilicity and aqueous solubility

Baidoo, Richard^1^, B. Bangel^1^, T. Thoden^2^ and R. Athalye^1^

^1^Corteva Agriscience, Indianapolis, IN 46268

^2^Corteva Agriscience, 81677 Munich, Germany

### Abstract

The movement and distribution of a nematicide in soil not only impacts its performance (efficacy) and dissipation, but also ground water leaching potential - a critical consideration in making management recommendations to growers with respect to irrigation regimes, application volumes, and frequency of application. In this study, a soil column assay was used to investigate relative mobility and rootzone retention of Reklemel™ Active and a number of modern nematicides including Abamectin, Fluensulfone, Fluopyram, and Oxamyl. These nematicides were chosen due to their varying physico-chemical properties: thermodynamic water solubility, octanol-water partition coefficient (logD), soil adsorption coefficient (KOC), and molecular weight. The active ingredient was dissolved in 5% acetone in water and applied to soil column surface and irrigated with high volume simulated rainfall to allow the chemistries to move down the soil column filled with a sandy loam soil at field capacity. Both analytical and biological approaches were used to race the concentrations of the chemistries along the soil column. Water samples were taken from various sections of the soil column using lysimeters and from the leachates for chemical analysis by HPLC. The movement of the active ingredient was also traced using a novel soil bioassay in which cucumber plants were over-infested with the root-knot nematode, *Meloidogyne incognita* along the column. The results showed that Abamectin was the least mobile chemistry and Oxamyl was the most mobile chemistry. Fluensulfone and Fluopyram possessed intermediate levels of mobility whereas Reklemel demonstrated a well-balanced behavior between mobility and rootzone retention along the soil column, due to its combined properties of solubility, logD, KOC, and molecular weight. These physico-chemical properties of Reklemel allow for both optimal mobility through the soil (high solubility) as well as adequate binding (sorption) to soil particles along the soil profile (lipophilicity). The biological response data corroborated the analytical data, i.e. good nematode control was seen all along the soil column for Reklemel. The study shows chemical behavior and distribution in the soil around crop roots can be a differentiating factor for nematicide performance.

## NemaTaxa: bridging the gap in nematode community data analyses

Baker, Hannah^1^, J. R. Ibarra Caballero^2^, C. Gleason^1^, C. E. Jahn^2^, C. N. Hesse^3^, J. E. Stewart^2^ and I. A. Zasada^3^

^1^Department of Plant Pathology, Washington State University, Pullman, WA 99163

^2^Department of Agricultural Biology, Colorado State University, Ft. Collins, CO 80523

^3^Horticultural Crops Research Unit, USDA-Agriculture Research Service, Corvallis, OR 97330

### Abstract

High-throughput amplicon sequencing of nematode communities is increasing our understanding of nematode community ecology. However, one of the problems preventing widespread adoption of metabarcoding in nematode community analysis is the lack of comprehensive sequence databases featuring consistent taxonomic naming conventions. Here, I will introduce the NemaTaxa database. This database was generated using 18S sequence data and is compatible with QIIME and mothur analysis platforms. We evaluated NemaTaxa against existing databases, specifically PR2 and Silva v132, within the mothur framework, and using nematode communities collected from potato cropping systems in Oregon, Idaho, and Washington. NemaTaxa exhibited a better performance than Silva v132 and PR2. Notably, NemaTaxa enabled classification at the genus, family, and order levels, while PR2 often resulted in sequences being assigned only at the class level due to incomplete taxonomic strings. NemaTaxa represents an accessible database solution for nematologists seeking to investigate nematode community ecology.

## How does prolonged exposure to extracts of litchi tomato, *Solanum sisymbriifolium*, impact egg hatch of *Meloidogyne hapla*?

Baker, Hannah V. ^1^, L. Schulz^2^, L. M. Dandurand^2^ and I. A. Zasada^1^

^1^USDA-ARS, Corvallis, OR 97330

^2^University of Idaho, Dept. of Entomology, Plant Pathology, and Nematology, Moscow, ID 83844

### Abstract

The northern root-knot nematode, *M. hapla,* is a problem for potato, wine grape, and vegetable growers in the Pacific Northwest. The primary methods of control for this nematode are pre-plant fumigation and post-plant nematicide application. Identifying more environmentally friendly methods of control is of great importance. A proposed method of control is litchi tomato, *Solanum sisymbriifolium*, which is used globally as a trap crop for *Globodera* spp. and has been shown to reduce populations of *G. pallida* by up to 99% in potato following exposure to litchi tomato. While the infection process of *Meloidogyne* spp. and *Globodera* spp. are quite different, the mechanisms by which litchi tomato interacts with plant-parasitic nematodes are not well understood. It is assumed that at least one mechanism is the production of nematicidal compounds that negatively impact nematode survival. The objective of this project was to determine the impact of litchi tomato extracts on *M. hapla* fecundity, infectivity, and mobility. Compounds from litchi tomato roots or stems and leaves were extracted using different solvents of increasing polarity: hexane, dichloromethane, ethyl acetate, and butanol, respectively. *Meloidogyne hapla* egg hatch was enumerated at 3, 5 and 7 days following exposure to extracts for either 24 or 72 hours. Initial data indicates that eggs exposed for 72 hours experienced decreased hatch rates, up to 20%, when compared to hatch at 24 hours. Successful inhibition of *M. hapla* egg hatching by litchi tomato extracts could provide growers with another tool to manage *M. hapla*. Further experimentation will identify specific compounds in extracts that are responsible for the toxic effects of litchi tomato against plant-parasitic nematodes.

## Callose deposition regulated by PTI modulates potato-nematode interactions

Bali, Sapinder^1^, H. Peng^2^, M. Sobczak^3^ and C. Gleason^1^

^1^Department of Plant Pathology, Washington State University, Pullman, WA 99164

^2^USDA-ARS, San Joaquin Valley Agricultural Sciences Center, Parlier CA 93648

^3^Department of Botany, Warsaw University of Life Sciences, Warsaw, Poland

### Abstract

The root-knot nematodes (RKN’s) cause billions of dollars in crop losses annually. RKN juveniles (J2’s) penetrate the root tissue and establish feeding sites, thus triggering host basal immune responses. To suppress the basal immune responses, the nematodes secrete small molecules/proteins called effectors. Since callose deposition is one of the hallmarks of basal host immunity, nematode effectors suppress it to promote parasitism. In 2017, Gleason et al characterized a nematode effector called Mh265 and showed that the expression of this effector in Arabidopsis enhanced nematode susceptibility and suppressed callose deposition in response to a defense elicitor (Flg22). As a follow up, we are elaborating the role of host callose responses during nematode parasitism in potatoes. Treating potatoes with a callose synthase inhibitor (2-deoxy-d-glucose) significantly enhanced their susceptibility to RKN. Next, we focused on potato Glucan Synthase-like gene (*StGSL05*) gene due to its characterized role in PTI-based callose deposition in Arabidopsis. We developed two independent *StGSL5* knockdown lines in nematode susceptible Desiree potato using RNAi gene silencing. These lines show significantly reduced *GSL05* expression and callose deposits in response to elicitor FlgII28. Nematode assays showed that the J2 penetration and gall count is significantly higher in the *GSL05* RNAi lines as compared to the untransformed potato. Intriguingly, there is no significant difference in the number of egg masses or total egg count between the *GSL05* RNAi lines and untransformed potato. We also performed microscopic (TEM) analyses of the nematode infected roots at 14dpi and found that the giant cells are significantly smaller in the *GSL05* RNAi lines, and the cell walls show signs of degradation as compared to the untransformed roots. We hypothesize that less callose deposition during the early infection stage (PTI-based) leads to more J2’s penetrating the root tissue and establishing the feeding sites but as the infection proceeds lower expression of *GSL05* generates downstream effects that interferes with the giant cell development/integrity.

The aim of the study is to leverage the acquired information to manipulate the deposition of callose or other natural defenses, to develop nematode control strategies tailored for economically important crops like potato.

## Investigating *Meloidogyne chitwoodi* effectors towards enhanced potato crop protection

Bali, Sapinder^1^, I. Ko^1^, M. Teixeira^1^, P. Vieira^2^, T. Maier^3^, T. Baum^3^ and C. Gleason^1^

^1^Washington State University, Dept. Plant Pathology, Pullman, WA 99164

^2^USDA-ARS, Beltsville, MD 20705

^3^Dept. Plant Pathology, Entomology and Microbiology, Iowa State University, Ames, IA 50011

### Abstract

*Meloidogyne chitwoodi*, a root-knot nematode prevalent in potato-growing regions of the Northwestern USA, poses a significant threat to potato crops. Its ability to infect both potato roots and tubers results in unsightly blemishes, diminishing the market value of the produce. Understanding the mechanisms behind *M. chitwoodi’s* successful invasion of potato plants is crucial for developing effective control strategies. To establish a compatible interaction with its host, the nematode secretes small molecules called effectors. These effectors play key roles in suppressing host defense mechanisms and/or helping to form nematode giant-cells, which are essential for parasitic success. While *M. chitwoodi* has hundreds of predicted secreted proteins, their specific functions in parasitism remain largely unknown. Our research aims to unravel the mystery surrounding *M. chitwoodi* effectors. Leveraging a combination of *M. chitwoodi* gland library and whole J2 transcriptome data, we’ve pinpointed novel putative effector genes. Further exploration led us to characterize two of these candidate effectors in greater detail. In situ hybridization assays using antisense probes revealed the localization of these two effector transcripts within the nematode’s gland region, confirming our findings from the gland library data. Moreover, when these genes were expressed in Arabidopsis, the resulting transgenic plants exhibited enhanced susceptibility to root-knot nematode infection, underscoring the significance of these effectors in promoting parasitism. Additionally, we investigated whether these effectors possess the capability to suppress basal plant immune responses. Remarkably, one of the effectors demonstrated the ability to inhibit elicitor-induced callose deposition, indicating its role in defense suppression. This effector data offers insights into the intricate mechanisms by which nematode secretions facilitate parasitism.

## Irreversible effects of reklemel™ on root-knot nematode (*Meloidogyne incognita*) motility, mobility, and infectivity in soil

Baidoo, Richard^1^, B. Bangel^1^, T. Thoden^2^ and C. Geng^1^

^1^Corteva Agriscience, Indianapolis, IN 46268

^2^Corteva Agriscience, 81677 Munich, Germany

### Abstract

The effect of a nematicide on nematodes is a function of its concentration and exposure time. The higher the concentration and longer the exposure time, the more effect is expected.

Such an effect may either be reversible or irreversible which is also dependent on dose and length of exposure. For reversible effects, the nematode can recover from the nematicide impact if the exposure time is short-lived and for irreversible effects, the nematode cannot recover, and the effects continue even when the nematicide is removed. In this study, we demonstrate the irreversible effects of Reklemel™ Active on root-knot nematode (*Meloidogyne incognita*) at sub-lethal concentrations relative to other nematicides (Fluopyram, Fluensulfone, Oxamyl, and Abamectin) using a Phenalysys imaging technology. Second stage juveniles (J2s) of root-knot nematode (RKN) were exposed to different sublethal concentrations of the nematicides and the mobility or motility of the nematodes were assessed using a Phenalysys imaging system at different time points. The J2s were washed of the nematicide, and mobility/motility was reassessed 1 hour and 24 hours after wash to determine recovery. The results showed that the effect of Reklemel was irreversible and continued to immobilize the nematode after washing. We also evaluated the effect of Reklemel on RKN mobility and infectivity relative to other nematicides. The nematodes were washed after exposure to sublethal concentrations for varying time points and were placed on a filter to allow active nematodes to swim through in a modified Baermann funnel system and then inoculated into pots with young cucumber plants to assess nematode infectivity in soil. The results showed that Reklemel at sublethal concentrations (≤ 5 ppm) could significantly reduce RKN infectivity in soil even though the RKN may be alive and could pass through coffee filter. The irreversible effects of Reklemel and its ability to reduce RKN infectivity at sublethal doses are significant attributes that differentiate it from other nematicides on the market such as Abamectin, Fluopyram, or Fluensulfone.

## NemaTaxa: bridging the gap in nematode community data analyses

Baker, Hannah^1^, J. R. Ibarra Caballero^2^, C. Gleason^1^, C. E. Jahn^2^, C. N. Hesse^3^, J. E. Stewart^2^ and I. A. Zasada^3^

^1^Dept. Plant Pathology, Washington State University, Pullman, WA 99163

^2^Dept. Agricultural Biology, Colorado State University, Ft. Collins, CO 80523

^3^Horticultural Crops Research Unit, USDA-Agriculture Research Service, Corvallis, OR 97330

### Abstract

High-throughput amplicon sequencing of nematode communities is increasing our understanding of nematode community ecology. However, one of the problems preventing the widespread adoption of metabarcoding in nematode community analysis is the lack of comprehensive sequence databases featuring consistent taxonomic naming conventions. Here, I will introduce the NemaTaxa database. This database was generated using 18S sequence data and is compatible with QIIME and mothur analysis platforms. We evaluated NemaTaxa against existing databases, specifically PR2 and Silva v132, within the mothur framework, and using nematode communities collected from potato cropping systems in Oregon, Idaho, and Washington. NemaTaxa exhibited a better performance than Silva v132 and PR2. Notably, NemaTaxa enabled classification at the genus, family, and order levels, while PR2 often resulted in sequences being assigned only at the class level due to incomplete taxonomic strings. NemaTaxa represents an accessible database solution for nematologists seeking to investigate nematode community ecology.

## Delving into *Steinernema* and *Heterorhabditis* biology through hybrid genome assembly

Baniya, Anil^1^, E. M. Schwarz^2^ and A. R. Dillman^1^

^1^Dept. of Nematology, University of California, Riverside, Riverside, CA 92521

^2^Dept. of Molecular Biology and Genetics, Cornell University, Ithaca, NY 14853

### Abstract

Nematodes show extraordinary versatility in the evolution of parasitic lineages, with both animal-parasitic and plant-parasitic branches arising independently many times within the phylum. The genomes of entomopathogenic nematodes (EPNs) are critical for understanding the molecular mechanisms that drive parasitism, symbiosis, and host-parasite interactions. To gain a better understanding of parasitism evolution and its biology, we sequenced and assembled the genomes of several species within the insect parasitic *Steinernema* and *Heterorhabditis* genera, which have been extensively researched for decades for their roles in biological control against agricultural insect pests and as models for animal parasites. Here we present new draft genome sequences for *Heterorhabditis indica* (Contig N50 ~1.1 Mb; total size 68.5 Mb), *Steinernema scapterisci* (N50 ~4.0 Mb; scaffold N50 ~ 15.1 Mb; total size 77.5 Mb), and *Steinernema glaseri* (Contig N50 ~1.7 Mb; scaffold N50 ~16.2 Mb; total size 82.8 Mb). A high-quality genome assembly is a valuable tool for conducting a wide range of analyses, such as genome completeness assessments, gene number predictions, chromosomal synteny comparisons, evaluations of the conservation of parasitism-associated genes, and orthologous gene comparisons, to gain deeper insights into nematode biology.

## Lisi global’s directed energy system: a non-chemical approach for the management of nematodes in nurseries

Benedetti, Tatiana^1^, I. Zasada^2^, M. Moretti^1^, J. Weiland^2^, K. Graham^2^ and J. Crisp^3^

^1^Dept. Horticulture, Oregon State University, Corvallis, OR 97330

^2^USDA-ARS Horticultural Crops Disease and Pest Management Research Unit, Corvallis, OR 97330

^3^Lisi Global Inc, Richland, WA 99353

### Abstract

Oregon leads the United States in the production of several ornamental nursery crops including shade trees, conifers, and flowering trees. Nematodes are a major constraint to tree seedling production for the ornamental industry. Failure to control can result in seedling death or unintentional distribution of infected nursery stock. One of the primary ways to control nematodes is through pre-plant fumigation with methyl bromide. However, the use of this chemical is detrimental to the environment and human health, making it urgent to develop safer alternatives. Our long-term goal is to discover new ways to manage nematodes that will reduce reliance on soil fumigants. The Directed Energy System (DES) is a novel alternative management technique that generates pulses of electricity capable of killing nematodes. Controlled experiments were conducted against two life stages of *Meloidogyne chitwoodi* [egg and second-stage juveniles (J2)] and mixed life stages of *Pratylenchus neglectus* to determine the electrical parameters required to kill each nematode. To evaluate the parameters to kill *M. chitwoodi* eggs, 1,000 eggs were added to pots containing soil, and different energy levels were applied. After treatment, tomato seedlings were transplanted in each pot and grown for eight weeks to determine nematode survival. To determine the effects on *M. chitwoodi* J2 and *P. neglectus,* nematodes were added to soil in pots, and different energy levels were applied. Nematode survival was determined by extracting living nematodes using the Baermann funnel method. The energy required to kill 90% of *M. chitwoodi* eggs was 23.4 J/cm^3^ of soil. A higher level of energy, 110 J/cm^3^, was required to kill 100% of *M. chitwoodi* J2. *Pratylenchus neglectus* density decreased linearly with increasing energy levels; at 30 J/cc^3^ of soil, there was a 100% kill of *P. neglectus*. Preliminary data suggests that both voltage and frequency significantly affect nematode viability in the soil. High voltage kills nematodes and the higher the frequency the more lethal the treatment.

## Pathotypes of *Globodera* species from Peru to assess diversity of South American populations

Bhatta, Bhupendra^1^, I. A. Zasada^2^, J. Kuhl^3^, J. Franco^4^ and L. M. Dandurand^1^

^1^Department of Entomology, Plant Pathology and Nematology, University of Idaho, Moscow, Idaho 83844-2329, USA

^2^USDA-ARS Horticultural Crops Disease and Pest Management Research Unit, Corvallis Oregon 97330

^3^Department of Plant Sciences, University of Idaho, Moscow, Idaho 83844-2333, USA

^4^Agro Innovation-Perú, Valle Hermoso, Surco, Lima, Peru

### Abstract

The potato cyst nematodes, *Globodera pallida* and *Globodera rostochiensis*, are globally regulated potato pests. In the U.S., *G. rostochiensis* was first detected in New York in 1941 and *G. pallida* was detected in Idaho in 2006. Phytosanitary guidelines to control and contain these two species are in place in both New York and Idaho. Potato varieties resistant to *G. rostochiensis* are available for New York growers, however, resistance to *G. pallida* is not available in russet potatoes, the primary market class grown in Idaho. Control of *G. pallida* involve soil fumigation and currently, efforts to develop *G. pallida* resistant russet potato varieties are underway. Several pathotypes of potato cyst nematodes have been characterized based on their ability to multiply on differential clones containing different resistant genes. An The European pathotyping system has characterized three *G. pallida* pathotypes (Pa1-Pa3) five *G. rostochiensis* pathotypes (Ro1-Ro5). Six *G. pallida* pathotypes and four *G. rostochiensis* pathotypes have been characterized by a South American system. The center of evolution for potato cyst nematodes is in the highlands of the Andes. This research aims to evaluate the genetic diversity of the *Globodera* species by identifying *Globodera* species collected from potato areas in the in the Andean highlands of Peru and assessing their pathotype. Of the 10 populations evaluated, six populations were identified, using multiplex PCR, as *G. pallida,* one as *G. rostochiensis* and three as a mix of both species. To determine the pathotype, we conducted phenotypic assays on differential clones. The different resistance genes are used to distinguish between *Globodera* species with different virulence genes. After 8 weeks of growth, roots were stained with acid fuchsin, and all life stages of the nematode were counted. Number of females and relative susceptibility score compared to the susceptible potato Désirée were used to determine pathotype of the populations. Our bioassay results suggest that two populations were *G. pallida* Pa2/3, one population was *G. pallida* Pa1, two populations were *G. pallida* Pa3, one population was possibly *G. rostochiensis* Ro2, and one population was an unknown pathotype. Three populations were mixed species and their pathotype could not be determined. These results indicate that *Globodera* populations are more diverse than what is encompassed by the resistance genes that are available to U.S. potato breeders. Obtaining a larger range of genetic resistance sources will be crucial to fully represent this diversity.

## Relationships among temperature stability, nematodes and potato tuber productivity

Bird, George^1^, B. Basso^1^, R. Price^1^, M. Otto^2^ and F. Warner^1^

^1^Michigan State University, East Lansing, MI

^2^Agri-Business Consultants Inc., Lansing, MI

### Abstract

Relationships among temperature stability, nematodes and potato tuber productivity were evaluated in 2019 in two adjacent Michigan commercial potato fields (P01 and P38). Temperature stability zones for the fields were created using the protocol of Maestrini and Basso (Field Crops Res. 219:106–112). The procedure involved flying a drone with thermal sensors over each field six times, with the initial flight taking place before potato planting and the final flight immediately before the predicted tuber harvest date. Temperature was classified as four stability zones: cold/stable, medium/stable, hot/stable, and unstable. Within-field areas identified as hot/stable were sites where temperature is consistently five degrees Celsius greater than areas classified as cold/stable. Soil texture maps and P01 and P38 scouting experiences were used to geo-position twelve sites in each field for hand dug potato tuber yield determination and collection of soil for nematode and soil health indicator analyses. The sites were selected to represent a diversity of within-field soil textures and yield history. The soil heath indicator analyses were done at the Cornell University Soil Health Laboratory and nematode identification by the MSU Nematode Diagnostic Laboratory. It was previously reported that potato tuber yields, nitrogen mineralization potential, active carbon, and the Cornell Soil Health Index in Michigan potato fields in 2017 and 2018 were significantly greater in cold/stable areas, compared to hot/stable sites. This was also true for P38. In P01, however, tuber yields were low at all three temperature stability zones and population densities of *Pratylenchus penetrans* were significantly greater in the hot/stable areas compared to cold/stable areas. In P38, population densities of a *Tylenhorhynchus* sp., bacterivores and total nematodes were greater in the cold/stable areas, compared to hot/stable sites. *Meloidogyne hapla* was common in all temperature stability zones in P01, but not detected in P38. While temperature and moisture are key driving variables in most ecosystems, much remains to be learned about their impacts on nematodes under both natural and human-managed conditions.

## Overcoming resistance: unraveling the mechanisms behind root-knot nematode evasion of tomato *Mi*-gene

Blundell, Alison Coomer^1^, P. Shakya^1^, M. Winter^2^, D. Lunt^2^, V. M. Williamson^1^ and S. Siddique^3^

^1^Dept. of Plant Pathology, University of California Davis, Davis, CA

^2^School of Nature Natural Sciences, University of Hull, Hull, UK

^3^Dept. Entomology and Nematology, University of California Davis, Davis, CA

### Abstract

Root-knot nematodes (RKNs) are among the most devastating pathogens of crops, causing substantial yield and economic losses worldwide. These parasitic organisms can infect over a hundred different plant species and can evade plant defense mechanisms by secreting a concoction of effectors. For decades, the *Mi-1* resistance gene has been effective in detecting and inhibiting RKNs in tomatoes. However, the underlying mechanisms by which *Mi-1* detects these pathogens remain largely unknown. In recent years, resistance-breaking populations have emerged in both greenhouse and field settings, posing a threat to the potency and effectiveness of the *Mi-1* gene and, consequently, the tomato industry. We used two strains of *M. javanica*, one strain VW4, which is recognized by *Mi-1*, and another strain, VW5, which was selected from VW4 and can overcome resistance mediated by *Mi-1*. Utilizing the newly constructed reference genome for *M. javanica* (VW4), we compared genomes of VW4 and VW5 and identified an approximately 50 kb region that is present in VW4 but missing in VW5. This missing region contains seven protein-coding genes, three of which encode putative effectors and are currently being tested as potential avirulence genes for *Mi-1*. In addition, we have conducted a series of infection assays on different host plants lacking *Mi-1,* and the results revealed a significantly lower egg count in VW5 when compared to VW4. We plan to expand these assays by testing additional *M. javanica* resistance-breaking strains collected from fields all over California to determine if this trade-off is consistent across other strains. Overall, our results suggest that although VW5 can overcome *Mi-1*, there is a trade-off in the form of compromised reproduction. This research helps to better understand the mechanism and components of *Mi-1* and develop strategies for addressing resistance-breaking populations.

## Discrepancies between morphological and molecular diversity in an Antarctic nematode

Borgmeier, Abigail and B. Adams

Brigham Young University, Provo, UT

### Abstract

*Eudorylaimus* is one of five nematode genera that are found in the Antarctic Dry Valleys. Despite being one of only a handful of nematode taxa present in the region, there have been a limited number of studies characterizing the diversity of *Eudorylaimus* in the Dry Valleys. It is possible that there is more than one species present within the Dry Valleys after examining the degree of molecular diversity within the genus in Antarctica. The first objective of this study is to examine the degree of molecular diversity within Antarctic *Eudorylaimus* as compared with other *Eudorylaimus* species within the *Eudorylaimus* phylogeny. The second objective of this study is to resolve the *Eudorylaimus* genus phylogeny to find evidence for which *Eudorylaimus* taxa is sister to the Antarctic Dry Valleys *Eudorylaimus* species. All available *Eudorylaimus* sequences from GenBank and the Barcode of Life Datasystems (BOLD) were downloaded. The COI mitochondrial genes, 28S genes, and the 18S small ribosomal subunit genes of any species of *Eudorylaimus* were downloaded and included in the dataset. *Eudorylaimus* specimens were collected from the McMurdo Dry Valley region and the 18S and COI loci were sequenced. Following phylogenetic analysis using maximum likelihood trees, the Antarctic *Eudorylaimus* clade shows that there is very low genetic diversity within Antarctic *Eudorylaimus* taxa, even though these taxa are from distant locations. There is a large divergence between Antarctic *Eudorylaimus* and all other *Eudorylaimus* species, making it impossible to determine the sister taxa to Antarctic *Eudorylaimus* without further sampling. While there is high morphologic variation within this region, there is low genetic variation within the selected loci, making it unique compared to other Antarctic nematodes with high genetic variation and population structure.

## Developing a database and analysis pipeline for soil nematode abundance and functional group composition in Hawaii

Braley, Lauren and K.-H Wang

Department of Plant and Environmental Protection Sciences, University of Hawaii at Manoa, Honolulu, HI 96822

### Abstract

Nematodes are the most abundant animals globally, making up approximately four-fifths of the abundance of animals on land, and playing key roles in soil ecosystem functioning at different trophic levels. Their distinct morphological features, especially their mouthparts, allow for ease of identification and classification of nematodes into trophic groups. Thus, nematodes have been considered good indicators of biological functions occurring in soil systems. Additionally, knowledge of nematode trophic abundance in relation to geographical location can provide insight when approaching climate change modeling and environmental decision-making. In order to produce accurate models, large baseline datasets are needed. Previous work has generated a global soil nematode and functional group abundance database and model. However, the database is limited and missing significant tropical data. Hawaii has one of the most diverse ecosystems worldwide, with diverse soil orders present, and a large amount of soil nematode community data collected over several decades that has yet to be included in previous global soil nematode abundance datasets. The objective of this project was to 1) curate a database of pre-existing nematode trophic data in Hawaii, 2) assess soil nematode trophic abundance across Hawaii in relation to the global soil nematode database, and 3) examine the relationship between Hawaii nematode trophic abundance with various climate relevant variables. Nematode trophic data was compiled and analyzed similar to methods previously described in the study conducted by van den Hoogen and colleagues (2019; https://doi.org/10.1038/s41586-1019-1418-6). After aggregating sample data into unique ‘pixels’, environmental global databases were sampled at each pixel location to compare nematode trophic abundance to other environmental variables, including various soil properties, and vegetative, climatic, topographic, and anthropogenic information. A total of 1,213 samples were collected from five of the six major islands in Hawaii (Oahu, Maui, Big Island, Kauai, Molokai), and aggregated into 21 unique pixel locations. Nematode trophic abundance data from Hawaii followed similar trends seen in previous global abundance data, with the greatest relative abundance observed in bacterivores and the lowest in omnivores and predators. However, total mean nematode abundance for Hawaii was two times greater than previously reported means for tropical biomes elsewhere. When comparing data between islands, overall highest nematode abundance was observed on Molokai and the lowest mean abundance was observed on the Big Island, but interestingly, highest predator abundance was observed on the Big Island while the lowest was on Molokai. When comparing Hawaii nematode abundance to environmental properties, observed soil organic carbon content and pH showed impact, whereas climate factors (e.g. temperature and precipitation) did not. Based on these findings, the current global model differs greatly from soil nematode abundance and climate relationship trends observed in Hawaii, demonstrating the need for inclusion of additional tropical nematode abundance data to improve the accuracy of global nematode distribution models as future environmental decision-making tools.

## Heat tolerance of distinct geographic populations of *Meloidogyne incognita* is possessed by intrinsic thermal acclimation reaction: nematode response to global warming

Hada, Alkesh^1^, P. Bucki^1^, N. Sela^2^ and S. B. Miyara^1^

^1^Department of Entomology, Nematology and Chemistry Units, Agricultural Research Organization (ARO), The Volcani Center, Bet Dagan 7505101, Israel

^2^Department of Plant Pathology and Weed Research, Agricultural Research Organization (ARO), The Volcani Center, Bet Dagan 7505101, Israel

### Abstract

Research interest in the mechanisms enabling plant-parasitic nematodes to adjust their physiological performance and cope with changing temperatures has intensified in light of global warming. Here, we show that geographically distinct populations of the root-knot nematode *Meloidogyne incognita*, prevalent in the three main pepper-growing regions in Israel—Carmel Valley (Carmel), Jordan Valley (JV), and the Arava Rift (Arava)—possess persistent differences in their thermal acclimation capacity, which affect embryonic development. The optimal temperature for embryonic growth completion for the Carmel population was 25°C, 25 and 30°C for the JV population, and 30°C for the Arava population. Juvenile survival indicates that at the lowest temperature tested at 20°C, compared with the Arava population, Carmel population gained the highest survival rates throughout the experimental duration, while at 33°C, Arava population gained the highest survival rate throughout time duration. This tendency was further reflected by roots penetration assay as well as whole plant infection studies, while Carmel population demonstrated increased J2s penetration at 25°C compared to the JV and Arava populations, the opposite trend was found at 33°C, where increased penetration of tomato roots was observed for the Arava population compared to the Carmel and JV populations. Next, for studying the molecular mechanism underlying population’s thermal adaptation, we performed transcriptomic analysis. RNA of infected roots at 25, 30, and 33°C was extracted and sequenced using Illumina sequencing PE150, which identified an extensive set of genes in different populations cultured under different temperatures. Gene ontology and KEGG-pathway-based analysis were used to further investigate the functions of the differentially expressed genes (DEGs). Results so far indicate that Carmel population possess higher genetic plasticity compared with Arava, as reflected by significant increase in differential expressed genes along the temperatures. The transcriptome profiles presented in this study provide insight into the transcriptome complexity and will contribute to further understanding the thermal adaptation and help to predict eco-evolutionary trends under temperature change scenarios.

## Sensitivity of *Meloidogyne incognita* and *Rotylenchulus reniformis* to cyclobutrifluram

Katherine Brown and T. R. Faske

University of Arkansas System, Division of Agriculture, Lonoke Extension Center, Lonoke, AR 72086

### Abstract

Cyclobutrifluram, a succinate dehydrogenase inhibitor fungicide, is being evaluated as a seed-applied nematicide in cotton and soybean. Currently, there is no information on the sensitivity of *Meloidogyne incognita* or *Rotylenchulus reniformis* to cyclobutrifluram. Second-stage juveniles of *M. incognita* and mixed life stages of *R. reniformis* were exposed to aqueous solutions of cyclobutrifluram. Paralysis was observed for both nematode species after 2 hr of contentious exposure. The 2-hr EC_50_ values were 0.48 μg/ml and 1.07 μg/ml for *M. incognita* and *R. reniformis*, respectively. Recovery in nematode motility was observed for both nematode species after 1 hr of being removed and rinsed from a 1 hr exposure time to their respective 2-hr EC_50_ values. However, exposure for 1 hr to concentrations of cyclobutrifluram below their respective 2-hr EC_50_ values did inhibit both nematode species from infecting tomato roots. These data provide some information on the sensitivity of two nematode species to a new nematicide, cyclobutrifluram.

## Effects of cover cropping systems on soil nematode community composition in organic vegetable production

Budhathoki, Sabina^1^, Z. J. Grabau^1^ and G. Maltais-Landry^2^

^1^Department of Entomology and Nematology, University of Florida, Gainesville, FL, 32611

^2^Department of Soil, Water and Ecosystem Sciences, University of Florida, Gainesville, Florida, 32603

### Abstract

Nematode communities are integral to biological soil health and can be affected by diverse cropping practices such as cover cropping. While some cover crops can serve as hosts, others may inhibit nematode populations. Although cover crops are used as a measure to suppress plant-parasitic nematodes (PPNs), the impact of cover crop mixtures relative to monocultures is less certain. Two field trials were conducted in 2021 and 2022 to determine the effects of different cover cropping systems on soil nematode communities preceding three vegetables: squash, bok choy and pepper production in an organic vegetable production system. The experiment was arranged in randomized complete block design with 4 replications. The treatments included monoculture i) sunn hemp (SH), two bicultures of ii) SH and sorghum-sudangrass (SSG), and iii) SH and browntop millet (M), a high diversity mixture of iv) SH+SSG+M+ cowpea (CP) compared to v) weedy fallow. Cover crops were terminated after approximately two and a half months, and soil nematode communities, comprising both free-living and PPNs were evaluated after cover crop termination and at vegetables harvest. In both years, cover crops had similar effects on the nematode community in each vegetable crop, so results are pooled across vegetable crops. The effects of cover crops on the nematode community were not different at vegetable harvest, thus the results focus on the effects observed after the termination of cover crops. In 2021, PPN infestation at the site was low, consisting of very low abundances of economically important PPNs. However, in 2022, SH monoculture reduced PPNs including stubby-root nematodes (*Paratrichodorus* spp), root-knot nematodes (*Meloidogyne* spp.) and ring nematodes (*Mesocriconema* spp) compared to other cover crop treatments. Moreover, SH+SSG or the high diversity mixture increased stubby-root nematode populations compared to SH monoculture or weedy fallow. In both years, all cover crop treatments significantly increased bacterivores and fungivores compared to weedy fallow except that SH+M reduced bacterivores and fungivores compared with all other treatments in 2022. Omnivores and predatory nematode abundances were not significantly different across cover crop treatments in 2021, but in 2022, SH+SSG and the high diversity mixture increased populations compared to other treatments. In summary, the results suggested that SH monoculture as well as bicultures and diverse mixtures, can impact soil nematode communities, with SH showing potential in reducing PPNs. Additionally, the inclusion of species like sorghum-sudangrass and millet in the mixture may elevate stubby-root nematodes in soil.

## Efficacy of fluopyram and fluensulfone on sting nematode (*Belonolaimus longicaudatus*) in florida strawberry

Bui, Hung and J. Desaeger

University of Florida, Department of Entomology and Nematology, Gulf Coast Research and Education Center, Wimauma, FL 33598

### Abstract

Florida accounts for more than 90% of winter strawberries grown domestically in the United States. Sting nematodes (*Belonolaimus longicaudatus*) are ubiquitous in Florida strawberry fields and are considered one of the major overall pests. For decades fumigants were the only nematicides available to strawberry growers as no non-fumigants were labeled for strawberries. However, in the past 5–10 years, two non-fumigant nematicides fluensulfone (Nimitz^®^) and fluopyram (Velum^®^) have become registered for use in strawberry production in Florida. While these products should not be considered direct replacements for fumigants, they do offer more selective and less toxic options for strawberry growers to manage sting and other nematodes. Between 2018–2022, we conducted four field experiments evaluating these new nematicides at the research farm of the University of Florida Gulf Coast Research and Education Center, Wimauma, FL. The experiments were conducted using a complete randomized block design with five replicates per treatment at commercial rates. The nematicide treatments were: (1) fluensulfone treated a week before transplanting, (2) fluopyram treated at transplanting, (3) fluensulfone treated a week before transplanting and followed by fluopyram treated at three weeks after transplanting, (4) a cocktail of fluensulfone and fluopyram treated a week before transplanting, (5) metam potassium fumigation three weeks before transplanting and (6) untreated control. Strawberry fruits were harvested weekly from November-March and nematode soil population density was evaluated at the middle and the end of the season. Metam potassium consistently showed higher yields than the untreated control which was irrespective of its effect on sting nematode soil population densities which were often higher at the end of the season. Fluopyram and fluensulfone affected yields to a lesser degree but especially fluopyram did show a trend towards lower sting nematode soil population densities at the end of the season. In the 2018–2019 season, total yields were not different among treatments, but sting nematode soil populations in fluensulfone and fluopyram treatments were numerically lower than in the untreated control. In the 2019–2020 and 2020–2021 seasons, total yields were greater with fluopyram, and combinations of fluopyram and fluensulfone as compared to the untreated control. This trend was also observed in the 2021–2022 season in the fluopyram treatment in terms of yields and sting nematode soil population density. In conclusion, the fumigant treatment with metam potassium protected strawberry yield the most, probably by providing not only nematode control, but also disease and weed control, However, the fumigant did not reduce sting nematode populations at the end of the season. Fluensulfone, and especially fluopyram often reduced sting nematodes more and could offer an alternative or additional tool for nematode management in strawberries.

## Pathogenic hitchhikers: investigating the synergy of bacteria and nematodes on plant health

Casey, Veronica^1^, M. D. arter^2^, C. Allen^2^, J. C. Hong^3^, T. Lowe-Power^4^ and S. Siddique^1^

^1^Dept. Entomology and Nematology, University of California, Davis, Davis, CA

^2^Dept. Plant Pathology, University of Wisconsin-Madison, Madison, WI

^3^USDA ARS United States Horticultural Research Laboratory, Fort Pierce, FL

^4^Dept. Plant Pathology, University of California, Davis, Davis, CA

### Abstract

In the vast scope of soil ecology, plant-parasitic nematodes can forge alliances with other microbial adversaries, such as the disease complex formed between nematodes and bacterial wilt-causing *Ralstonia* spp. These disease complexes exacerbate disease symptoms and yield losses. Plant-parasitic nematodes are microscopic roundworms that cause approximately $100 billion in yield loss a year, and most of the damage is attributed to root-knot nematodes (RKNs; *Meloidogyne* spp.). Bacterial wilt is caused by multiple *Ralstonia* species, namely *Ralstonia pseudosolanacearum, R. solanacearum,* and *R. sygzii* that enter the plant’s roots to colonize its vascular system. Prior to *Ralstonia* infection, RKN infection may facilitate bacterial disease by increasing access to the vascular tissue. However, little research has been conducted to elucidate the molecular details of this interaction. Previous reports of RKN and *Ralstonia* spp. in the field hypothesized that the infection was due to root wounding and physiological changes. In this study, I will determine the nature of the interaction between *Ralstonia* and nematodes at both ecological and molecular levels. This research project will explore the hypotheses that 1) *Ralstonia* adheres to the cuticle of nematodes using specialized appendages called pili and 2) *de novo* xylem formation in the galls increases *Ralstonia* transport into the plant. A common strategy for preventing nematode infection is by using resistant plant cultivars. However, resistance-breaking nematode populations have arisen, and we plan to utilize resistance-breaking nematodes, which are most likely to interact with bacterial wilt in the field. This presentation will report on the attachment and greenhouse experimental results of the RKN-*Ralstonia* complex. The escalation of climate change is leading to increased instances of pathogenicity; therefore, it is crucial to uncover disease complexes which can have monumental consequences on food security. A meticulous study into the nematode and *Ralstonia* disease complex will support the management of these damaging pathogens across the world.

## Cutting the cord: using gene editing to manipulate susceptibility genes in tomatoes against Root-Knot Nematodes

Castro Esparza, Bardo and S. Siddique

University of California, Davis, Department of Entomology and Nematology, Davis, CA, 95616

### Abstract

Plant parasitic nematodes (PPNs) are a major problem for agriculture, causing 8 to 15% of total crop losses worldwide. Root-Knot Nematodes (RKNs) are the most economically devastating PPNs due to their broad host range, infecting about 2,000 different plant species, including important crops like tomatoes. During their obligate biotrophic life cycle, RKNs act as unwelcome guests in root systems, penetrating and migrating to the vascular cylinder where they induce the formation of giant cells (GCs). These GCs have enhanced nutrient uptake and serve as a source of nourishment throughout the remainder of the RKN life cycle. Adult female RKNs produce egg masses containing large numbers of eggs, which can survive in the soil for extended periods and lead to subsequent infections. Currently, there are no ideal strategies to halt RKN infections. Some management strategies include the use of nematicides and resistance genes like *Mi-1*. However, these strategies have limitations, such as increased nematode resistance to nematicides and the emergence of *Mi-1* resistance breaking strains of RKNs. The decreasing efficacy of these traditional management efforts is prompting the exploration of new approaches to develop genetic resistance to RKNs. The goal of this project is to identify plant susceptibility genes (S genes) that are involved in GC maintenance and nutrient uptake in tomatoes. We have performed transcriptional analysis of tomato galls resulting from RKN infection and used these data to identify genes highly upregulated 28 days post-infection, a time point when GCs are fully developed, and nutrient acquisition is pivotal for RKN life cycle. Using this data, we developed a pipeline employing several criteria to identify candidate genes that might be vital for GC maintenance but whose alteration would have minimal effects on plant growth. We will use gene editing techniques to precisely target these candidate susceptibility genes in tomatoes and test their effect on RKN infections. This approach is expected to yield new insights into genes and pathways important for GC establishment and maintenance and might provide new forms of genetic resistance in tomatoes.

## Field evaluation of winter wheat lines for the management of *Pratylenchus neglectus* in Montana

Consoli, Erika^1^, J. O. Eberly^2^ and A.T. Dyer^1^

^1^Montana State University, Dept. of Plant Sciences and Plant Pathology, 59717 Bozeman, MT

^2^Montana State University, Central Ag Research Center, 59642 Moccasin, MT

### Abstract

Root lesion nematodes (RLN, *Pratylenchus* spp.) are important pests of wheat worldwide, causing significant yield loss across much of the globe. For Montana, a leading wheat producing state in the USA, statewide surveys carried out in 2006 and 2007 found 12–15% yield loss due to *Pratylenchus neglectus* infections in winter wheat. At the time, management options were limited to crop rotations involving resistant crop species including peas, lentils, and barley. Unfortunately, their widespread adoption led to the selection of *P. neglectus* pathotypes displaying high multiplication rates on such crops. Because the resistant crops are less profitable than winter wheat and their effectiveness was expected to fail over time, Montana State University started breeding winter wheat lines to incorporate *P. neglectus* resistance into locally adapted cultivars. These efforts ultimately resulted in the development of double haploid lines (DHLs) with significant *P. neglectus* resistance, as determined by greenhouse trials. The objectives of this study were to: i) Validate winter wheat resistance in *P. neglectus* infested fields across the state, and ii) Characterize differences in rhizosphere microbiome associated with nematode susceptible and resistant wheat phenotypes. To accomplish these objectives, eight resistant DHLs, five susceptible DHLs, and three susceptible winter wheat cultivars (i.e. Warhorse, Yellowstone, and Judee) were grown in trials at three *P. neglectus*-infested locations across Montana. For each plot, pre-planting (initial population (Pi)) and post-harvest (final population (Pf)) densities of *P. neglectus* and nematode trophic groups were determined, along with data on agronomic and edaphic factors. While there was a negative correlation between *P. neglectus* densities and yield (*P* < 0.001) across phenotypes and cultivars, significant interactions between wheat phenotypes and Pi were observed for yield (*P* = 0.02), and grain quality (*P* = 0.002). In particular, Warhorse, a solid-stem cultivar popular among Montana growers, displayed a dramatic decrease in yield at higher nematode pressures (*P* = 0.008, 48.8% yield loss at one location) while yields for the resistant phenotypes remained relatively unaffected. Analyses of bacterial and fungal diversity among these lines identified a more diverse rhizosphere community among the resistant DHLs relative to the susceptible DHLs, in which bacterial and fungi alpha diversity was 29.8% and 26.2% greater in resistant DHLs, respectively. To better understand the interactions among *P. neglectus*, wheat phenotypes and the surrounding microbiome, a metagenomic analysis will be performed on select lines per phenotype in the spring 2024. Plots were replanted last fall with the expectation that observed differences between resistant and susceptible entries will become more pronounced. Findings indicate that *P. neglectus*-resistant lines protect yield and grain quality under high nematode pressures and will provide a powerful new tool for growers dealing with this nematode in Montana.

## Microbiomes of Tobrilid species residing in the Alkaline Lakes of the Western Sandhills of Nebraska

Critchfield, Ricky^1^, P. G. Mullin^2^, T. Harris^2^, K. Powers^2^, T. O. Powers^2^ and D. Porazinska^1^

^1^Dept. Entomology and Nematology, University of Florida, Gainesville, FL 32611

^2^Dept. Plant Pathology, University of Nebraska, Lincoln, NE 68503

### Abstract

The interactions between nematodes and their microbiomes significantly impact nematode biology, ecology, and overall fitness. However, questions remain about how these microbiomes assemble, how they differ among nematode species, and how they affect nematode life. The Alkaline Lakes in the western Nebraska Sandhills provide an ideal system to address these questions due to their variability in alkalinity (pH 8 – 11) and unique nematode communities, primarily composed of several species from the family Tobrilidae. We hypothesized that the host identity and environmental factors would be the major drivers of tobrilid nematode microbiomes. In October 2024, we collected four sediment replicate samples from five lakes and extracted nematodes from three of those lakes using sugar centrifugation and Baermann funnels. Sediment samples and individual hand-picked tobrilid specimens underwent 16S (515F-926R) rRNA metabarcoding via Illumina sequencing to characterize bacterial communities. Nematode identity was determined using Sanger Sequencing with an18S NF1/18Sr2b primer set. Additionally, we characterized the biogeochemistry of the lakes to determine its impact on nematode communities. We employed Generalized Linear Models with post-hoc analysis to assess differences in alpha diversity (e.g., Shannon Index) between microbiome origins (sediments vs. nematodes), lakes (Mallard’s Arm, Island, and Border), and nematode identities (*Semitobrilus sp*., *Tobrilus sp*., *Neotobrilus sp*., and *Brevitobrilus* sp.). Additionally, we used PERMANOVA to examine differences in microbial community compositions. Finally, we used GLM and Pearson’s correlation analysis to understand potential relationships between diversity and biogeochemistry. As expected, the examined lakes differed in biochemistry, with pH, potassium, copper, and sodium levels significantly higher in Border Lake, followed by Island Lake, and Mallard’s Arm Lake. Microbiomes of the lake sediments consistently exhibited greater diversity than those of nematodes. Although alpha diversity varied among the lake sediment microbiomes (highest in Millard’s Arm Lake and lowest in Border Lake), no differences were observed among nematode microbiomes. Similar patterns emerged for microbiome compositions, with lake sediments forming distinct clusters separate from nematodes. Additionally, while each lake supported unique sediment microbiomes, no such distinction was evident among nematode microbiomes. Nematode microbiomes were dominated by Proteobacteria and Cyanobacteria regardless of the lakes, whereas sediment microbiomes displayed richer and more dynamic community patterns. Finally, we observed significant negative relationship between Shannon Diversity of both sediments and nematodes with pH, potassium, and sodium, and positive relationships with calcium and iron. While the impact of nematode identity on their microbiomes still awaits examination, our data suggests that sediment and nematode microbiomes respond to lake identity differentially. However, the underlying relationships with lake biogeochemistry may be more similar for some variables.

## Efficacy of cyclobutrifluram against the coneflower nematode *Aphelenchoides pseudobesseyi* in chrysanthemum

Crow, William T.^1^ and L. Demesyeux^2^

^1^Entomology and Nematology Dept., University of Florida, Gainesville FL 32611

### Abstract

*Aphelenchoides pseudobesseyi* is a newly described species of foliar nematode that was recently separated from *A. besseyi* based on molecular phylogenetic analysis and slight morphological differences. *Aphelenchoides pseudobesseyi* has since been identified as the most common plant-parasitic *Aphelenchoides* sp. infecting ornamental plants in Florida, particularly asters and ferns. This experiment evaluated the efficacy of cyclobutrifluram against infection of chrysanthemum by *A. pseudobesseyi*. Clycobutrifluram applied as a soil drench either 2 or 4 weeks before inoculation with *A. pseudobesseyi* reduced infection compared to inoculated controls and the highest rate reduced infection to near zero. Cyclobutrifluram treatments minimized foliar symptoms and maintained plant quality equal to that of the noninoculated controls. These results indicate that cyclobutrifluram has excellent systemic acropetal movement in chrysanthemum and is efficacious against *A. pseudobesseyi*. This new nematicide from Syngenta Crop Protection shows great promise for foliar nematode management in Florida ornamental production.

## Genetic mechanisms underlying resistance breaking and adaptive evolution in Root-Knot Nematodes

Dai, Dadong and S. Siddique

Dept. Entomology and Nematology, University of California Davis, Davis, CA

### Abstract

Root-knot nematodes (RKNs) are among the most devastating plant-parasitic nematodes, posing a significant threat to global food security. Approximately 100 species of RKNs have been identified, with the most notorious being the polyploid members of the *Meloidogyne incognita* group (MIG; *Meloidogyne incognita*, *M. javanica*, *M. arenaria*). Despite considerable research, significant gaps remain in our understanding of the relationship between polyploidization and adaptive evolution in RKNs. In my previous studies, I have constructed Phased genomes of the MIG, revealing the absence of typical telomere structures in MIG. Instead, these nematodes have evolved alternative lengthening of telomeres (ALT) mechanisms (Dai *et al*., 2023 https://www.nature.com/articles/s41467-023-42700-w.pdf). This ALT mechanism likely contributes to the genomic instability observed in MIG, potentially leading to chromosomal fusion, insertions, deletions, and other genetic variations during evolution. These changes may increase the genetic diversity of MIG, aiding in the expansion of their host range. Recently, an increasing number of MIG resistance-breaking populations have been reported in tomato fields across California. We plan to sequence the genomes of these resistance-breaking populations, assemble phased genomes, analyze telomere diversity, and compare genomic structural changes and subgenome dominance at the subgenomic level. Additionally, we aim to sequence and assemble the genomes of other RKN species, such as *Meloidogyne floridensis* and *Meloidogyne luci*, particularly those that can overcome host resistance in tomato. We will also comparatively analyze the telomere sequences of these RKNs to further elucidate the ALT mechanism. Ultimately, by compare their genetic differences and similarities with MIG, we seek to unravel the molecular mechanisms underlying resistance breaking from a genetic perspective, providing a theoretical basis for field control of resistant breaking nematodes.

## PAPAS: Actionable Science Against Nematodes

Dandurand^1^, Louise-Marie, I. Zasada^2^, C. Gleason^3^, J. Kuhl^4^, W.S. De Jong^5^ and P. Watson^6^

^1^University of Idaho, Moscow ID

^2^USDA-ARS, 3420 NW Orchard Ave, Corvallis, OR

^3^Washington State University, Pullman, WA

^4^Plant Science Department, University of Idaho, Moscow ID

^5^Cornel University, Ithaca, NY

^6^Ag Economics Department, University of Idaho, Moscow ID

### Abstract

Among the many nematodes threatening potato production in the U.S., the potato cyst nematodes (PCN) *Globodera pallida* and *Globodera rostochiensis*, and the root knot nematodes (RKN) *Meloidogyne chitwoodi* and *Meloidogyne hapla,* continue to pose serious threats to productivity. RKN can infect tubers and cause cosmetic damage that reduces potato market value, whereas PCN are quarantined pests in the U.S. and, if left uncontrolled, can cause 80% yield loss. Since there are few or no potato varieties resistant to PCN or RKN, growers must rely on nematicides as the most effective means for control. The goal of the NIFA-SCRI coordinated agricultural project ‘Systems approach to controlling nematodes in U.S. potato production’ is to develop a systems approach to control plant-parasitic nematodes that threaten the potato industry. This group of scientists, educators, and industry is also known as PAPAS: Actionable Science Against Nematodes. Our first objective is to develop decision support tools to assist farmers dealing with nematode infestations. This includes modeling of nematode infestations and the impact of management practice on nematode population densities and the development of an AI platform to synthesize potato/nematode information. Second, we will continue to support and pursue breeding efforts to develop varieties with resistance to important plant-parasitic nematodes of potato including *M. chitwoodi* and *G. pallida*. Our third objective is to leverage the nematode suppressive potential of the trap crop *Solanum sisymbriifolium* for the development of novel nematicides. How this plant suppresses PCN and RKN is being evaluated and the chemistry behind this suppression is being explored. Finally, an active effort is underway to increase knowledge about plant-parasitic nematodes by potato growers. Factsheets, videos, and general information on nematodes can be found at potatonematodes.org.

## Biological and chemical nematicides: not either or both, but differently

Desaeger, Johan^1^ and R. Sikora^2^

^1^University of Florida, Dept. of Entomology and Nematology, Gulf Coast Research and Education Center, Wimauma, FL, 33598

^2^University of Bonn, Institute for Crop Science & Resource Conservation, Bonn, Germany

### Abstract

Biological nematicides are more attractive than chemicals to the public and interest among growers is increasing. However, field efficacy and consistency of biologicals tend to be more variable and site-specific as compared to chemical nematicides. For growers to adopt more biological products, in most cases they will have to be combined with chemicals. Nematicides can be combined in different ways, either sequentially as part of a program (rotational) or by mixing them together (cocktails). Combinations or cocktails of pest and disease control products have often shown to be more effective at killing pests and diseases than each of the products used separately (e.g. chemotherapy cocktails in cancer treatments, antihelmintic treatments in animals and humans). The use of biological nematicides in cocktails or combinations is relatively unknown, but their diversity and different modes of action offer significant opportunities to supplement and improve nematode management programs. One of the most common applications is the use of biologicals to extend nematode control following a chemical nematicide application once the chemical has biodegraded. With the recent emergence of safer and more selective chemical nematicides, this could provide new opportunities for integration with biologicals. For instance, new SDHI nematicides that also have fungicidal activity, could potentially help reduce fungal antagonists to biological nematode control agents and promote their activity. On the other hand, they could very well also harm the actual biocontrol agent. Another interesting research area is nematode-suppressive soils and whether it is possible to over time obtain some type of acquired suppressiveness following repeated applications of biological agents. One of the most popular uses of biologicals is as seed treatments, an area which offers many opportunities for combinations with chemicals to improve their efficacy, either by repelling nematodes from the germinating root system or boosting the young plants innate immunity. In some cases, it may also be possible to leave out the chemical component and just combine different biological agents to improve nematode control. With the range of nematode control products continuing to grow, so will the opportunities for synergy. We will discuss these and other questions with the audience as well as give some results from recent research into biological/chemical nematicide combinations.

## Entomopathogenic nematode genomes & secreted effectors

Adler R. Dillman^1^, A. Baniya^1^, A. K. Lima^1^ and E. M. Schwarz^2^

^1^Dept. Nematology, University of California, Riverside, Riverside, CA 92521

^2^Dept. Molecular Biology and Genetics, Cornell University, Ithaca, NY 14853

### Abstract

Nematodes have been a focus of genomics since the beginning of large-scale sequencing initiatives in the early 1990s. The use of genome sequences and postgenomic tools has had a significant impact on different fields of biology. Several entomopathogenic nematode (EPN) genomes have been sequenced, with many more on the way. Genomes of EPNs are invaluable to understanding the molecular mechanisms of parasitism, symbiosis, and host-parasite interactions. We discuss the new chromosome-level assembly of the *Steinernema hermaphroditum* genome and how this genome has been used in comparative analyses. Nematode parasites release excretory/secretory (ES) products to manipulate host biology. The complex mixture of nematode ES usually contains hundreds of proteins. Although nematode ES has been shown to have immunosuppressive effects on various hosts, our understanding of the effects of individual proteins remains limited. We present the results of studying Shk-domain-containing proteins from *Steinernema carpocapsae* ES and how genomic tools help facilitate this work. Our data suggest that EPNs are a powerful model for genome evolution and understanding parasitic nematode ES.

## Virulence genetics of soybean cyst nematode

Docherty, Lauren^1^, V. Ramasubramanian^1^, C. Hirsch^2^, A. Lorenz^1^ and S. Chen^2,3^

^1^Department of Agronomy and Plant Genetics, University of Minnesota, St. Paul, MN 55108

^2^Department of Plant Pathology, University of Minnesota, St. Paul, MN 55108

^3^Southern Research and Outreach Center, University of Minnesota, Waseca, MN 56093

### Abstract

Soybean cyst nematode (SCN) (*Heterodera glycines*) is a major pest affecting soybean production. SCN is primarily controlled by growing resistant cultivars. However, reliance on a single source of resistance has led to SCN populations with the ability to overcome resistance. Surveys throughout the United States have shown that most SCN populations are virulent on current elite soybean cultivars. While significant work has been done to identify resistance loci in soybean, relatively little is known about SCN genes controlling virulence to different sources of host plant resistance. SCN populations are very heterogeneous and individuals are microscopic, making it difficult to extract sufficient DNA for genomic analysis. To overcome these challenges, 178 inbred lines of SCN were developed for this research. These nematodes were originally collected from field samples and inbred using single-cyst descent for at least 10 generations. The genome of each line was sequenced to an average depth of 40×, aligned to the reference genome, and variants were identified. Virulence phenotypes were also measured for each line using a modified HG-type test. The SCN lines were inoculated on six soybean indicator lines with diverse sources of soybean resistance and one susceptible line. A female index was calculated and used as the phenotype in a genome-wide association study (GWAS). In addition to the GWAS, population structure and rate of linkage disequilibrium decay were evaluated on these lines. The results of this project could be used to develop genetic tests for virulence in SCN populations, allowing for more efficient tracking of virulence shifts across landscapes. This project can also inform breeding efforts for more durable soybean resistance.

## Soil food web survey supports specificity of EPN-*Paenibacillus* sp. Association and identifies potential bacterial antagonists of root weevils in a florida citrus orchard

Dritsoulas, Alexandros^1^, H. egmi^2^, S. Kamali^2^, L. L. Stelinski^2^, L. iepenbrock^2^ and L. Duncan^2^

^1^Agricultural University of Athens, Iera Odos 75, 11855 Athens, Greece

^2^University of Florida, IFAS, Citrus Research and Education Center, 700 Experiment Station Road, Lake Alfred, FL 33850 USA

### Abstract

Diaprepes root weevil (DRW, *Diaprepes abbreviatus*) is a major economic pest of citrus trees in Florida and the Caribbean basin. Biological control by native entomopathogenic nematodes (EPN) has been proposed as a driver of DRW abundance across Florida’s ecoregions. Weevils also typically occupy specific locations within orchards for unknown reasons. To identify potential causes of local patterns of weevil abundance and tree condition, we measured relationships between DRW and edaphic properties (biotic and abiotic) within a central Florida orchard. Adult DRW were trapped and monitored weekly for two years in 94 plots arranged in a grid pattern within a 2.5 ha area. One year after monitoring began, soil in each plot was sampled, and DNA extracted from organisms was recovered by sieving-sucrose centrifugation. Soil subsamples were processed for physicochemical properties and DNA was subjected to metabarcoding (Illumina NovaSeq) for three gene regions (ITS2 rDNA, 16S rDNA, and COI mtDNA). Species-specific qPCR primer-probe sets were also used to measure *Steinernema diaprepesi* and *Heterorhabditis indica.* Here we focus on a restricted set of 124 amplicon sequence variants (ASV, comprising 55 identified *Paenibacillus* species) because of the known entomopathogens in this group and the two species described as ectoparasites of EPNs. Soil pH was strongly associated with *Paenibacillus* ASVs (*P* < 0.001). Fourteen bacterial ASVs were dissociated with DRW (*P* < 0.05), whereas none were positively associated with the weevil according to Spatial Analysis by Distance Indices (SADIE). Several *Paenibacillus* species, elevation, coarse sand particles, and combined ASVs of all identified nematophagous fungi (but no EPN) were significant variables explaining 36% of DRW and tree condition variability in a redundancy analysis. Among 124 *Paenibacillus* ASVs, only *Paenibacillus* sp. JF317562, an ectoparasite of *S. diaprepesi*, was correlated (*P* ≤ 0.05) with that nematode. *Paenibacillus* sp. JF317562 was also correlated with *Fergusobia* sp. AY589425, but with none of the 64 other identified insect species, suggesting an affinity of the bacterium for insect-associated nematode species.

## Harnessing prime editing for enhanced nematode resistance in rice: a sustainable approach to global food security

Dutta, Tushar K.^1,2^ and S. Siddique^1^

^1^Dept. Entomology and Nematology, University of California Davis, Davis CA 95616

^2^Division of Nematology, ICAR-Indian Agricultural Research Institute, New Delhi 110012, India

### Abstract

The rice root-knot nematode, *Meloidogyne graminicola,* causes considerable yield decline in rice crops, particularly in southeast Asia. Climate change has prompted a major shift in rice farming from traditional submerged methods to aerobic systems. Notably, *M. graminicola* has become equally damaging in both upland and direct-seeded rice. Recently, a nucleotide-binding leucine-rich repeat (LRR) receptor type *R* gene, *MG1,* was identified in *Oryza sativa japonica* cv. Zhonghua 11, which confers resistance to the *M. graminicola*. However, cloning an *R* gene in a cultivated species through molecular breeding is time-consuming. As an alternative, the CRISPR/Cas9 system has been used to improve nematode resistance by targeting and knocking out the susceptibility (*S*) genes, which facilitates nematode parasitism. Although *S*-gene mediated resistance is recessively inherited, and thought to be durable, many of these genes are involved in essential host functions, implying a potential fitness cost to the host when mutations are induced. In this context, prime editing, which induces specific point mutations without compromising host fitness, presents a promising alternative. We propose a prime editing model that can modify susceptible alleles of *MG1* gene in *Oryza sativa japonica* cv. Nipponbare to a resistant form. Our research uses cutting-edge gene editing technologies to enhance crop resistance against nematodes, thereby supporting global agricultural productivity and food security.

## Reklemel™ active (fluazaindolizine, Salibro™ nematicide), an effective tool to manage *Meloidogyne enterolobii*

Dyer, David^1^, L. Morales^2^, C. Guarnieri^3^, S. Tewari^4^ and T. Thoden^5^

^1^Corteva Agriscience, Myakka City, FL 34251

^2^Corteva Agriscience, Jalisco 45645, Mexico

^3^Corteva Agriscience, Mogi Mirim, SP, Brazil

^4^Corteva Agriscience, Indianapolis, IN 46268

^5^Biogen, 81677 Münche, Germany

### Abstract

Reklemel™ active (fluazaindolizine, Salibro™ nematicide) is a novel, non-fumigant, chemical nematicide recently launched by Corteva Agriscience. It has a novel and unique mode of action and has demonstrated selective and effective control of many plant-parasitic nematodes, especially a wide range of key root-knot nematode species (*Meloidogyne* spp.). The Guava root-knot nematode (*M. enterolobii*) is an emerging tropical and subtropical pest that has been recently introduced and is a growing concern in multiple countries including the United States. This species of *Meloidogyne* is an aggressive plant parasite that infects a wide range of agriculturally important crops including root-knot resistant cultivars of many of these crops. This talk will summarize the results of recent lab and field-based studies conducted with *M. enterolobii* and will highlight the activity of Reklemel on this nematode.

## Designing and printing 3D models of nematodes: resources for nematode systematic workshop

Eisenback, J. D.

School of Plant and Environmental Science, Virginia Tech, Blacksburg, VA 24061

### Abstract

Three-dimensional models of nematodes can be extremely valuable tools for teaching nematode morphology and taxonomy. Production of these models has two major components: 1) Designing the original model, and 2) Printing the model. Unfortunately, the market for models of nematodes and the structures that make them up is virtually nonexistent; therefore, the utilization of 3D models of nematodes depends on individuals to originate objects to be printed. Models for 3D printing can be made using one of several software programs such as Sketchup, Maya, Blender, Adobe Dimension, Cinema 4d, 3ds Max Design, Recap, Rhino, ZBrush, and numerous others. The learning curve is steep, but the results can be very life-like and useful for teaching nematology. Another way to develop a 3D print model is the old-fashioned way with clay or some other moldable material. After a model is formed, it is photographed from all directions and a 3D scanning software such as Polycam 3D Scanner, 3D Scanner App, Pixel 3D, 3D Scanner – Sapling, etc., and an iPad or iPhone is used to generate a 3D object. With a 3D printer in hand, this model can be printed with a variety of 3D printers. Several options are available for printing 3D model prints, but the most suitable printers for nematode models are either filament or stereolithography. Each of these has its own strengths, weaknesses, and pitfalls that will be discussed during this workshop.

## Field performance of forty-four maturity group IV and V soybean cultivars in a southern root-knot nematode-infested field

Emerson, Michael, B. Baker, K. Hurd and T. R. Faske

University of Arkansas System, Dept. of Entomology and Plant Pathology, Lonoke, AR 72086

### Abstract

Forty-four soybean cultivars marketed as resistant to the southern root-knot nematode (*Meloidogyne incognita*) were evaluated in three on-farm experiments in 2023 in Pulaski County, Arkansas. The nematode damage threshold was severe across all experiments at harvest with an average population density of 916 second-stage juveniles/100 cm^3^ of soil. Host susceptibility was based on the percent of root system galled at the R5–R6 growth stage. Cultivars were considered very resistant if the percentage of root system galled was between 0.0 to 1.0%, resistant 1.1 to 4.0%, and moderately resistant 4.1 to 9.0%. Cultivars that were at least moderately resistant consisted of 17% of the maturity group IV and 61% of the maturity group V entries. There was a greater grain yield average for maturity the group IV cultivars that were at least moderate resistant (69 bu/ac) compared to the other cultivars (37 bu/ac). A similar significant difference was observed in the maturity group V cultivars between those classified as at least moderate resistant (58 bu/ac) compared to the other cultivars (43 bu/ac). These data illustrate the limited availability of *M. incognita-*resistant cultivars in the maturity group IV compared to maturity group V cultivars adapted for production in Arkansas and the Mid-Southern United States.

## Field efficacy of seed- and soil-applied nematicides in hybrid corn

Emerson, Michael, B. Baker, K. Brown and T. R. Faske

University of Arkansas System Division of Agriculture, Department of Entomology and Plant Pathology, Lonoke Extension Center, Lonoke, AR 72086

### Abstract

Four seed-applied and four soil-applied nematicides were evaluated in 2021 and 2022 for nematode control and grain yield protection in two on-farm experiments in Jackson County, Arkansas. Fall densities of *Paratrichodorus minor* (60/100 cm^3^ soil) in 2021 were slightly above the damage threshold (40/100 cm^3^ soil) for corn production in Arkansas but low in 2022 (<10/100 cm^3^ soil), while densities of *Meloidogyne incognita* and *Pratylenchus* spp. were low (<100/100 cm^3^ soil) in both experiments. In 2021, terbufos (Counter 20G) was the only nematicide that suppressed mid-season densities of *P. minor* and *Pratylenchus* spp. compared to the non-treated control. Whereas, in 2022, there was no suppression of nematode densities by a nematicide. Nematicides had no impact on grain yield protection in either experiment. These data support the inconsistency of nematode suppression and yield protection by nematicides when multiple species of corn nematodes are present at low densities.

## Preparation and imaging of monhysterid nematodes with a scanning electron microscope

Farrer, Solinus^1^ and B. Adams^1,2^

^1^Dept. Biology, Brigham Young University, Provo, UT 84602

^2^Monte L. Bean Life Science Museum, Provo, UT 84602

### Abstract

The use of scanning electron microscopy (SEM) improves the study and observation of nematodes by revealing morphological features of the integument, posterior, and anterior. While advantageous, SEM imaging of nematodes is difficult due to the fragile and delicate nature of biological samples. The technique demands proper sample preparation in order to maintain the structure of the soft tissue and protect it from charging caused by the electron beam. Samples of monhysterid nematodes were obtained from the Great Salt Lake and killed using glutaraldehyde as the primary fixative. They were then washed in a cacodylate buffer and put in osmium tetroxide as a secondary fixative. After fixation, samples were dehydrated in an ethanol series and then critical point dried, which eliminates the shrinking caused by surface tension and maintains the structure of the nematodes. After mounting dried samples on SEM sample mounts using carbon tape, a AuPd coating was applied to protect the samples from the electron beam by making them conductive. We found that with these sample preparation techniques we were able to obtain the highest quality SEM images of nematodes.

## Nematode Faunal Analysis: foundational principles and future evolution

Ferris, Howard

Department of Entomology and Nematology, University of California Davis

### Abstract

Nematode Faunal Analysis provides a conceptual framework for an indicator system of the condition of the soil food web and of the soil environment. The organisms of the soil food web perform functions and services that are essential for life on Earth. Synoptic procedures are critically needed for evaluating the condition of the soil ecosystem and, if necessary, providing a basis for management criteria. Soil nematodes are useful indicator taxa because of their ubiquity, the diversity of their habits and functions, and their intermediate persistence and turnover rates. Nematodes of different structural (c-p) groups separate into enrichment opportunists, basal fauna present in most soils, and indicators of food web structure and complexity. They depend upon the carbon and energy provided by combinations of plant sources and the microbiome engine of the soil ecosystem. Graphical and statistical representation of proportions and biomass of the nematode groups can indicate soil health and the magnitude of carbon and energy fluxes in the system. Interpretation of indicator assessments requires consideration of the nature of the soil ecosystem and of the successional and auto-regenerative processes that follow disturbances to, or changes in, the system. Within the nematode assemblage, structural guilds (c-p groups) and functional guilds (feeding categories within c-p groups) differ in sensitivity to stress; following disturbance, they undergo structural and functional succession but at different rates. Reliable optical, molecular, or AI-based identification of resident nematode taxa, and their justifiable assignment to functional guilds, is important. The faunal analysis system is, in essence, a conceptual model and, as such, must be subjected to continued verification and validation. The c-p groupings and functional guilds are currently resolved at the family level. Clearly, there are data gaps and research challenges centered on improving resolution of knowledge of the fundamental biology, behavior, feeding habits and life-course dynamics of taxa, at least at the genus level, for assignment of most non-parasitic taxa to functional and structural guilds. Although the assignment of taxa to the c-p 1 and 2 groups is fairly intuitive, the criteria that separate c-p 3, 4, and 5 groups, which include body size, longevity, and sensitivity to stress, are less well defined. More decisive definitions will emerge from a detailed study of the biology of those taxa. The Dorylaimda, in particular, is a huge group of nematode families, genera, and species that span the c-p 3-5 groups and which requires more detailed study. Finally, considering the wide-scale use of nematode-based indicators, some processes for agreeing on changes in the assignment and documentation of taxa to different life histories and feeding categories will be important.

## Effectiveness of nematicides for suppression of plant-parasitic nematodes in a Young Okanagan Valley, British Columbia Vineyard

Forge, Tom^1^, P. Munro^1^ and M. Thurston^2^

^1^Agriculture and Agri-Food Canada, Summerland Research and Development Centre, Summerland, British Columbia, V0H 1Z0, Canada

^2^Pearl Agricultural Consulting, Inc., Lake Country, BC, V4V 1P9, Canada

### Abstract

Winter cold injury, trunk disease complexes and general declines in productivity are driving an increase in replanting of vineyards in the Okanagan Valley of British Columbia (BC). Most vineyards are infested with plant-parasitic nematodes, but restrictions on fumigation have left few options for managing nematodes prior to replanting. The utilization of post-plant nematicides on replanted vines is an alternative approach that deserves examination. In two trials initiated in 2021 and 2022, respectively, we assessed the efficacy of oxamyl, fluopyram, fluazaindolizine at variable rates, and combinations of oxamyl and fluazaindolizine, for control of *Mesocriconema xenoplax*, *Xiphinema bricolensis*, *Meloidogyne hapla* and *Paratrichodorus minor* in a replanted block of Merlot on 3309C rootstock in loamy sand soil. In each trial, eight treatment combinations were applied to five replicate 7 m long row-plots in a randomized complete block experimental design. Treatments were re-applied in each of the two and one years after initiation of the 2021 and 2022 trials, respectively. Both trials were terminated at the end of 2023. Soil samples were taken, and nematode population densities were measured at seven and five times through the three and two growing seasons after initiation of the 2021 and 2022 trials, respectively. Data were analyzed using repeated measures ANOVA models. Analyses indicated significant main-factor effects of treatment, but not treatment × date interaction, for *M. xenoplax* and *X. bricolensis* in the 2021 trial. No other nematode group was affected by treatment or treatment × date interaction in the 2021 trial, and neither the main-factor effect of treatment nor the treatment × date interaction was significant for any of the nematode groups in the 2022 trial. There were no significant effects of treatment or treatment × sample date interaction on the abundance of free-living nematodes in either trial. In the 2021 trial, only the combined application of 1120 g ha^−1^ fluazaindolizine + 2240 g ha^−1^ oxamyl resulted in main-factor mean *M. xenoplax* population densities lower than in the untreated control. In contrast, mean population densities of *X. bricolensis* were suppressed relative to the untreated control in five of the treatments: fluazaindolizine at 1120 and 2240 g ha^−1^, 2240 g ha^−1^ oxamyl, and combined applications of 560 g ha^−1^ fluazaindolizine+oxamyl and 1120 g ha^−1^ fluazaindolizine+oxamyl. Population densities of *M. hapla* remained low in all treatments throughout the trial, likely as a result of rootstock resistance to *M. hapla*. There were no significant effects of treatments on vine growth parameters in either trial, perhaps due to lack of consistent control of *M. xenoplax* which was the dominant plant-parasitic nematode at the sites. In summary, our results indicate under BC vineyard conditions, multi-year applications of fluazaindolizine and oxamyl, alone and combined can suppress *X. bricolensis*.

## Utility or futility of alternative rootstocks and soil amendments for management of the root-lesion nematode, *Pratylenchus penetrans* in replanted apple orchards

Forge, Tom^1^, P. Munro^1^, S. Ali^2^, V. Lévesque^2^ and K. Fuller^2^

^1^Agriculture and Agri-Food Canada, Summerland Research and Development Centre, Summerland, British Columbia

^2^Agriculture and Agri-Food Canada, Kentville Research and Development Centre, Kentville, Nova Scotia

### Abstract

The root-lesion nematode, *Pratylenchus penetrans,* is a cosmopolitan pest of apple trees. Most high-density apple orchards in Canada have been planted on dwarfing rootstocks, most of which are known to be susceptible to *P. penetrans* and the broader apple replant disease (ARD) complex. Soil fumigation has been the primary means of reducing *P. penetrans* populations and ARD prior to replanting apple orchards. However, fumigants pose significant risks to human health and the environment and do not provide long-term control. Several Geneva-series dwarfing rootstocks are considered to be tolerant to ARD, but little is known of their host status for *P. penetrans*, specifically. The objective of this research was to determine the effects of organic amendment-based soil treatments on *P. penetrans* infestation of four different rootstocks: M.9, M.26, G.41 and G.935. Field experiments were conducted in the Okanagan Valley of British Columbia (BC) and the Annapolis Valley of Nova Scotia (NS). Pre-plant soil treatments in BC were: 1) municipal compost amendment (COM), incorporated into tree-row soil at 50 dry Mg ha^−1^; 2) anaerobic soil disinfestation (ASD), with 20 dry Mg ha^−1^ alfalfa hay carbon source; 3) dazomet fumigation (FUM); and 4) untreated (UT) control. Soil treatments in NS were: 1) ASD 20 dry Mg ha^−1^ with grass hay carbon source; 2) mustard seed meal (MSM); 3) oxamyl nematicide (ON); 4) FUM; and 5) UT control. All chemical treatments were applied at label rates. Both experiments were set up using split-plot experimental designs with rootstocks planted as sub-plots within soil treatment whole-plots. Population densities of *P. penetrans* in soil were assessed multiple times through the first two growing seasons, and population densities in roots were assessed twice in the second growing season. There was no rootstock × soil treatment interaction or rootstock main-factor effects on *P. penetrans* g^−1^ root at either site, indicating that the four rootstocks were similarly susceptible to *P. penetrans* across a wide range of soil conditions. There were significant main-factor effects of soil treatment at both sites. In BC the COM and ASD treatments resulted in lower *P. penetrans* g^−1^ root than in the UT control and were not different from the FUM treatment. In NS, the ASD and MSM treatments resulted in lower *P. penetrans* g^−1^ root than the UT control and neither differed from the ON or FUM treatments. These results indicate that preplant treatments with compost, anaerobic soil disinfestation or mustard seed meal can reduce infection of apple roots by *P. penetrans* and improve early growth of apple trees in replant sites.

## Microplot design and construction for plant parasitic nematode research

Forsberg, Lance, R. Yazdani and M. Quintanilla

Department of Entomology, Michigan State University, East Lansing, MI, 48824

### Abstract

Microplots were recognized as a valuable tool for conducting precision agricultural research due to their ability to provide better controlled and replicated field conditions for studying various agronomic practices and crop responses. This abstract is an overview for the establishment and design of a 600 cell microplot system at Michigan State University’s Entomology Farm. The microplot field had a 30-meter by 93.5-meter perimeter, which is surrounded by a 2.4-meter-high woven wire fence with two 6.1 meters gate openings for vehicle and farm equipment access. A 14-meter by 79-meter road was constructed in the middle, creating two sections with 300 microplots on each side. The road is 25 cm below surface level with a 1% grade to help with drainage from weather events. Individual microplots were constructed using a 38 cm inside diameter polypropylene storm pipe cut into 51-centimeter-long pieces, placed in 61 cm deep holes, elevated 25 cm above ground level, and filled with 0.08 cubic meters of 2NS sand. Hole excavation was carried out using a Caterpillar 259D3 skid steer with a 46 cm auger attachment. The Hp polypropylene pipe was chosen for its resistance to Ph ranges of 1.5 to 14, chemicals, and UV exposure for more than 10 years. The microplot layout was established in a 6 row by 10 row design using a stringline at a 90-degree angle to determine block pattern length and width. The pattern was spray painted on the ground, pipes removed, and holes drilled 91 cm apart in a 6x10 block pattern, totaling 10 blocks and 600 individual microplots. Spacing between 60 cell 6x10 pattern is 3.66 meters. Extra soil from drilled holes was evenly spread, and a 20-inch 212 cc Legend roto tiller was used for breaking up the soil. The ground was covered with 315w woven geotextile cloth, holes were tightly cut by hand around each microplot to create a tight skirting to help hold individual tubes at an exact 25.4 cm height until the field soil settled. The ground surface was covered with 5 cm of #9 limestone to aid drainage, minimize cross contamination, and control weeds. Field soils from commercial farms around Michigan were selected based on the presence of specific nematode species. Representative soil samples were analyzed molecularly for nematode identification and population counts. Each microplot received 0.029 cubic meters of field soil by hand, with each 6x10 block representing a single plant parasitic nematode species for research. Nematode populations were verified for each individual microplot before conducting any research. In conclusion, this 600-cell microplot facility serves as a valuable tool for plant-parasitic nematode research, enabling controlled study and management of these agricultural pests.

## Impacts of elevated soil temperatures on nematode reproduction and virulence

GC, Sagar^1^, C. Khanal^1^ and P. anakar^2^

^1^Dept. Plant and Environmental Sciences, Clemson University, Clemson, SC, USA

^2^CCS Haryana Agricultural University, Department of Nematology, Hisar, Haryana, India

### Abstract

Climate change is one of the major issues of the millennium, and its adverse impacts on agriculture can lead to global food insecurity. Temperature is the major factor that influences climate change; however, relationships between warmer soil temperature and nematode biology is not fully understood. Studies were conducted to understand the effects of elevated soil temperatures on the reproduction and virulence of reniform nematodes (*Rotylenchulus reniformis*). Four different soil temperatures, 26°C (control), 32°C, 34°C, and 36°C, were established using a commercial heat mat in a growth room environment. Four-week-old tomato seedlings (cv. Rutgers) were transplanted in steam-sterilized 3:1 clay and sand soil in six-inch plastic pots, followed by inoculation with 10,000 *R. reniformis* eggs on the day of transplanting. The reproduction of *R. reniformis* was significantly impacted by the soil temperature and ranged from 13,727 to 29,919 eggs/g root. The nematode reproduction was increased at 32°C and 34°C; however, the increase was significant only at the latter temperature relative to the control. The reproduction was decreased at 36°C and was statistically similar to that at the control. The root dry biomass ranged from 0.08 to 0.17 g, with the lowest and the greatest biomass being at 36 and 26°C, respectively. Relative to the control, the root biomass was significantly lowered at 32°C and 36°C, however, it was not significantly different at 34°C. Results from the current study suggest that nematode reproduction increases with an increase in temperature up to 34°C and again reduces at 36°C probably because the soil temperature at the upper limit is not suitable for the growth and development of both nematodes and plants. Additionally, the virulence of the nematode seems to increase with increased temperature although increments may not always be statistically significant. Results from the current study suggest that an increase in soil temperature will lead to increased crop damage due to increased nematode virulence, implying the need for the search of measures to limit the adverse impact of climate change on crops.

## Mitochondrial metagenomics for high throughput nematode identification – what is it? How good is it? How to use it?

Gendron, Eli, J. Sevigny^1^, W. K. Thomas^1^ and D. L. Porazinska^2^

^1^Hubbard Center for Genome Studies, Durham, NH 03824

^2^Department of Entomology and Nematology, University of Florida, FL 32611

### Abstract

The most common methods of nematode identification are based on morphology and/or the use of well-established single specimen DNA barcodes. However, both morphology and DNA barcoding methods are laborious, require extensive expertise, and have limited throughput. Metabarcoding techniques offer solutions to some of these shortcomings by significantly increasing throughput. However, they generally lack the ability to recover the entire nematode diversity due to limited primer coverage and/or taxonomic resolution stemming from limited amplicon sizes. We have recently identified mitochondrial metagenomics (mtMG) as a potential addition to the suite of techniques available for nematode identification. By bypassing the need for morphological inspection, taxon-specific primers, or limited resolution of gene markers, while still supporting high throughput processing, mtMG has the potential to allow for more comprehensive and accurate identification of nematode communities. We have tested this approach using nematode mock communities as well as controlled environmental communities. In addition, we developed protocols for sample processing and data analysis along with the construction of a curated mitochondrial reference sequence database to facilitate the analysis. In this 2-hr long workshop, we will provide: 1) a detailed overview of the mtMG process along with its pros and cons, 2) a demonstration of data analysis and how to incorporate its use in research and field surveys, 3) an access to all available protocols and databases.

## Influence of ecological and edaphic factors on plant-parasitic nematodes associated with tropical fruit trees in South Florida

Gitonga, Denis^1^, D. Carrillo^2^ and A. Hajihassani^1^

^1^Dept. of Entomology and Nematology, University of Florida Fort Lauderdale Research and Education Center, Davie, FL 33314

^2^Dept. of Entomology and Nematology, University of Florida, Tropical Research and Education Center, Homestead, FL 33031

### Abstract

Plant-parasitic nematodes (PPN) have been reported to cause damage to several species of tropical fruits in Florida, one of the leading tropical fruit producers in the U.S. Most tropical fruit cultivation in South Florida occurs in Calcareous soils. However, the influence of PPNs on fruit tree growth performance has been poorly investigated. From July 2022 to September 2023, 65 fruit tree orchards (18 avocados, 17 guavas, 9 mameys, 5 longans, 4 starfruits, 3 mangoes, three lychees, 2 dragon fruits, 2 passion fruit, 1 papaya, and 1 banana) were randomly selected for sampling in Homestead, Florida to determine the incidence and distribution of PPNs. Principal Component Analysis (PCA) was used to examine the relationships between PPNs and edaphic factors, including soil texture, temperature, organic matter, pH, and electrical conductivity (EC). The frequency of occurrence (%), mean relative abundance, and maximum relative abundance (per 100 cm^3^ of soil) of the 11 different PPN genera detected in 11 different fruit tree species were as follows: *Rotylenchulus* spp. (incidence, 73.8%; mean, 67.4; maximum, 900), *Aphelenchus* spp. (58.5%; 5.8; 60), *Mesocriconema* spp. (49.2%; 33.4; 490), *Helicotylenchus* spp. (46.2%; 19.6; 185), *Meloidogyne* spp. (30.8%; 11.9; 260), *Pratylenchus* spp. (21.5%; 1.3; 20), *Xiphinema* spp. (18.4%; 1.0; 16), *Hoplolaimus* spp. (13.8%; 2.9; 72), *Tylenchorrhynchus* spp. (12.3%; 3.3; 102), *Trichodorus* spp. (12.3%; 1.0; 28), and *Ditylenchus* spp. (4.6%; 0.5; 26). PCA indicated that fruit tree type (host), soil texture, and organic matter significantly influenced the distribution and population density of PPNs. Reniform, lance, and root-lesion nematode abundance and distribution were more associated with avocado; root-knot (RKN) and spiral nematodes were associated with guava and dragon fruits, while ring and spiral nematodes were more associated with lychees, longans, and mangoes. Multiple regression models indicated that the presence of RKNs was influenced by an increment in sand content and the age of the orchards. Additionally, the fruit tree variety and organic matter influenced the abundance of reniform nematode in avocados. Results indicate that in addition to the direct influence of the host plant, soil physicochemical parameters are critical for PPN dispersion and abundance. These findings will help to increase knowledge of the prevalence of economically significant nematodes in Florida fruit tree orchards, enabling the establishment and implementation of appropriate control measures.

## Screening for new sources of resistance against plant-parasitic nematodes in wild tomato

Godinez-Vidal, Damaris and S. C. Groen

Dept. of Nematology, Riverside, University of California, Riverside, CA 92521

### Abstract

Plant-parasitic nematodes are a devastating threat to many vegetables. For crops such as tomato, nematodes lead to yield losses of up to 20%. Important nematode parasites include the root-knot nematodes (RKNs), which attack over 4,000 plant species and generate billions of dollars in crop losses annually worldwide. In addition, farmers are now finding RKNs in their supposedly resistant tomato crops in increasing numbers. The *Mi-1* gene, discovered in wild tomatoes, has been the only source of resistance against the RKNs *Meloidogyne incognita*, *M. javanica*, and *M. arenaria*. However, this has put RKNs under high selection pressure to overcome *Mi-1*-mediated resistance. To search for new sources of resistance, we examined forty accessions of wild and weedy tomato plants. These plants naturally evolved in areas with varying levels of RKN activity, so we expect some will have developed effective defenses against RKNs. The tomato accessions were grown under greenhouse conditions and exposed to juveniles of the second stage of *M. incognita*. We obtained data on plant growth as well as fitness and galling numbers of *M. incognita* during three weeks of infection. We find four highly susceptible accessions, seven tolerant accessions, and nine accessions with low gall numbers, suggesting a great potential as sources of resistance against *M. incognita* infection. In susceptible accessions, we observed typical yellowing, stunting, and wilting symptoms. Crop damage results from a complex interaction in which RKNs start injecting effector proteins with their saliva into host cells to dampen the plant’s defense response. To understand this process better, we performed genome-wide gene expression profiling on a subset of accessions to link root gene expression to tomato resistance to *M. incognita*. Preliminary analyses showed that expression of two genes was negatively correlated with nematode galling. One is *DHRS4*, which encodes dehydrogenase/reductase SDR family member 4 and is implicated in biosynthesis of calystegines, which are alkaloid secondary metabolites. The other is *MYB60*, which encodes a MYB transcription factor that is known to regulate root growth. Expression of its ortholog in *Arabidopsis thaliana* shows induction of expression early during cyst nematode infections. Our observations and experiments show the tremendous potential of some of the tolerant and resistant accessions and the possibility of finding novel genes that can contribute to RKN resistance.

## Hexavalent chromium in soil affects the behavior of the entomopathogenic nematode *Steinernema feltiae*

Godjo, Anique, D. Mc Donald, L. Ansaldi, S. Boyle, J. Byrne and T. akouli Duarte

Molecular Ecology and Nematode Research Group, enviroCORE, Department of Applied Science, South East Technological University, Kilkenny Road Campus, Carlow, R93 V960, Ireland

### Abstract

Essential information about the effects of a pollutant on the local ecosystem can be obtained by observing how it influences a bioindicator organism. The heavy metal hexavalent chromium (Cr VI^+^) has been reported to be naturally present at concentrations of 5–250 mg/kg in Irish agricultural soils. This compound is very dangerous to human and environmental health. Therefore, the aim of this study was to understand the impact of this potential toxicant on soil biota, by using the entomopathogenic nematode (EPN) *Steinernema feltiae* as a sentinel organism. An Irish EPN isolate (SB 12(1)), was chosen to risk assess soil Cr VI^+^ pollution. With tenfold replication, the attraction of nematodes to insect host (late instar larvae of *Galleria mellonella*) was investigated using varying lengths of PVC tubes (5, 10, 15, 20 cm) loaded with Cr VI^+^ polluted play sand and various doses of Cr VI^+^ (50–300 ppm in increments of 50). The percentage of *G. mellonella* mortality was recorded at the end of the experiment, and death caused by nematodes was ascertained by dissecting dead larvae under a stereoscope. Positive control tubes received nematodes but no Cr VI^+^ and negative control tubes received i) no nematode and no Cr VI^+^ and ii) no nematode but Cr VI^+^. In addition, *S. feltiae* reproduction in *G. mellonella* in the presence of various Cr VI^+^ concentrations (70–100ppm in increments of 5 and 0–300ppm in increment of 50) was studied. Results showed that the distance between the host and the nematode application point, as well as the concentration of Cr VI^+^, had a significant impact on the attraction of *S. feltiae* to the host. Higher insect mortality of 100% and 88.89% were recorded at low Cr VI^+^ concentrations (positive control and 50ppm, respectively) over 5 cm. There was a lack of significant differences in insect mortality between the negative control and the 150–300 ppm Cr VI^+^ levels, suggesting that the threshold of the Cr VI^+^ effect on the attraction of *S. feltiae* to its host lies within that range of metal concentration. Furthermore, the percentage host penetration and reproduction of *S. feltiae* were not significantly affected by the presence of Cr VI^+^ at 70–100ppm. This confirms that the threshold of the Cr VI^+^ effect on the development of *S. feltiae* is above 100 ppm. Data collected from an experiment that studied the effect of Cr VI^+^ on *S. feltiae* reproduction potential at higher metal concentration (up to 300ppm) is currently undergoing analysis. Overall, *S. feltiae* demonstrated excellent potential for assessing the risk of Cr VI^+^ soil contamination and is a suitable sentinel organism that can be used for an internationally accredited protocol.

## Deciphering the vectors: unveiling the local dispersal of *Litylenchus crenatae* ssp. *mccannii* in forest ecosystem

Goraya, Mankanwal^1^, C. Kantor^2^, P. Vieira^3^ and M. Kantor^1^

^1^Plant Pathology & Environmental Microbiology Department, The Pennsylvania State University, University Park, PA 16802

^2^Huck Institutes of the Life Sciences, Pennsylvania State University, University Park, PA 16802

^3^Mycology and Nematology Genetic Diversity and Biology Laboratory, USDA, ARS, Northeast Area, Beltsville, MD 20705

### Abstract

Beech leaf disease (BLD), caused by the North American beech leaf nematode, *Litylenchus crenatae* ssp. *mccannii* (Lcm), is an emerging threat to beech trees, characterized by symptoms such as interveinal banding, thickened leaf texture, and eventual tree mortality. Understanding Lcm dispersal mechanisms is crucial for managing BLD, yet these remain largely unknown, posing a major barrier to its effective management. This study represents a pioneering investigation into the abiotic and biotic vectors potentially contributing to the local transmission of Lcm in natural forest systems. The experiment was set up in a natural forest using four funnel stands, each with three water-filled funnels, placed at variable distances and different radial growth of BLD-infected beech trees. Active Lcm nematodes were recovered from each funnel demonstrating their ability to travel at least 11.74 m from the nearest BLD-infected tree. The findings underscore the role of abiotic vectors, as indicated by a generalized linear model with negative binomial distribution selected as the best fit to predict the influence of weather variables on the transmission of Lcm. The results indicated the positive significance of wind and humidity, with precipitation as a lesser albeit still significant variable in the transmission of Lcm. Furthermore, the current study uncovered the unspecific association of Lcm with other biotic forms under the canopy of infected beech trees, including beech blight aphids, and spiderwebs. The presence of active Lcms in caterpillar frass revealed the ability of Lcm to remain undigested in the gut of the Tussock moth caterpillar, thus having a potential role in the indirect dispersal of BLD inoculum. To our knowledge, this study is the first to document the potential vectors involved in the local dispersal of Lcm in natural forest environments and offers valuable information for developing effective BLD management strategies. The findings represent a significant advancement in understanding the local spread of BLD and highlight the complex interplay of abiotic and biotic factors in disease transmission.

## Movement and fluctuation in root-knot nematode (*Meloidogyne* spp.) Population densities quantified through soil sampling in north Carolina sweetpotato production

Gorny, Adrienne^1^, P. B. Jeffreys^1^, K. Cox^1^, J. Dotray^1^ and R. uckaba^2^

^1^Department of Entomology and Plant Pathology, North Carolina State University, Raleigh, NC 27695

^2^Teleos Ag Solutions, Hamlet, NC 28345

### Abstract

In North Carolina, sweetpotatoes are typically grown on bare-ground, raised beds with a pre-plant fumigant application where root-knot nematode (RKN) pressure is high. To save on cost, these pre-plant fumigants are typically not applied to the whole field (broadcast), but rather applied only to the center of the raised bed (row application). In the case of row application, it is hypothesized that RKN from non-treated areas between the rows (row middles), or regions of reduced fumigant concentration (bed “shoulders”) may re-colonize the planting bed as the season progresses, through either natural nematode movement or cultivation events. To document potential migration and fluctuation in RKN population density between treated bed centers, shoulders, and non-treated row middles, an intensive soil-sampling program was undertaken in the field during the 2023 growing season. Fumigant (Telone II, 1,3-dichloropropene at 8 gal/Ac) was applied within the row to small plots (4 rows wide by 100 ft long). Planting beds (40-inch row spacing) were formed at time of fumigant application. Non-treated plots served as the control and treatments were arranged in a randomized complete block design, with 4 replicate plots per treatment. Three weeks after the fumigant application, sweetpotatoes (cv. ‘Covington’) were planted. Beds were cultivated for weed management following standard practices for sweetpotato. Soil samples were collected from the bed center, row middle, and bed shoulders (measured as approximately halfway between row center and row middle) from both shallow (0–6 inch) and deep (6–12 inches) depths. In each plot, six subsamples were collected from each location × depth combination and bulked to form a composite sample. Samples were collected at planting, and at 2, 4, 6, and 8 weeks post planting. Root-knot nematodes were extracted via a modified Whitehead tray, counted under the microscope, and population densities estimated for the sample. In non-treated plots, higher RKN population densities were observed at the 6- and 8-weeks sampling time in row centers and bed shoulders, at both depths, compared to row middles, with some of the differences being statistically significant at α=0.05 level. Telone II-treated plots had lower population densities at all locations × depths at the 6- and 8-weeks sampling times, when compared to non-treated plots. The soil sampling survey will be repeated and results from this survey will be used to inform fumigation best management practices.

## Assessing cost effectiveness of nematicide rates and chemistries for sting nematode management in Florida potato

Grabau, Zane J.^1^, R. Sandoval-Ruiz^1^ and C. Liu^2^

^1^University of Florida, Entomology and Nematology Department, Gainesville, FL 32611

^2^Mississippi State University, Department of Biochemistry, Molecular Biology, Entomology, and Plant Pathology, Mississippi State, MS 39762

### Abstract

*Belonolaimus longicaudatus* (sting nematode) is a yield-robbing pathogen in Florida potato production. Management mostly relies on fumigant nematicides, with 1,3-dichloropropene (1,3-D) at 66.3 kg active ingredient (a.i.)/ha a common practice. However, fumigation costs have increased, reducing profit margins. This research aims to identify alternative nematicide rates and chemistries to reduce application costs while effectively managing sting nematode and the effect of these treatments on non-target, beneficial, free-living nematodes. A replicated (n=6) small plot field trial was conducted in Northeast Florida in 2023 and is being repeated in 2024. Treatments included fumigant nematicides metam potassium at rates of (i-ii) 125 or 293 kg a.i./ha; and 1,3-D at (iii-v) 33.1, 49.7, or 66.3 kg a.i./ha. Treatments applied in-furrow at planting were the liquid nematicides (vi) oxamyl at 1.12 kg a.i./ha or (vii) fluopyram at 249 g a.i./ha. Finally, a combination of (viii) 1,3-D at 33.1 kg a.i./ha followed by in-furrow oxamyl at 1.12 kg a.i./ha, and (ix) untreated control were tested. In addition to yield and vegetative growth, nematode soil populations were assessed at midseason (6 weeks after planting) and harvest. In 2023, any treatment with 1,3-D or metam potassium fumigation effectively managed sting nematode soil abundances relative to untreated. Applications of only fluopyram or oxamyl did not manage sting nematode soil abundances. Free-living nematodes were not affected by any of the treatments. In 2023, there was evidence of phytotoxicity from metam potassium based on stand counts at 28 days after planting. Fumigation using 1,3-D alone, at 49.7 and 66.3 kg a.i./ha or 1,3-D followed by oxamyl increased income as well as total and marketable yield compared to untreated. No other treatments significantly changed yield or income relative to untreated. From the 2023 trial, 1,3-D fumigation was the most effective treatment for managing sting nematode in potato, but growers may be able to reduce application rates without sacrificing efficacy. Results from the 2024 field trial will help determine consistency of these nematicide treatments.

## Efficacy of resistant cultivars and nematicide application for managing reniform nematode in cotton

Grabau, Zane J.^1^, R. Sandoval-Ruiz^1^ and C. Liu^2^

^1^University of Florida, Entomology and Nematology Department, Gainesville, FL 32611

^2^Mississippi State University, Department of Biochemistry, Molecular Biology, Entomology, and Plant Pathology, Mississippi State, MS 39762

### Abstract

*Rotylenchulus reniformis* (reniform nematode, RN) is a major plant-parasitic nematode on cotton which has typically been managed using nematicide application and crop rotation. Recently, commercial cotton cultivars resistant to RN have been introduced. The objective of this research was to assess the efficacy of resistant cultivars and nematicide application for management of RN. Two replicated small plot field experiments were conducted. The first experiment (N=5), conducted in 2021 and repeated in 2022 and 2023, compared a susceptible (Deltapine 1646 B2XF [DP1646]) and a resistant (Deltapine 2141NR B3XF [DP2141NR]) cotton cultivar. These cultivars were crossed with the in-furrow nematicides (i) aldicarb or (ii) fluopyram; seed coating treatments of (iii) heat-killed *Burkholderia* bacteria, (iv) fluopyram; or (v) no nematicide control. The second experiment (N=6) compared (i) RN-susceptible Phytogen 444WRF (PHY444), (ii) PHY444 with in-furrow fluopyram, and (iii) RN-resistant Phytogen 443W3FE (PHY443) in 2021 and 2022. In both experiments, reniform nematode resistant cotton cultivars were highly effective. In the first experiment, across years, DP2141NR reduced midseason RN abundances from roots by 94% and RN soil abundances at harvest by 67% relative to the susceptible DP1646 when neither cultivar received nematicide. DP2141NR had variable yield return, with a decrease of 12% in 2021, but increases of 56% and 21% in 2022 and 2023, respectively, relative to DP1646, averaged across nematicide treatments. Under DP2141NR, nematicides did not improve management of RN abundances. Under the susceptible DP1646, only aldicarb reduced RN abundances relative to control, reducing midseason RN abundances in roots 84% and harvest soil RN abundances 25% under DP1646. Aldicarb and *Burkholderia* seed treatment increased cotton lint yield 13% and 10% relative to control, averaged across cultivars. In the second experiment, the resistant cultivar PHY443 reduced midseason RN abundances from roots 80% and harvest RN soil abundances 65% relative to the susceptible PHY444 without nematicide. PHY443 also increased yield 42%. Fluopyram application with PHY444 reduced final RN soil abundances 30% but did not affect yield or RN abundances from roots. In summary, DP2141NR and PHY443 resistant cultivars are highly effective options for managing RN in cotton under severe infestation. Aldicarb was the only consistently effective nematicide at managing RN.

## Trailblazers of science, the case of nematodes as model organisms

Grimberg, Bruna Irene

Western SARE and Montana State University, Dept. Cell Biology and Neuroscience, Bozeman, MT 59717

### Abstract

Grounded in evolutionary biology, scientists developed the notion of model organisms implying that a particular organism can serve as a sample of a larger class of organisms that are phylogenetically related to it. As such, model organisms facilitate the understanding of complex biological phenomena based on the presumption of their representational power that allows the extrapolation of data and results beyond the studied organism. Questions arise about the validity of the representativeness of model organisms and the consequences of the inductive idealization and abstraction of this kind of research. Why are model organisms accepted as credible representations of biological phenomena? Are model organisms a viable scientific model for creating knowledge? How do their roles-as representational and interventionist organisms impact science research practices? The presentation will address these questions by analyzing the aspects and impact of two kinds of model organism projections, 1) the representational scope, referring to genetic makeup similarity-based representation, and 2) the target scope referring to phenomena-based representation. Application for science teaching and research will be discussed.

## Confirmation of fluopyram resistance in *Meloidogyne graminis* and *Belonolaimus longicaudatus* populations

Guri, Erica and W. T. Crow

Entomology and Nematology Dept., University of Florida, Gainesville, FL 32611

### Abstract

Grass root-knot (*Meloidogyne graminis*) and sting (*Belonolaimus longicaudatus*) nematodes are two of the most destructive nematodes affecting turfgrass, particularly in warm climates like Florida. Recent reports have indicated a decline in the effectiveness of fluopyram, a commonly used nematicide, used for managing these nematodes. This decline in efficacy raises concerns about the development of resistance, prompting the need for further investigation. The objective of this study was to confirm the presence of resistance to fluopyram in populations of *M. graminis* and *B. longicaudatus*. Nematode populations were collected from golf course locations in Florida where fluopyram had been repeatedly applied and showed reduced efficacy, as well as areas where fluopyram had never been used. These nematodes were then multiplied on bermudagrass in a greenhouse and continued to be exposed to fluopyram according to the (Indemnify®, Envu, Cary NC) turfgrass label or remained unexposed. In laboratory experiments, the populations of *M. graminis* and *B. longicaudatus* were exposed to increasing concentrations of fluopyram (0ppm, 10ppm, and 50ppm) for 24 and 72 hours. An NaOH solution was used to stimulate movement of live nematodes, and the percent mortality was recorded. The results of these experiments revealed that the populations of *M. graminis* and *B. longicaudatus* previously exposed to fluopyram were less affected by fluopyram *in vitro* than those with no previous fluopyram exposure, confirming fluopyram resistance in these populations. These, first ever, known fluopyram resistant plant-parasitic nematode populations will continue to be maintained for use in future experiments.

## Exploring the tripartite interactions of host, root-knot nematodes, and plant growth promoting bacteria for biocontrol

Habteweld, Alemayehu^1^, M. Kantor^2^, C. Kantor^3^ and Z. Handoo^1^

^1^Mycology and Nematology Genetic Diversity and Biology Laboratory, USDA, ARS, Northeast Area, Beltsville, MD, 20705

^2^Plant Pathology and Environmental Microbiology Department, Pennsylvania State University, University Park, PA 16802

^3^Huck Institutes of the Life Sciences, Pennsylvania State University, State College, PA 16802

### Abstract

Root-knot nematodes (RKNs) are the most widespread plant-parasitic nematodes in agricultural soils, infecting thousands of crops and causing annual losses of billions of dollars around the globe. Currently, the most effective and widely used RKNs control technique is the use of chemical nematicides. However, due to human health and environmental concerns, the use of many of these nematicides is banned or restricted. Therefore, there is a pressing need to develop effective and environmentally friendly alternative strategies for RKN control. One such alternative is the use of plant growth-promoting bacteria (PGPB), which facilitates RKNs’ control and increases soil fertility, plant growth, and crop safety. However, microbial agents that were found to be effective in controlling RKN in the laboratory and/or in the greenhouse conditions often do not replicate the same level of control in the more complex soil ecosystems. The low efficacy of microbial agents may be attributed to overlooking native microbiota that possesses protective abilities for RKN, as well as to soil abiotic factors that modulate the host-RKN-PGPB interactions. Consequently, a deeper understanding of the dynamics of host-RKN interactions in varied biotic and abiotic environments could be pivotal in devising novel RKN control strategies. Several pressing questions remain to be addressed. For instance: 1) How do we enhance our understanding of tripartite interactions to develop effective strategies for sustainable RKN management? 2) How to find the most effective RKN-PGPB species combination that enhances host fitness? 3) How does PGPB and RKN-protective microbiota competition influence the microbial composition in the rhizosphere? 4) What are the mechanisms RKNs use to recruit protective soil microbiota in soil? 5) What specific component of root exudates are involved in RKN and protective microbiota interactions? 6) What abiotic factors favor RKN-protective microbes? 7) Is RKN protective microbiota directly involved in infection and feeding site establishment? 8) Which agricultural practices may help to enhance the abundance and activities of indigenous PGPB, and their communication through volatiles? Finding answers to these questions could facilitate the effective integration of PGPB into strategies for sustainable RKN control and promote ecologically sound agroecosystems.

## Reconstruction of the esophageal connectome suggests rewiring of the nervous system in *Heterodera glycines*

Han, Jaeyeong^1^, A. Thompson^2^, J. Boudreaux^1^ and N. E. Schroeder^1^

^1^University of Illinois at Urbana-Champaign, Dept. of Crop Sciences, Urbana, IL 61801

^2^University of Illinois at Urbana-Champaign, School of Integrative Biology, Urbana, IL 61801

### Abstract

The soybean cyst nematode, *Heterodera glycines*, is a devastating pathogen that significantly affects soybean yields. The function of the plant-parasitic nematode esophagus is closely associated with pathogenicity such as feeding behavior and effector secretion. These vital functions are likely controlled by the esophageal nervous system. However, a lack of detailed anatomical data hinders our understanding of how the nervous system controls feeding in a plant-parasitic nematode. This study aims to map the esophageal connectome (a comprehensive map of neural connections) and anatomy of the infective second-stage juvenile (J2) of *H. glycines* using serial-section electron microscopy. We have obtained over 3,200 ultrathin (70 nm) serial transverse sections from two high-pressure frozen *H. glycines* J2s, covering their esophageal regions. The sections were collected on silicon wafers and imaged at a pixel resolution of 3.37 nm using a scanning electron microscope with a backscatter detector. The images were montaged and aligned for subsequent cell identification and segmentation. Our initial analysis suggests significant differences in synaptic connectivity between *H. glycines* and *Caenorhabditis elegans* neurons. Notably, the *C. elegans* interneuron I3 and its putative *H. glycines* homolog make different pre-and post-synaptic partners despite their morphological similarities. The *H. glycines* and *C. elegans* posterior bulb neurons exhibit divergent neurite morphologies. Specifically, *H. glycines* neurons are monopolar cells with an unbranched anterior process, whereas *C. elegans* neurons feature branched neurites or complex dorsal-ventral turns. These differences in wiring pattern and morphology may mediate behavioral differences between the two species. Interestingly, the *H. glycines* metacorpus is exceptionally similar to the *C. elegans* metacorpus in the number of nuclei, cell types, and morphology. For example, in the metacorpus of both species, pump muscles are characterized by a tri-symmetric structure anchoring marginal cells with finger-like interlocking structures. These similarities suggest the evolutionary importance of the metacorpus in diverged nematode clades. We expect this comprehensive mapping to reveal the neuronal connectivity and anatomy within the esophageal nervous system of *H. glycines*, providing insights into the structural basis of *H. glycines* feeding behavior. This study will help advance our understanding of nematode neuroanatomy and provide potential novel targets for control of plant-parasitic nematode species.

## Whole genome duplication facilitates anhydrobiosis in the fungal-feeding nematode *Aphelenchus avenae*

Han, Ziduan^1^, N. Yang^1^ and W-S. Lo^2^

^1^College of Plant Protection

^2^Institute of Future Agriculture. Northwest A&F University, Yangling, Shaanxi, China, 712100

### Abstract

Desiccation is a common challenge faced by all land animals. Some animals have evolved anhydrobiosis, a strategy that involves behavioral and morphological changes along with reduced metabolism, to tolerate drought conditions. The fungal-feeder *Aphelenchus avenae* has been used as a model to study anhydrobiosis in nematodes, due to its robust tolerance to drought. However, the molecular mechanism underlying anhydrobiosis remains unclear. To investigate this, we have assembled a high-quality genome of *A. avenae* using PacBio HiFi sequencing (yielding a genome size ~ 251 Mb, N50 = 10.1 Mb), and through synteny analysis we confirmed that this nematode has undergone a whole genome duplication (WGD) event during evolution. WGD might play a role in the evolution of anhydrobiosis in this nematode, although its contribution is yet to be demonstrated. Using Pluronic F-127 gel, we have developed a method that could slowly induce *A. avenae* to enter anhydrobiosis, allowing us to study different phases of the process. At 70% relative humidity, individuals of *A. avenae* started showing signs of entering anhydrobiosis after 22 hours, and almost all individuals entered anhydrobiosis by 66 hours. We sampled nematodes at pre-, mid-, and post-anhydrobiosis stage, and conducted RNAseq experiments to identify changes in gene expression. The results revealed drastic changes in gene expression between pre- and mid-anhydrobiosis. In addition, we found that both gene copies (ohnologs) of trehalose-6-phosphate synthase, a key enzyme in trehalose synthesis, were highly expressed 10 hours after induction of anhydrobiosis. Our ongoing work aims to further elucidate the transcriptomic changes during anhydrobiosis and understand the potential role of whole genome duplication in this process.

## Impact of varying population densities of the pale cyst nematode, *Globodera pallida* on susceptible and resistant potato

Hickman, Paige and L. M. Dandurand

Dept. of Entomology, Plant Pathology and Nematology, University of Idaho, Moscow, ID 83844

### Abstract

*Globodera pallida*, the pale cyst nematode is a regulated quarantine pest of potato in Idaho. It is important to understand and predict the potential impact of *G. pallida* in Idaho for growers, policymakers, and other stakeholders. Previous research showed that a population of 80 eggs/g soil resulted in an 80% predicted yield loss for the susceptible potato variety ‘Désirée’. This project expands this work to develop predictive models for the resistant potato variety ‘Innovator’ and susceptible variety ‘Russet Burbank’. Russet-type potatoes are the primary type grown in Idaho, whereas ‘Innovator’ is a highly resistant European variety that will be used as a model to determine how a resistant potato could be impacted by various population densities of the Idaho *G. pallida* population. Due to the regulated status of *G. pallida* in Idaho, it is not possible to directly assess yield impact in the field. Instead, these experiments were performed under greenhouse conditions on the susceptible varieties ‘Russet Burbank’, and ‘Désirée’, and a resistant variety, ‘Innovator’. Soil was infested with the Idaho *G. pallida* population at initial population densities of 0, 10, 20, 40, or 80 eggs/g soil. Fresh and dried biomass and yield data were collected. *Globodera pallida* cysts were extracted from soil and average eggs per cyst were determined to calculate the reproduction factor. From these data, the effect of initial *G. pallida* density on reproduction and potato yield were determined. The SUBSTOR-DSSAT software will also be used to simulate and create a model of *G. pallida* initial population density effect on yield for Idaho conditions. The relatively low reproduction on ‘Innovator’ indicates that this variety is highly resistant. Decreasing yield mass demonstrates that while ‘Innovator’ is resistant to *G. pallida*, it is not tolerant and suffers yield loss under high population densities. The high *G. pallida* reproduction rate observed on ‘Désirée’ and ‘Russet Burbank’ is indicative of a highly susceptible potato. Under these conditions, the overall yield of ‘Russet Burbank’ was unaffected by high *G. pallida* population densities. However, at 40 and 80 eggs/g soil, the average ‘Russet Burbank’ tuber size decreased by 50%, while the number of tubers increased. Thus, total yield was not impacted but quality of the tuber was greatly impacted by higher densities of this nematode. This research helps to build a better understanding of the Idaho *G. pallida* population’s ability to reproduce on susceptible russet-type potatoes or on resistant potatoes and its potential impact on plant health and yield.

## Investigating a potential inhibitive effect of high population densities on hatch of the pale cyst nematode, *Globodera pallida*

Hickman, Paige and L. M. Dandurand

University of Idaho, Dept. of Entomology, Plant Pathology, and Nematology, Moscow, ID 83844

### Abstract

*Globodera pallida* requires a chemical hatching factor typically from a host plant to cause hatch of dormant juveniles from eggs. As a survival strategy, not all eggs within a *G. pallida* cyst will hatch despite being exposed to a hatching stimulus. Some eggs will remain unhatched until exposure to a host in a subsequent season. The exact mechanism of this biological phenomenon is not well understood. Could there be a similar process where high population densities under limited resources decrease hatch of encysted eggs? Chance observations of remaining encysted eggs in previous experiments suggested that initial high population densities of *G. pallida* at 40 and 80 eggs/g soil may result in greater remaining viable encysted eggs of the original population used to infest soil. These high population densities could have some type of inhibitive effect on egg hatch as a survival mechanism when resources are too limited to support further infection in the roots. These observations coincide with progeny cysts increasing with increasing population density until about 40 and 80 eggs/g soil initial population, after which, the reproduction tapers off likely due to limited resources. *In vivo* experiments are being conducted where soil is inoculated with *G. pallida* at 2.5, 5, 10, 20, 40, and 80 eggs/g soil. The original population was sealed inside mesh bags to be recovered after 12 weeks of plant growth to determine the average remaining encysted eggs. *In vitro* hatch assays are also being performed to determine whether higher numbers of cysts within close proximity affect the percent hatch of the nematode. If high population densities of *G. pallida* inhibit hatching, this could have implications for *G. pallida* population dynamics over time.

## Resistance-breaking root-knot nematode-Fusarium disease complexes in tomato

Hodson, Amanda K.^1^, K. R. Paugh^2^ and C. L. S. Swett^2^

^1^Dept. Entomology and Nematology

^2^Dept. Plant Pathology, University of California Davis, Davis, CA

### Abstract

In the field, root-knot nematode infections rarely occur in isolation. Nematodes are known to form disease complexes with plant-parasitic fungi such as *Fusarium oxysporum,* the causative agent of Fusarium wilt. Combined infections of nematodes and fungi can increase plant mortality. Additionally, combined infections may result in disease synergy, where symptoms caused by the fungus are worse in the presence of nematodes than in plants infected with the fungus alone. While these effects have been examined in greenhouse and laboratory studies for over 40 years, few field studies have focused on the economic consequences of disease interactions between fungi and *Meloidogyne species* able to break resistance to the Mi gene. In California, processing tomatoes have traditionally been managed for nematodes and Fusarium wilt using resistant cultivars. However, resistance-breaking populations of root-knot nematodes have been acknowledged since 1996, and our recent research has shown that resistance-breaking populations of *Meloidogyne incognita* and *Meloidogyne javanica* were commonly isolated from cultivars known to have resistance to common biotypes of the nematode. An informal survey of 30 fields found that nematodes were more likely to be found on plants with an array of fungal diseases and were rarely isolated alone. In addition to root knot nematodes, plants were commonly infected with one or more fungal pathogen such as *F. oxysporum* f. sp. *lycopersici*-Fol (Fusarium wilt), *Fusarium falciforme* vine decline, and *F. oxysporum* f. sp. *radicis*-*lycopersici*-Forl (Fusarium crown and root rot). A greenhouse study where nematodes and Fusarium wilt were inoculated simultaneously did not find any disease synergy in symptoms, although plant biomass was lowered. This research suggests that root knot nematode/Fusarium disease complexes could be driving significant yield loss and that managing such co-infections requires consideration of both pathogens involved.

## Plant-parasitic nematodes associated with sweet pepper and pathogenicity of *Meloidogyne* spp. in Costa Rica

Humphreys-Pereira, Danny, F. Badilla-Jiménez, L. Núñez-Rodríguez and L. Flores-Chaves

Laboratory of Nematology-CIPROC, Agronomy School, University of Costa Rica, San José, Costa Rica 2060

### Abstract

Root galls in sweet peppers (*Capsicum annuum*) have been observed frequently in the Central Valley of Costa Rica (provinces of Alajuela, Cartago, Heredia, and San Jose). A survey of plant-parasitic nematodes was conducted in these four provinces. A total of 45 fields were sampled in a zig-zag pattern and included at least two composite samples (five plants per sample and the surrounding soil) per field. The two most frequent nematodes found in roots were *Meloidogyne* and *Helicotylenchus* with frequence of occurrence (FO) of 78.5% and 25.8%, respectively. Other nematodes found in roots were *Pratylenchus* (FO = 10.8%), Heteroderinae (FO = 6.5%), *Hemicycliophora* (FO = 4.3%), *Rotylenchulus* (FO = 4.3%), *Tylenchus* (FO = 4.3%), and Criconematidae (FO = 3.2%). Similarly, *Meloidogyne* and *Helicotylenchus* were also the most frequent nematodes found in soil with FO of 76.4% and 60.7%, respectively. In addition to the nematodes found in roots, Trichodoridae, *Ditylenchus*, *Paratylenchus,* and *Tylenchulus* were also observed in soil. Twenty-five *Meloidogyne* populations were identified at the species levels using the PCR-RFLP and PCR-species specific primers techniques. *Meloidogyne incognita*, *M. hapla,* and *M. exigua* were identified in 18, eight, and five fields, respectively. The species *M. incognita* and *M. exigua* were found concomitantly in three fields, *M. hapla* and *M. exigua* in one field, and *M. incognita* and *M. hapla* also in one field. A pathogenicity assay of *Meloidogyne* spp. on sweet pepper var. Nathalie was performed under greenhouse conditions to test the reproduction factor (RF) of the *Meloidogyne* species found in this study and *M. enterolobii*, an emerging pest. All tested *Meloidogyne* spp. had reproduction factor (RF) values from 7.7 to 25. To our knowledge, this is the first report of the coffee root-knot nematode, *M. exigua* parasitizing sweet pepper in Costa Rica. This information should be taken into consideration for intercropping systems such as coffee, where sweet peppers are grown between coffee trees after pruning.

## Antarctic nematode genetic structure reflects historical climate disturbances and geomorphic barriers

Jackson, Abigail^1^, S. D. Leavitt^1,2^, D. Porazinska^3^, D. H. Wall^4^, T. O. Powers^5^, T. S. Harris^5^ and B. J. Adams^1,2^

^1^Department of Biology, and Evolutionary Ecology Laboratories, Brigham Young University, Provo, UT 84602

^2^Monte L. Bean Life Science Museum, Brigham Young University, Provo, UT 84602

^3^Department of Entomology and Nematology, University of Florida, Gainesville, FL 32611

^4^Department of Biology and School of Global Environmental Sustainability, Colorado State University, Fort Collins, CO 80523

^5^Department of Plant Pathology, University of Nebraska – Lincoln, Lincoln, NE 68588

### Abstract

Historical climate disturbances, such as glacial cycling and fluctuating stream, lake, and sea levels, have a profound influence on the distribution and evolutionary trajectories of Antarctic terrestrial species. Antarctic invertebrates, including the ubiquitous sentinel nematode species *Scottnema lindsayae*, are particularly sensitive to climate disturbances. We tested hypotheses associated with the historical geographic and population genetic structure of this species as it occurs across the McMurdo Dry Valleys (MDVs) of Antarctica. To reconstruct the influence of climate disturbance and ecological conditions on this species, partial mitochondrial cytochrome c oxidase I (COI) gene sequences were sequenced and analyzed from individual *S. lindsayae* collected from sites across the MDVs reflecting opposing gradients of climate disturbance during the Last Glacial Maximum (LGM). We found that populations were strongly demarcated by geomorphic barriers, with distinct haplotypes associated with valleys except among valleys that experienced glacial advance and retreat during the LGM. Our work shows that contemporary populations of these animals are strongly structured by prior climate history and reinforced by subsequent ecological conditions. Such findings can be useful for understanding the processes that shape the distribution and abundance of these ecologically important animals and interpreting long-term monitoring of demographic shifts in response to changing climate trends in the McMurdo Dry Valleys.

## Vegetable farm cover crop practices to improve nematode management, nitrogen utilization and to support water quality improvement in Florida

Jacobs, Dustin^1^, J. Desaeger^1^, M. Lusk^2^ and Z. Grabau^3^

^1^University of Florida, Gulf Coast Research and Education Center, Wimauma, FL 33598

^2^University of Florida, Steinmetz Hall Gainesville, FL 32611

### Abstract

Cover crops in agricultural systems pose many potential benefits including erosion reduction, nutrient cycling, weed suppression, and nematode suppression. These benefits have the potential to reduce chemical inputs and synthetic fertilizer use in the cash crop season, as well as improve soil and water quality by reducing nitrogen leaching. Further research is needed to determine the effect of cover crops on these factors, especially in sandy soils found in Florida. The objective of this study is to observe the ability of cover crops to improve nitrogen utilization, reduce nitrogen leaching, and increase nematode suppression. Nonlegume cover crops (sorghum sudnangrass) are expected to provide less soil nitrogen than their legume counterparts (cowpea, sunn hemp). However, nonlegume crops are expected to reduce nitrogen leaching more than legume crops. All cover crop treatments are expected to improve nitrogen utilization and reduce leaching compared to weedy fallow. A two-year experiment (2023–25) will be conducted to compare the effect of 6 treatments on these factors. Cover crops are sunn hemp (*Crotalaria juncea*), sorghum sudangrass (*Sorghum bicolor × S. bicolor* var. sudanense), cowpea (*Vigna unguiculata*), a 1:1 sunn hemp and cowpea mix, a 1:1 sunn hemp and sorghum sudangrass mix and a weedy fallow serving as the control. In the first year, cover crops were planted late April 2023 using a randomized complete block design in 7.3 m × 91.4 m plots with six replicates. Cover crops were mowed and incorporated early August and three, 100 m raised plastic-mulch beds were formed perpendicular to the cover crop blocks in late August. Six-week-old tomato (cv. HM1823) seedlings were planted in 30 ft plots (15 plants per plot), replicated six times. Nitrogen utilization was measured by observing mineralization rates found in soil samples taken on seven sample dates after cover crop destruction (0, 2, 3, 6, 8, 12, and 18 weeks after destruction) at three depths (0–15, 15–30, 30–45 cm). Using the same soil samples, nitrogen leaching was measured by observing the extractable NO_3_^−^ and NH_4_^+^ movement over time between different depths of soil samples in each treatment. Plant-parasitic and non-plant-parasitic nematode populations were measured in 200 cm^3^ of soil from the same samples as taken for nitrogen data. Nitrogen and nematode data are still being processed and analyzed. Preliminary nematode data showed that nematode populations at the end of the cover crops were predominantly ring nematodes (Criconematidae) and lesion nematodes (*Pratylenchus* spp.). Root-knot nematodes were found at the end of the tomato crop and minor to medium root gall incidence was noted on tomato roots. Only minor differences were noted among treatments. The trial will be repeated in 2024 on the same land using identical treatments.

## Efficacy of organic nematicides against *Meloidogyne incognita* infecting cucumbers under field conditions

Jagdale, Ganpati^1^, A. Hajihassani^2^ and D. Shapiro-Ilan^3^

^1^Dept. Plant Pathology, University of Georgia, Athens, GA

^2^Dept. Entomology and Nematology, Fort Lauderdale Research and Education Center, University of Florida, Davie, FL

^3^USDA-ARS, Byron, GA 31008

### Abstract

Cucumber (*Cucumis sativus*) is a major market vegetable crop grown in the U.S., including Georgia. The root-knot nematode (RKN; *Meloidogyne incognita*) is considered an important pest of vegetables, including cucumber. Pre-plant soil fumigation with chemicals is commonly used to manage plant-parasitic nematodes (PPNs), especially RKNs, in many vegetable production systems. Due to environmental or regulatory concerns, alternative control methods are needed, especially nematicidal products that can effectively target PPNs while being safe for humans and the environment. We compared the efficacies of metabolites from two entomopathogenic bacteria (*Xenorhabdus szentirmaii* and *X. bovienii*) associated with entomopathogenic nematodes, each applied at a rate of 7.34 liters per plot, Majestene™ (a.i. Heat-killed *Burkholderia* spp. strain A396) at 7.57 liters per acre, MeloCon WG (*a.i.Paecilomyces lilacinus* strain 251) at 1.81 Kg per acre, MeloCon LC (*a.i. Paecilomyces lilacinus* strain 251) at 0.30 liters per acre, Vydate (*a.i.* oxamyl) at 1.89 liters per acre, and an untreated check (water) against *M. incognita* infestation in cucumber variety Mongoose grown on plastic beds in 2021. The treatments were injected through the drip irrigation system using a CO_2_-pressurized tank during the transplanting of three-week-old cucumber seedlings and arranged in a randomized complete block design with five replicates per treatment. Population densities of RKNs, root-galling indices using a 0–5 scale (0 = no galls seen, 5 = roots completely covered in galls), plant growth parameters, and cucumber yields were determined 60 days after treatment (DAT). Soil population densities of RKNs were significantly reduced in all treatments, including Vydate, Majestene, MeloCon and the metabolites of both *X. szentirmaii* and *X. bovienii* bacteria, compared to the untreated check at 60 DAT. However, the indices of root galls caused by RKN were significantly reduced only in plots treated with Vydate compared to the untreated check at 60 DAT. We observed that the number of cucumber fruits was not statistically increased by the application of nematicides, but fruits were numerically higher in the plots treated with Vydate, Majestene, MeloCon, and the metabolites of both *X. szentirmaii* and *X. bovienii* bacteria than in plots treated with MeloCon and the untreated check. A similar trend was observed in the weight of cucumber fruits. These results support our previous greenhouse findings (Data not provided) that all the treatments except MeloCon were effective in reducing populations of RKNs. This suggests that biologically active nematicides such as Majestene and the metabolites of entomopathogenic bacteria are effective in suppressing RKN populations infesting cucumbers.

## Development of bionematicide formulation using vetiver extract for controlling root-knot nematodes in chilli

Jindapunnapat, Kansiree^1^, V. areedenchai^2^, D. Sakloetsakun^3^, W. Phattanaphakdee^2^, N. Chomphuphuang^1^, K. Poruangdate^4^, A. ongcharoen^1^ and B. Chinnasri^5^

^1^Department of Entomology and Plant Pathology, Faculty of Agriculture, Khon Kaen University, Thailand

^2^Dept. Pharmacognosy, Faculty of Pharmacy, Srinakharinwirot University, Thailand

^3^Dept. Pharmaceutical Technology, Faculty of Pharmaceutical Sciences, Khon Kaen University, Khon Kaen, Thailand

^4^Office of Agricultural Regulation Department of Agriculture, Thailand

^5^Dept. Plant Pathology, Faculty of Agriculture, Kasetsart University, Thailand

### Abstract

*Vetiver zizanioides* (L.) Nash, belonging to the family Poaceae, provides a variety of usage including protecting soil against erosion, or serving as thatched roof and herbal medicines. ‘Songkha 3’ vetiver grass collected from different sites was found to contain *p*-coumaric acid and has been reported to suppress nematodes. The mortality rate of root-knot nematodes was examined on these compounds at the laboratory level. It was determined that *p*-coumaric acid was able to kill 69.86 % of root-knot nematodes within 24 hours, compared to 36.78 % that in vetiver extracts. It was revealed that *p*-coumaric acid belongs to a group of phenolic compounds. Natural vetiver extract had total phenolic content (TPC) of 53.27 – 98.47 ug/mg and total polysaccharide contents (TSC) of 163.25 to 329.58 ug/mg. Laboratory and greenhouse experiments were conducted to determine the efficacy of vetiver extract in suppressing root-knot nematodes infecting chili plants. It was found that vetiver extract had a killing effect of >80 %, it inhibited root-knot nematode development >70 %, and decreased egg number per g root >80 % without imposing phytotoxicity to chili plants. In addition, it also enhances the growth of chili when used as green manure and as soil amendment. Spray-dry formulation of vetiver extract was evaluated in the laboratory. It was determined that a 50 mg/ml concentration was able to kill root-knot nematodes as effective as the pure vetiver extracts. When the formulation was applied to chili plants in a laboratory experiment, the aforementioned application rate reduced the number of eggs per root weight by 90.14 %, which was the same as the vetiver extract. Therefore, vetiver grass can be used to produce a solution to tackle root-knot nematodes. Farmers facing root-knot disease in chili fields will benefit from using vetiver grass extract as spray in the future.

## Living nematodes in America’s dead sea

Jung, Julie, T. Loschko, S. Reich, M. Rassoul-Agha and M. S. Werner

Dept. Biological Science, University of Utah, Salt Lake City, UT 84112

### Abstract

Extreme environments enable the study of simplified food-webs and evolutionary bottlenecks. We investigated the biodiversity of invertebrate meiofauna in the benthic zone of the Great Salt Lake (GSL), Utah, one of the most hypersaline lake systems in the world. The GSL is currently thought to support only two multicellular animals: brine fly larvae and brine shrimp. Here, we report the presence, habitat, and microbial interactions of novel free-living nematodes. Nematode diversity drops dramatically along a salinity gradient from a freshwater river into the south arm of the lake. In Gilbert Bay, nematodes primarily inhabit reef-like organosedimentary structures built by bacteria called microbialites. These structures likely provide a protective barrier to UV and aridity, and bacterial associations within them may support life in hypersaline environments. Notably, sampling from Owens Lake, another terminal lake in the Great Basin that lacks microbialites, did not recover nematodes from similar salinities. Phylogenetic divergence suggests that GSL nematodes represent previously undescribed members of the family Monhysteridae, one of the dominant faunas of the abyssal zone and deep-sea hydrothermal vents. These findings update our understanding of halophile ecosystems and the habitable limit of animals.

## Entomopathogenic nematodes avoid the scent of predatory mites

Kamali, Shokoofeh, D. Olabiyi, L. L. Stelinski, L. Diepenbrock and L. Duncan

University of Florida, IFAS, Citrus Research and Education Center, 700 Experiment Station Road, Lake Alfred, FL 33850

### Abstract

The utility of entomopathogenic nematodes (EPNs) in integrated pest management programs is limited by their poor persistence in soil. Abiotic soil conditions such as temperature, moisture, and texture modulate the persistence of EPNs, and top-down regulation by predators and diseases may also drive survival rates. To better understand how EPNs cope with microarthropod predators, we conducted assays to measure chemotactic responses of the EPN, *Heterorhabditis bacteriophora*, *Steinernema feltiae*, *Steinernema diaprepesi*, and *Heterorhabditis indica* to the mesostigmatid mite, *Stratiolaelaps scimitus*. The numbers of IJ consumed by *S. scimitus* did not differ between EPN species in any experiment. By contrast, *S. scimitus* consumed between 3-fold and 5-fold as much biomass when presented with steinernematid species compared to heterorhabditids. When the nematode IJs were placed in two-choice *T*-tube assay units filled with washed sand (>150μ), *H. bacteriophora* and *S. diaprepesi* moved away from *S. scimitus.* Using a ‘push–pull’ headspace collection system combined with gas chromatography-mass spectrometry (GC-MS), two citral isomers, neral, and geranial, were recovered from *S. scimitus*. Both compounds were repellent to *H. bacteriophora* and *S. diaprepesi* in *T*-tube assays. Research to detect additional EPN semiochemicals that may interact with nematode response to these compounds is ongoing. Our results suggest that EPN have evolved the ability to detect and respond to neral and/or its isomers as a kairomone indicating danger (i.e. predation).

## X-ray photoelectron spectroscopy analysis for the chemical impact of *Litylenchus crenatae mccannii* infection on american beech (*fagus grandifolia* ehrh.)

Kantor, Mihail^1^, M. Goraya^1^, C. Kantor^2^ and A. Marin^3^

^1^Plant Pathology and Environmental Microbiology Department, Pennsylvania State University, University Park, PA 16802

^2^Huck Institutes of the Life Sciences, Pennsylvania State University, State College, PA 16802

^3^Ken and Mary Alice Lindquist Dept. Nuclear Engineering, Penn State University, University Park, State College, PA 16802

### Abstract

*Litylenchus crenatae mccannii* (Lcm) is the pathogen responsible for beech leaf disease (BLD), posing a significant threat to American beech trees and Northeastern Forest ecosystems. BLD symptoms include dark-green interveinal banding in leaves, leaf thickening, and aborted buds, ultimately leading to tree mortality. The distribution of non-structural carbohydrates (NSCs) is crucial for the physiological processes and metabolic functions involved in tree growth and the defense mechanisms needed for survival. However, a significant gap remains in understanding how NSCs are allocated after sustained physical damage caused by nematodes feeding on different parts of the beech trees, such as leaves and buds, throughout their growth cycle. By the ability to distinguish between mixed carbon species, the X-ray photoelectron spectroscopy (XPS) technique was employed to study the chemical behavior of both infected and non-infected trees. An increased oxygenated carbon response, namely C-O and C=O/O-C-O functional groups, coupled with an increased oxygen-to-carbon ratio, was found in the diseased trees. In addition, the oxygen species follow a similar tendency, as the C=O/O=C-O chemical groups are increasing compared to healthy trees. Traces of phosphorus (~0.3%) were also detected and attributed to C-PO_3_ chemical species. This work is intended to establish the chemical mechanism of carbon oxidation in response to nematode activity.

## Novel bacteria for biological control of root-knot nematode, *Meloidogyne incognita*

Kassam, Rami^1^, U. Rao^2^ and A. ajihassani^1^

^1^University of Florida, Dept. of Entomology and Nematology, Fort Lauderdale Research and Education Center, Davie, FL 33314

^2^Indian Agricultural Research Institute, Division of Nematology, Delhi, India 110012

### Abstract

Among microorganisms that parasitize or reduce populations of plant-parasitic nematodes (PPNs) by their antagonistic behavior, bacteria play an important role and certain species/strains have shown considerable promise as biocontrol agents, offering a potential solution for managing PPNs. The objective of this study was to reinvestigate the local bacterial diversity across selected states in India, with a focus on identifying species that play a key role in maintaining the natural balance of nematode populations and exploiting the potential ones for root-knot nematode management. Soil samples were collected from 13 Indian states from the rhizosphere of fruit trees, pulses, cereals, and vegetables and used for isolating potential bacteria employing *Meloidogyne incognita* and *Caenorhabditis elegans* as baits in water agar. Isolation and purification of various bacteria followed by molecular diagnoses based on the sequencing of 16s rRNA gene revealed the presence of some predominant genera belonging to both plant pathogenic and non-pathogenic groups, including *Alcaligenes faecalis, A. aquatilis, A. endophyticus, Burkholderia cepacia, B. ambifaria, Pseudomonas entomophila, P. mosselii* and *Pseudochrobactruma saccharolyticum. In vitro* evaluation of different concentrations of the culture filtrates of the isolated bacteria against *M. incognita* revealed their potential to kill second-stage juveniles by up to 96.3% and 89.1% in 100% and 25% concentrations, respectively at 2 days after treatment. GC-MS analysis of the culture filtrates revealed the presence of nematicidal compounds such as Heneicosane, Myristynoyl pantetheine and Dimethyl disulfide in most of the bacteria. Further evaluation of the bacterial cultures with high mortality effects against *M. incognita* infecting tomato plants under greenhouse conditions showed that the *A. faecalis* isolate significantly reduced numbers of galls, females, and egg masses of *M. incognita* by 75.3%, 72.3%, and 96.7%, respectively compared to the untreated check. Additional evaluation of the promising bacteria under field conditions will support commercial development and deployment of effective biocontrol agents to combat root-knot nematodes.

## Nuclear receptors expressed in the *Pristionchus* stoma polyphenism are lineage-specific metabolic regulators

Katsougia, Eleni, S. J. Connors and E. J. Ragsdale

Department of Biology, Indiana University, Bloomington, IN 47405

### Abstract

Nuclear receptors (NRs) include important mediators between environmental signals and the outcomes of plastic development. In the case of polyphenism, whereby developmental plasticity results in categorically alternative forms, such factors are predicted to allow switching between alternative gene networks. In the nematode *Pristionchus pacificus*, cues such as starvation and crowding promote the development of alternative forms of the stoma, allowing access to different food resources when induced. NRs function as sensors to rapidly respond to endogenous or exogenous signals. These transcription factors have undergone an extensive expansion in nematodes, although the evolution and the molecular functions of most of these in invertebrates are still unknown. Thus far, two NRs (NHR-40 and NHR-1) that regulate the polyphenism have been uncovered through forward genetic screens. Further, co-expression networks influenced by these and other known polyphenism switch genes have revealed potentially subordinate NRs in this plastic developmental response. Specifically, these NRs belong to polyphenism-biased co-expression modules and whose expression was controlled by multiple morph-constitutive lines. However, whether and how such transcription factors affect the outcome of a polyphenism is unknown, as are their effects on associated processes of metabolism, physiology, and behavior. To test whether these NRs influence phenotypes associated with the *Pristionchus* resource polyphenism, we used CRISPR/Cas9 gene editing to determine the NRs’ functions. As a result, we found that several of these NRs affect fat metabolism and storage, consistent with a role for the polyphenism in accessing alternative diets. Moreover, we found that these NRs were the product of gene duplications specific to a lineage of polyphenic nematode species, suggesting rapid turnover in transcription factors subordinate to the polyphenism switch. In summary, our results give insight into how alternative gene-networks faithfully execute a polyphenism decision and how this decision is linked to the development and physiology of the organism.

## High prevalence of *Wolbachia* and *Cardinium* revealed in different geographical populations of plant-parasitic nematodes

Kaur, Amandeep and A. M. V. Brown

Dept. Biological Sciences, Texas Tech University, Lubbock, TX

### Abstract

Plant-parasitic nematodes (PPNs) pose a significant threat to global agriculture, causing substantial yield losses. *Wolbachia* and *Cardinium*, two endosymbionts reported in PPNs, exhibit diverse roles ranging from mutualists to reproductive manipulators in different hosts. Despite their various functions observed in non-PPN hosts, their distribution, prevalence, diversity, and roles in PPNs remain poorly known. To investigate the prevalence of these endosymbionts in PPNs, we developed a novel PCR assay targeting PPN-type *Wolbachia* and *Cardinium*. Primers were designed using Geneious Prime with alignments to PPN-type strains recently discovered or extracted from database mining. Bulk nematode communities isolated from soil and root samples collected from 232 sites from 21 US states were screened for *Wolbachia* and *Cardinium*. Individual nematodes were also isolated from farms in Washington (WA) and DNA was extracted using a single worm lysis protocol. Of the screened bulk nematode communities, 45% were positive for *Wolbachia* and 60% were positive for *Cardinium*. Single nematode screening for *Pratylenchus penetrans* from one WA population showed *Wolbachia* at 45.8% prevalence and *Cardinium* at 63.5% prevalence with 40.6% of individuals being co-infected. Another population from a nearby raspberry field in WA had lower infection rates, with *P. penetrans* infected with *Wolbachia* at 9.8% and *Cardinium* at 27.4%, whereas this community showed other unidentified PPNs had *Wolbachia* at 15% and *Cardinium* at 24%, with co-infections at 7.3%. Our findings highlight the utility of the novel PCR assay on single worm as well as bulk communities in detecting *Wolbachia* and *Cardinium* infections in PPNs. The prevalence observed hints at the richness of symbiotic relationships within nematode communities. Ongoing work includes metagenomic sequencing to determine the functional profiles of the new PPN *Wolbachia* strains. Further comparative genomics will investigate critical genes with host-manipulation functions that may inform strategies to aid symbiont-based integrated pest management and biocontrol.

## Soil temperatures differentially impact reproduction biology of nematodes

Khanal, Churamani^1^, J. Land^2^, P. Banakar^3^ and S. GC^1^

^1^Plant and Environmental Sciences Department, Clemson University, Clemson, SC

^2^Rheinland-Pfälzische Technische Universität, Landau, Germany

^3^CCS Haryana Agricultural University, Department of Nematology, Hisar, Haryana, India

### Abstract

Addressing the adverse impacts of climate change on agriculture for ensuring global food security is one of the major challenges of the millennium. While increase in soil temperature due to climate change is known to increase stress on crops, the fate of nematode and cop-nematode relationships under higher soil temperatures is not fully understood. A series of studies were conducted to understand the impacts of various soil temperatures on reproduction biology of two nematode species: the reniform nematode (*Rotylenchulus reniformis*) and the peach root-knot nematode (*Meloidogyne floridensis)*. In one experiment, a commercial heat mat was used to raise soil temperatures to 32 (control), 33, and 34°C for 7 hr during the daytime, and subsequent evaluation of nematode reproduction, virulence, and/or disease severity on tomato (cv. Rutgers) at six weeks after inoculation in a growth room. The reproduction of *R. reniformis* decreased significantly at 33°C relative to the control, however, the reproduction of *M. floridensis* was statistically similar at both temperatures. Relative to 33°C, the reproduction of *R. reniformis* remained statistically similar to that at 34°C while that of *M. floridensis* was significantly reduced. At 34°C, the reproduction of *R. reniformis* and *M. floridensis* decreased by 49% and 53%, respectively relative to that at control. Additionally, the virulence of *R. reniformis* was increased when soil temperature was increased from 32 to 34°C suggesting a greater virulence of the nematode at higher soil temperatures. The virulence of *M. floridensis* appeared to be decreased as evident from increased root biomass when soil temperature increased from 32 to 34°C, however, the increased root biomass resulted from increased root galling at the higher temperature. In the second experiment, soil temperatures of 26 (control), 32, 34, and 36°C were established using the commercial heat mat in the growth room and nematode reproduction and virulence of *R. reniformis* and *M. floridensis* on tomato (cv. Rutgers) were evaluated after six weeks of inoculation. The reproduction of both nematodes significantly increased only at 34°C relative to the control although root biomass remained statistically similar. Root biomass of *R. reniformis* infected plants were significantly reduced at 32 and 36°C while those infected with *M. floridensis* had significantly lower biomass at the latter temperature only. Results suggested that soil temperatures affect nematode reproduction biology, and the effect is differently expressed in nematode species.

## Identification and characterization of a virulent *Meloidogyne incognita* population breaking the tomato *Mi*-1.2-mediated resistance in Indiana

Kunwar, Vijay^1^, W. Guan^2^ and L. Zhang^1,3^

^1^Dept. Botany and Plant Pathology, Purdue University, West Lafayette, IN 47907

^2^Dept. Horticultural and Landscape Architecture, Purdue University, Southwest Purdue Agricultural Center, Vincennes, IN 47591

^3^Dept. Entomology, Purdue University, West Lafayette, IN 47907

### Abstract

High tunnel production is increasing rapidly in the US because of its effectiveness in extending production seasons. Tomato is considered one of the most profitable crops grown in high tunnels. However, high crop density, elevated soil temperature and constant soil moisture in high tunnels lead to the buildup of plant-parasitic nematodes (PPN) in soils. Among them, root-knot nematodes (RKNs) have a broad host range and damage roots of tomatoes and other specialty crops. Growing RKN-resistant tomato cultivars or using the grafting technique with RKN-resistant tomato rootstock are considered effective in controlling RKNs. Unfortunately, all the RKN-resistant tomatoes carry the same resistance gene *Mi*-1 and this lack of diversity in resistance can lead to the emergence of virulent RKN populations. In this study, we identified a virulent population of the southern root-knot nematode (*Meloidogyne incognita)* in a high tunnel in Indiana. The *M. incognita* population was confirmed as being able to infect and reproduce on two resistant tomato cultivars, ‘Better Boy’ and ‘Early Girl’, both carrying the *Mi*-1 gene, under a controlled environment at 24°C. To our knowledge, this is the first report of *Mi*-1.2-gene resistance-breaking *M. incognita* population in Indiana. The RKN-12 virulent population exhibited equal penetration of J2 on both susceptible and resistant cultivars. However, there was a notable impact on development in the resistant cultivar, with only 8.2% and 6% of the initial RKN-12 inoculum progressing to young and adult females on the resistant cultivars, Early Girl, and Better Boy, respectively, in contrast to the 46% on the susceptible cultivar Rutgers at 45 dpi. The reproduction factors (Rf) of RKN-12 on the two resistant tomato cultivars, Better Boy, and Early Girl, were 3.28 and 2.67, respectively at 45 dpi. However, when considering the virulent sub-population, the Rf values surged to 14.03 and 13.59 for Better Boy and Early Girl, respectively at 45 dpi. The findings suggest that virulent populations have the capacity to rapidly proliferate, even in resistant cultivars and thus necessitates the high tunnel growers to regularly monitor the RKN population density and virulence to devise strategies to manage RKNs effectively.

## Determining host susceptibility of cover crops to reniform nematodes

Larger, Kekoa, R. Paudel, B. Wiseman and K.-H. Wang

Dept. Plant and Environmental Protection Sciences, University of Hawaii at Manoa, Honolulu, HI 96822

### Abstract

Reniform (*Rotylenchulus reniformis*) nematode is a key economic plant-parasitic nematode of sweet potato. Planting cover crops that are not susceptible to this nematode could mitigate pest pressure and improve sweet potato yield. The objective of this project is to determine the host susceptibility of 4 cover crops: ‘Nemagone’ marigold (MG, *Tagetes patula*), velvet bean (VB, *Mucuna pruriens*), ‘NX-D-61’ (NX-2) energy sorghum (*Sorghum bicolor*), and ‘Latte’ (LA) sorghum-sudangrass hybrid (*Sorghum bicolor × S. bicolor* var. Sudanese) to reniform nematodes. Two greenhouse pot trials were conducted. Trial I used a sterile sand/soil mix inoculated with 180 juveniles of reniform nematode/pot; Trial II used field soil infested with 640 reniform juveniles/pot. Each cover crop was replicated in 4 pots/trial. Six weeks after inoculation, reniform nematode eggs and juveniles in the roots were lower (*P* < 0.05) in all cover crops tested compared to the susceptible cowpea control in Trial I. Among which, VB was the only treatment with a reproductive factor (RF = final nematode counts/initial inoculation rate) lower than 1, indicating a lack of reniform reproduction in VB. The same result was recorded in Trial II with all cover crops having less reniform juveniles and eggs than the susceptible cowpea control (*P* < 0.05). These results confirmed the use of these four nematode suppressive cover crops in rotation with sweet potato to reduce reniform nematode initial population densities at planting that is challenging the sweet potato farmers in Hawaiʻi. Since VB is very promising in resisting the development of reniform nematodes, many farmers are interested in using VB as a cover crop to suppress plant-parasitic nematodes. Thus, the next logical step would be to evaluate the susceptibility of different varieties of VB against root-knot (*Meloidogyne incognita)* and reniform nematodes to address the interest of farmers.

## Exploring the diversity of turfgrass-associated entomopathogenic nematodes and their symbiotic bacteria in Florida for utilization of bacterial metabolites for nematode control

Larkin, Jacob^1^, R. Kassam^1^, W. Crow^2^ and A. Hajihassani^1^

^1^Dept. Entomology and Nematology, Ft. Lauderdale Research and Education Center, University of Florida, Davie, FL 33314

^2^Dept. Entomology and Nematology, University of Florida, Gainesville, FL 32611

### Abstract

Research suggests the nematicidal potential of toxic cell-free bacterial metabolites isolated from the symbiotic bacterial complex of entomopathogenic nematodes (EPNs) against plant-parasitic nematodes (PPNs). In this study, we determined the diversity of EPNs and their symbiotic bacteria in turfgrass systems across Florida to investigate the nematicidal effects of a diverse set of isolated bacterial metabolites. Soil samples collected from various turfgrass systems were used to isolate EPNs by adding *Galleria mellonella* larvae into the soil and then extracting EPN populations from symptomatic infected larvae using the standard White traps. EPN populations were identified by targeting the 28s rRNA and *ITS*-*rDNA* genomic *regions.* Furthermore, symbiotic bacteria were directly extracted from the recovered infective juvenile EPNs and identified using PCR amplification of 16s rRNA gene. Recovered PCR products from EPNs and bacteria were sequenced, and species identification *was* performed using a BLASTn search based on the similarity of the available sequences in GenBank. Three species of EPN *Steinernema glaseri*, *Steinernema diaprepesi*, and *Heterorhabditis indica* were detected in 12 out of 100 soil samples analyzed, with a regional breakdown of 7 in central Florida, 3 in northern Florida, and 2 in southern Florida. Several bacterial strains were found associated with the EPNs; *Photorhabdus luminescens, P. akhurstii, Xenorhabdus doucetiae, Stenotrophomonas maltophilia, Pseudochrobactrum saccharolyticum*, *Alcaligenes faecalis*, and *Ochrobactrum* spp. All the identified bacteria were cultured in nutrient media and cell-free metabolites were extracted from each via filtration. The cell-free metabolites were investigated for their nematicidal potential against the most economically damaging turfgrass-associated PPNs in Florida, sting (*Belononlaimus longicaudatus*), lance (*Hoplolaimus gelateus*), and root-knot (*Meloidogyne graminis*) nematodes. A primary bioassay showed 100% mortality of these three nematodes when subjected to 100% concentration of each of the metabolites after 24, 48, and 72 hours. Further study will be conducted using lowering concentrations to determine the metabolite toxic effects on these nematodes. The utilization of these bacterial metabolites against PPNs may represent a biologically derived alternative to traditional synthetic nematicides.

## Utilizing seasonal population dynamics of plant-parasitic nematodes for improved management strategies in Southern Florida Golf courses

Larkin, Jacob and A. Hajihassani

Dept. Entomology and Nematology, Ft. Lauderdale Research and Education Center, University of Florida, Davie, FL 33314

### Abstract

Golf is a popular outdoor recreational activity that provides enjoyment for Florida’s visitors and residents alike while providing large boons to the state’s economy. As such, considerable interest and investment goes into maintenance for their turfgrass playfields. Plant-parasitic nematodes (PPNs) represent a significant damage potential for golf course turfgrass through the direct root damage of their feeding, or through the indirect damage of predisposing the weakened turf to other diseases after feeding. Florida’s golf courses primarily rely on the warm season turfgrass species, bermudagrass (*Cynodon* spp.), which can be susceptible to a number PPNs. The most damaging of which are lance (*Hoplolaimus galeatus*), sting (*Belonolaimus longicaudatus*), and root-knot nematodes (*Meloidogyne graminis*), otherwise known as the “Big Three.” Current management strategies, which include seasonal or reactive chemical applications, often lack specificity, and may not align with the actual presence of damaging PPN levels, leading to inefficiency and potential overuse of nematicides. The goal of this research was to examine the yearly population dynamics of important PPNs in southern Florida golf courses for the purpose of creating a more efficient nematicide timing application protocol tailored to this region. From the period of October 2022 to October 2023, monthly soil sampling was conducted at three active golf courses and one golf course-like control site in southern Florida. At each course, six sub-locations were selected based on previous history of nematode infestation, management practices, and observed nematode damage symptoms. Five soil cores were collected at each sub-location once a month at a depth of 12 inches. From the soil cores, nematodes were extracted using a traditional sugar flotation extraction method and identified and counted to a genus level using an inverted microscope. Nine genera of PPN were found in each of the four locations, lance (*Hoplolaimus* spp.), sting (*Belonolaimus* spp.), root-knot (*Meloidogyne* spp.), ring (*Mesocriconema* spp.), spiral (*Helicotylenchus* spp.), shethoid (*Hemicriconemoides* spp.), dagger (*Xiphenema* spp.), stunt (*Tylenchorhynchus* spp.), and stubby-root (*Trichodorus* spp.) nematodes. Samples containing individuals from the lance, sting, and root-knot genera were identified further to a species level using DNA sequencing techniques and the following were found: *Hoplolaimus galeatus*, *Belonolaimus longicaudatus*, and *Meloidogyne graminis*. Focusing on the “Big Three” PPNs, population numbers were modeled with the month they were collected. We found that lance and sting nematodes had the largest populations during the early spring and for root-knot nematodes, the highest population was in the middle of the summer. Based on these findings, an experimental nematicide application protocol was developed and will be implemented in 2024, aiming for more precise timing to enhance effectiveness and efficiency compared to standard industry practices.

## Evaluation of different nematicide rates and application intervals against plant-parasitic nematodes associated with turfgrass in South Florida

Lasa, Michelle, A. and A. Hajihassani

Dept. of Entomology and Nematology, Fort Lauderdale Research and Education Center, University of Florida, Davie, FL 33314

### Abstract

Plant-parasitic nematodes (PPNs) are detrimental to the overall health and function of turfgrass across South Florida. The wide variety of PPN species that utilize turfgrass as a host in combination with the lack of definitive signs and symptoms of infection make the control of PPN difficult. The method primarily used to reduce and control the population of PPNs in turfgrass has been the use of chemical nematicides. However, the efficacy of existing chemical nematicides with the same mode of action is currently at risk due to the potential of resistance development in their target PPNs. As a result, there is a current need to test new chemistries with new modes of action for the management of PPN populations. In this study, we compared the efficacy of three distinct nematicides (Resilia, Kitae, and Indemnify), each differing in their active ingredient, rate or application interval which consisted of six treatments [(Resilia A: 14-day interval and Resilia B: 28-day interval both at 4 oz/1000 sq ft), (Kitae A: 24 oz/acre and Kitae B: 48 oz/acre both with 28-day interval), (Indemnify: 0.39 oz/1000 sq ft with 28-day interval), and (non-treated control)]. In order to find the optimal rate and timing of application for the management of PPNs, a randomized complete block design was used with five replicates per treatment. The PPN densities were assessed three times (before nematicide application, 58 days, and 112 days after application). Four PPNs (*Meloidogyne graminis*, *Belonolaimus longicaudatus*, *Hoplolaimus gelateus*., and *Trichodorus* spp.) were detected with *H. gelateus* having the highest densities in contrast to *B. longicaudatus* which had the lowest densities on average. There was a reduction (*P* < 0.05) of *M. graminis* and *H. gelateus* found 58 and 112 days after the first nematicide application when compared to the initial population. Although not different from the control, Indemnify and Kitae (B) had the lowest (*P* < 0.05) *H. gelateus* densities, while Resilia (A and B) and Kitae (A) had the highest densities. There was no significant reduction of *B. longicaudatus*, *M. graminis*, and *Trichodorus* spp. among the treatments. However, Indemnify had the lowest *M. graminis* densities, Kitae (B) and Resilia (A) had the lowest *Trichodorus* spp., and Resilia (A) had the lowest *B. longicaudatus* densities. These results suggest that nematicide effectiveness is influenced by active ingredients, rate, and application intervals. We are currently repeating this trial to confirm the potential efficacy differences found in the treatments for the management of detected PPNs as it was shown to reduce population densities. Ultimately, this research will help turfgrass managers identify and utilize alternative methods of nematode management.

## Metabolic insights into root-knot nematode infestations: unraveling nutrient exchange between parasites and host plants

Latina, Romnick A.^1^ and S. Siddique^2^

^1^Dept. Plant Pathology, University of California Davis, Davis, CA

^2^Dept. Entomology and Nematology, University of California, Davis, Davis, CA

### Abstract

Root-knot nematodes are highly destructive agriculture pests causing substantial yield losses across millions of hectares annually, significantly impacting crop productivity and farmers’ income. These nematodes induce and maintain highly metabolically active feeding sites in the roots of the host plants, which are their only source of nutrients throughout their life cycle. This long-term host-parasite interaction is characterized by sophisticated molecular and physiological dynamics. To ensure their survival, both the host and the parasite undergo a series of adaptations. The parasite depends on the host for the essential nutrients and energy required for its survival, growth, and reproduction. In response, the host activates immune responses and metabolic pathways to combat the infection. The metabolic complexity of these interactions has profound implications to the severity of infection and disease symptoms. However, the dietary requirements of nematodes remain largely unexplored. The present work aims to fill this knowledge gap by conducting metabolite profiling of infected host tissues in different host-pathogen systems, including tomato, rice, cucumber, carrots, and *Arabidopsis*. We will also profile the metabolites of the nematode parasite over time, covering all major parasitic stages. Our goal is to identify metabolites with consistent changes across various host plants. This study will pave the way for a comprehensive understanding of the metabolic dynamics in host-parasite interactions, potentially leading to more effective nematode management and control strategies.

## Elucidating the role of migpsy peptides in interaction between plants and root-knot nematodes

Lin, Ching-Jung^1^, H. Z. Yimer^2^, D. D. Luu^1^, A. C. Blundell^1^, M. F. Ercoli^1^, P. Vieira^3^, V. M. Williamson^1^, P. C. Ronald^1^ and S. Siddique^2^

^1^Dept. Plant Pathology, University of California Davis, Davis, CA, 95616

^2^Dept. Entomology and Nematology, University of California, Davis, California, One Shields Avenue, Davis, CA, 95616

^3^USDA-ARS Mycology and Nematology Genetic Diversity and Biology Laboratory, Beltsville, MD 20705

### Abstract

Plant-parasitic nematodes pose a severe threat to global food production. These parasites invade plant roots and establish permanent feeding sites, which serve as their sole source of nutrients. To manipulate host responses, they secrete effectors such as phytohormones or peptides that hijack the host’s cellular machinery. Plants produce a family of peptides called *PLANT PEPTIDE CONTAINING SULFATED TYROSINE* (PSY) that promote root growth via cell expansion and proliferation. Intriguingly, the bacterial pathogen *Xanthomonas oryzae* pv. *oryzae* also produces a PSY-like peptide called RaxX (required for activation of XA21 mediated immunity X), which contributes to bacterial virulence. Our previous research has identified a group of secreted peptides called MigPSYs in root-knot nematodes (*Meloidogyne spp.*) that resemble plant PSY peptides and stimulate root growth in Arabidopsis. We found that *MigPSY* transcript levels are highest during the early stages of infection in rice and tomato plants. Furthermore, down-regulating expression of *MigPSY* results in reduced root galling and egg production, suggesting that the MigPSYs serve as nematode virulence factors. To gain a better understanding of the roles of MigPSYs, I plan to characterize the mechanisms underlying their function and host perception in plants. This research is expected to provide valuable insights into the mechanism of nematode infection and may lead to the development of new methods for controlling plant-parasitic nematodes.

## Nimaxxa™ and kummat™: new biological nematicides for the control of plant parasitic nematodes

Ludwig, Scott, D. Narvaez and S. Sopher

UPL NA Inc, Research Triangle Park, NC 27709

### Abstract

Nimaxxa™ and KUMMAT™ are biological nematicides containing *Bacillus paralicheniformis* Strain CH2970, *B. paralicheniformis* Strain CH0273, and *B. subtilis* strain CH4000 being developed by UPL as seed treatment and in-soil applications for the control of plant parasitic nematodes. This combination of *Bacillus* species controls and protects crops against nematodes and root damage thought direct and indirect mode of action on plant parasitic nematodes. These three species cover a broad spectrum of conditions while providing multiple benefits to plants. They also increase the chances of a higher level of nematode control and a more consistent yield. *Bacillus paralicheniformis* CH2970 has high root colonization abilities. *Bacillus paralicheniformis* CH0273 is adapted to grow quickly at even low temperatures and acts as a plant biostimulant. *Bacillus subtilis* CH4000 produces key bioactive metabolites. In seed treatments the bacteria colonization occurs within the germinating root system and in soil applications the bacteria colonize the roots from the bulk soil. Using plant exudates present in the roots, the bacteria then colonize the root surface and grow together with the plant, providing protection against penetration by juveniles. When colonizing the root system, the bacteria produce nematicidal and antimicrobial compounds/metabolites that harm nematodes that approach the root. These active metabolites act directly against nematodes – both eggs and juveniles. Different plant growth-promoting compounds are also produced that help stimulate cell-wall expansion in the roots and promote root development, enhance plant growth, and ultimately increase plant biomass. Nimaxxa is being developed for seed treatment and KUMMAT for in-soil applications.

## A small cysteine-rich *Meloidogyne javanica* effector (MjCRSP) modulates plant immunity to promote nematode parasitism

Macharia, Teresia and L. Moleleki

Dept. Biochemistry, Genetics, and Microbiology, University of Pretoria South Africa, Forestry, Agriculture and Biotechnology Institute, University of Pretoria, South Africa

### Abstract

*Meloidogyne javanica* is an important root-knot nematode (RKN) species causing considerable economic losses globally. The specific mechanisms behind the interaction of these nematodes and their host plants remain poorly understood. Effectors are secreted molecules that mediate these intricate interactions. Nonetheless, the functions of different effectors in *M. javanica* infection have not received much attention thus far. In this study, using comparative genomic analysis, we identified a small cysteine-rich secreted protein encoded in *M. javanica* genome denoted as *Meloidogyne javanica* cysteine-rich secreted protein (hereafter, MjCRSP). MjCRSP is highly conserved in *Meloidogyne* species, has an N-terminus signal peptide and is predicted to be an extracellular protein. The full-length protein is 85 aa and of these, 12 aa (14.11%) are cysteine residues. The role and mechanism by which this effector contributes to plant parasitism was further elucidated. Towards this end, using fluorescence in situ hybridization we demonstrate that MjCRSP is expressed in the amphid secretory glands. Thereafter, we used the *Agrobacterium*-mediated transient expression system in *Nicotiana benthamiana*, to examine the ability of MjCRSP in suppression or induction of programmed cell death. We observed that this protein inhibits elicitin infestin1 (INF1)-triggered cell death and immune responses triggered by Flg22 peptide. Furthermore, subcellular localization showed that MjCRSP localizes in the cytoplasm. In conclusion, this study demonstrates that MjCRSP is a *M. javanica* effector protein that modulates plant immune responses during RKN-host interactions.

## Can altering soil pH be an effective control method for *Globodera pallida*, the pale cyst nematode?

Mann, Logan, L. M. Dandurand, and P. Hickman

University of Idaho, Dept. of Entomology, Plant Pathology and Nematology, Moscow, ID 83844

### Abstract

*Globodera pallida*, the pale cyst nematode (PCN), is a regulated pest in Idaho that has threatened Idaho’s potato industry since its discovery in 2006. The *G. pallida* infestation has the potential to cause up to 80% yield loss. Persistent in soil for decades, *G. pallida* eggs can remain viable as they await optimal hatching conditions. Currently, new research efforts for strategies of *G. pallida* eradication or control are of high importance, and understanding environmental factors is crucial for control. Much is being done to further knowledge of *G. pallida*, and to find proper methods of control and eradication. Since *G. pallida* lives in the soil ecosystem, they are affected by many soil characteristics, including soil texture, moisture, and temperature. Another abiotic soil factor that tends to impact ecosystem processes in soil is pH. This study aims to understand the relationship between soil pH and *G. pallida* tenacity. Infested fields in Idaho commonly have soils with neutral to alkaline pH. This study will test the impact of a range of pH (5.5, 6.0, 6.5, 7.0, and 7.5) on egg viability and hatching *in vitro and* in greenhouse trials. The soil that will be used has a pH of 5.5 and will be raised using two methods to negate any other impacts of each method used. Quicklime (CaO) and phosphate buffers will be used to raise pH in both *in vitro* and in greenhouse trials. *In vitro* assays will occur over a 3-week period, whereas greenhouse infection assays will occur over a 6-week period. The greenhouse trials will be analyzed by acid fuchsin staining at 6 weeks of growth to check infection. For these assays, ‘Russet Burbank’ will be the cultivar tested as it is one of the most prevalent potato cultivars produced. When comparing all collected data, the goal of this study will be to analyze data and find the relationship between soil pH and *G. pallida* viability and hatch. Ultimately, this study hopes to determine the impact of soil pH on *G. pallida* as this may help to enhance control measures.

## Integrating morphological and molecular approaches for nematode identification: a case study of coffee nematodes in Kenya

Maosa, Joseph^1^, D. Gitonga^2^, Z. Githinji^1^, H. T. Nguyen^3^, M. Couvreur^1^, L. Cortada^4^, C. Oduori^5^, D. Coyne^1,5^ and W. Bert^1^

^1^Dept. Biology, Ghent University, Ghent, Belgium

^2^Dept. Entomology and Nematology, Fort Lauderdale Research and Education Center, University of Florida, Davie, FL 33314

^3^Institute of Ecology and Biological Resources, Vietnam Academy of Sciences and Technology, Hanoi, Vietnam

^4^VIB-International Plant Biotechnology Outreach (IPBO), Technology Park, Ghent, Belgium

^5^International Institute of Tropical Agriculture, Nairobi, Kenya

### Abstract

As the landscape of biological classification constantly evolves, a growing prevalence in the use of sequence-based identification, or molecular barcoding, is being observed, while a concomitant decline in morphology-based taxonomy is also evident. However, a coherent link between conventional taxonomic units, such as species, and their respective DNA sequences remains imperative. Overcoming this challenge involves navigating numerous obstacles, such as minimal inter-species variations, substantial intra-species diversity, the ongoing quest for the best barcode, and the accurate correlation of DNA sequences with genuine species identities. Using nematodes from coffee sourced in Kenya, we underscore the inherent challenges and opportunities in harmonizing traditional morphological techniques with molecular methods for nematode species identification. Initially, morphologically vouchered individual specimens were sequenced, thus enhancing accuracy in public databases and avoiding potential misinterpretations. Through the analysis of data collected from 158 soil and 156 root samples, we offer a compelling demonstration of the limited exploration of nematode diversity within a particularly key crop and the complexities associated with identification. Of particular significance, we delve deep into the characterization of *Trophotylenchulus obscurus*, an understudied nematode of notable importance, and present its first 18S, D2-D3 of 28S rRNA, and ITS sequences. Furthermore, we identified the most common species morphologically and molecularly, such as *Meloidogyne africana, M. hapla, M. javanica, Helicotylenchus* spp., *Rotylenchulus macrosoma*, *Pratylenchus goodeyi,* and a putative new *Pratylenchus* species. An overview is also provided of the prevalence of all nematode genera recovered, including *Aphelenchoides, Hemicycliophora, Meloidogyne, Paratylenchus, Pratylenchus, Rotylenchulus, Scutellonema, Trophotylenchulus, Trichodorus, Tylenchulus,* and *Xiphinema.* In conclusion, our research illuminates crucial gaps in nematode diversity studies within Kenyan coffee sector, and our findings strongly advocate for the implementation of integrative approaches for nematode identification.

## Soybean cyst nematode virulence in Iowa: selection marches on steadily

McCarville, Michael T.^1^, C. C. Marett^2^, M. P. Mullaney^2^, G. D. Gebhart^2^ and G. L. Tylka^2^

^1^BASF, Omaha, NE

^2^Dept. Plant Pathology, Entomology and Microbiology, Iowa State University, Ames, IA 50011

### Abstract

Management of the soybean cyst nematode (SCN), *Heterodera glycines*, in the state of Iowa has relied on growing soybean varieties derived largely from a single source of SCN resistance, namely PI 88788. Previously published results of analyses of Iowa State University SCN-resistant variety trial data from 2001 to 2015 documented that prolonged, widespread use soybean varieties with PI 88788 SCN resistance led to an increase in SCN virulence on PI 88788 with corresponding negative effects on soybean yield. Results from similar experiments conducted from 2016 through 2020 were added to the dataset, and the analyses were repeated to determine if the trends initially reported continued and if the conclusions remained the same. Results of the analyses of the expanded data revealed that major trends detected from 2001 to 2015 continued between 2016 to 2020. Specifically, virulence on PI 88788 among SCN populations in Iowa increased at a rate nearly identical to that predicted from the 2001 to 2015 data, and the elevated virulence resulted in increasing yield loss on varieties with PI 88788 SCN resistance. Results of the linear regression analyses of the expanded data predict that by the end of this decade, cultivars with SCN resistance from PI 88788 will provide only about 40% control of SCN populations in Iowa resulting in average yield losses of approximately 10 bu/acre or 13% of soybean yield potential. These same trends do not appear in the expanded data for soybean varieties with Peking SCN resistance. There was no significant increase in the average SCN reproduction on Peking-resistant varieties among the data, but there was an increase in the number of experimental locations with SCN populations with elevated virulence (female indices >10%) on Peking between 2016 to 2020. These results further illustrate that moving forward diverse management strategies are necessary to combat SCN and alter the current trajectory of SCN management for declining efficacy and increasing yield loss.

## Development of microbial bionematicides: an industry perspective

Medina, Karla

Certis Biologicals, Columbia, MD 21046

### Abstract

A new horizon for bionematicides is occurring across the globe. The management of plant-parasitic nematodes is best when the approach integrates several tools and practices to suppress and manage populations below levels to stave off reductions in yield. The selection for microbial bionematicide development differs slightly from traditional synthetic nematicides in that microbial commercial products can get to market faster and are less expensive regarding regulatory hurdles than chemical products’ regulatory path. From an industry perspective, developing bionematicides involves the first step, sourcing the strain or selecting viable organisms for scale-up. This goal is to present and outline the process(es) regarding R&D, including screening, selection, and formulation, the manufacturing process to scale up, and the steps to evaluate in field, regulatory requirements, and developing the commercialization and support of the product once launched. Understanding the process for developing nematicides involves both basic and applied research. Investing and learning more on nematode biology and behavior can lead to discovering better and new products.

## Unraveling the roles of neuropeptides in chemosensation of the root-knot nematode *Meloidogyne javanica*

Mo, Chenmi^1^ and L. Zhang^1,2^

^1^Dept. Botany and Plant Pathology, Purdue University, West Lafayette, IN 47907

^2^Dept. Entomology, Purdue University, West Lafayette, IN 47907

### Abstract

Identification of novel drug targets in plant-parasitic nematodes (PPNs) is imperative due to the loss of traditional nematicides and a lack of replacements. Chemosensation, which is pivotal for PPNs in locating host roots, has become a focus in nematode behavioral research. However, its underlying molecular basis is still indistinct in such a diverse group of PPNs. To characterize genes participating in chemosensation in the Javanese root-knot nematode *Meloidogyne javanica*, RNA-sequencing of the second-stage juveniles (J2s) treated with tomato root exudate (TRE) for 1 hour and 6 hours was performed. Genes related to chemosensation in *M. javanica* mainly responded to TRE treatment at 1 hour. Moreover, Gene Ontology (GO) analysis underscored the significance of the neuropeptide-G protein-coupled receptor signaling pathway. Consequently, the repertoire of putative neuropeptides in *M. javanica*, including FMRFamide-like peptides (FLPs), insulin-like peptides (ILPs), and neuropeptide-like peptides (NLPs), were outlined based on homology analysis. The gene *Mjflp-14a* harboring two neuropeptides was significantly up-regulated at 1 hour TRE treatment. Through peptide synthesis and J2 treatment, one of the two neuropeptides (MjFLP-14-2) was proved to influence the J2 chemotaxis towards tomato root tips. Overall, our study reinforces the potential of nematode neuropeptides as novel targets and tools for root-knot nematode control.

## A survey of plant-parasitic nematodes on alfalfa with an emphasis on *Ditylenchus dipsaci* in Virginia

Mony, Fatima Tuz Zohora and J. D. Eisenback

School of Plant and Environmental Science, Virginia Tech, Blacksburg, VA 24061

### Abstract

Alfalfa, a crucial crop for the Virginia dairy industry, faces potential threats from plant-parasitic nematodes, including *Ditylenchus* spp. Despite the significant production of alfalfa to 270,000 tons in the U.S. in 2022, the impact of these nematodes in Virginia remains unclear due to the lack of a comprehensive survey since 1948. These nematodes can cause the plants to grow poorly, thereby reducing the amount of biomass produced. They may also reduce the number of years that this perennial crop remains in the field. This study investigated the presence and impact of *Ditylenchus* spp. and other nematodes on alfalfa production across farms in Virginia. In our initial survey of Pulaski County, *Ditylenchus* spp. was identified in soil and stem samples from two fields, while a variety of other plant-parasitic nematodes were present in all fields. Dagger, (*Xiphinema* spp.), lance (*Hoplolaimus* spp.), and stunt (*Tylenchorhynchus* spp.) were found in all fields except one. Spiral (*Helicotylenchus* spp.) was found in all fields except two. Lesion (*Pratylenchus* spp.), ring (*Criconemella* spp.), and pin (*Paratylenchus* spp.) were found less often. Cyst nematode (*Heterodera* spp.) was found in one sample, but it was not identified to species. Since the alfalfa cyst nematode, *H. medicaginis*, was found in Utah and Kansas, we are planning to sample additional specimens so that an accurate species identification can be made. The complete survey will sample all the major alfalfa-growing counties in Virginia. We will evaluate the key nematodes in alfalfa fields and determine the role that they may play in the production of alfalfa in Virginia.

## Advancing soybean cyst nematode management through evaluation of chemical and biological seed treatments in Ohio

Moore, Jenna, S. Mondal, T. Ralston and H. D. Lopez-Nicora

The Ohio State University, Dept. Plant Pathology, Columbus, OH 43210

### Abstract

Soybean cyst nematode (SCN; *Heterodera glycines* Ichinohe) poses a significant threat to agricultural productivity, causing extensive damage to soybean and leading to substantial economic losses. In the past few decades, there has been a noticeable decrease in the efficacy of genetic resistance. Effective management strategies are crucial to mitigate these impacts and ensure sustainable crop production. Among the various approaches available, chemical and biological seed treatments have emerged as promising routes for nematode control, with many producers spending a great portion of expenses on seed treatment packages. However, the control offered by these treatments (suppressing nematode population and minimizing crop damage) is not well documented for the Midwest-Atlantic region. Additionally, concerns regarding environmental impact, non-target effects, and the development of resistance highlight the necessity for continuous evaluation and optimization of chemical control measures. In contrast, biological seed treatments leverage the antagonistic interaction between beneficial microorganisms and plant parasitic nematodes to achieve sustainable nematode management. Biocontrol agents have the potential to effectively suppress nematode populations while preserving ecological balance and soil health. Moreover, biological treatments could promote plant growth and reduce reliance on chemical inputs. From 2022 and 2023, seed treatment evaluations took place at Western (Clark Co., OH) and North Central (Sandusky Co., OH) Agricultural Research Stations. These trials, employing a randomized complete block design with four to six replications, assessed chemical (pydiflumetofen, fluopyram, thiamethoxam + mefenoxam + fludioxonil, inpyrfluxam + mandestrobin + fludioxonil + mefenoxam, clothianidin) and biological (*Bacillus amyloliquefaciens* strains MBI600 and PTA-4838, and *Burkholderia spp.* strain A396) seed treatments. SCN populations were obtained at planting, 45 days after planting, and at harvest and SCN reproduction factor (RF = SCN population at harvest / SCN population at planting) was calculated. Plant population and yield data were recorded. Data from each trial was analyzed separately using analysis of variance (ANOVA). Our findings indicate that none of the treatments exhibited a statistically significant (*P* > 0.05) suppression of the final population of SCN compared to the untreated control. An average RF of 2.28 was observed for all trials, with a range of 0.22 to 12.81. Furthermore, we did not observe any significant differences (*P* > 0.05) in yield or plant stand among the various treatments. These findings highlight the need for an integrated SCN management strategy rather than sole reliance on chemical or biological seed treatments.

## High-throughput qPCR screening of export certification samples reveals that *Meloidogyne floridensis*, the peach root-knot nematode, is rare in Florida ornamental plant nurseries

Moore, Matthew R.^1^, L. A. Combee^1^, C. G. Roberts^1^, R. Xue^2^, S. J. Vau^2^ and J. A. Brito^2^

^1^Molecular Diagnostics Laboratory,

^2^Nematology Section, Florida Department of Agriculture and Consumer Services, Division of Plant Industry, Gainesville, FL 32608

### Abstract

The peach root-knot nematode, *Meloidogyne floridensis*, is a plant pathogen of growing regulatory significance due to its ability to infect RKN resistant *Prunus* cultivars. *Meloidogyne floridensis* is quarantined from at least one California county, necessitating the State of Florida to certify plant material free of this nematode when it is destined for export to this area. Rapid and reliable species-level identification of root-knot nematodes is a critical aspect of regulatory nematology. A triplex qPCR assay that identifies *M. floridensis* was deployed to understand the scope of this issue for Florida ornamental nurseries. Export certification samples dating back to 2019 were screened for *M. floridensis* to generate a historical perspective on the problem. Over 2,000 DNA extractions have been assayed and *M. floridensis* has not been detected in any ornamental nursery material from this period. Other aspects of the qPCR assay’s performance are described, including its ability to identify *M. hispanica*.

## Syngenta^®^ seedcare™ introduces TYMIRIUM^®^ technology as a new nematode solution for us cotton

Moore, Scott R.^1^ and S. Morsello^2^

^1^Syngenta Crop Protection, Monroe, LA 71201

^2^Syngenta Crop Protection, Greensboro, NC 27419

### Abstract

Plant parasitic nematodes are a major yield limiting factor in US cotton production, particularly the root-knot nematode (*Meloidogyne incognita*) and the reniform nematode (*Rotylenchulus reniformis*). TYMIRIUM^®^ technology is a new seed treatment developed by Syngenta Crop Protection for the control of plant-parasitic nematodes prevalent in cotton as well as yield robbing diseases, like Texas root rot (*Phymatotrichopsis omnivora*). An overview of US-focused research and development of TYMIRIUM^®^ technology and the benefits of its use in cotton will be presented. When registered, TYMIRIUM^®^ technology is anticipated to provide cotton growers a strong tool for both nematode and Texas root rot protection.

## Integrated nematode management on watermelons in honduras

Moreira, David^1^, J. Desaeger^1^ and C. Avellaneda^2^

^1^Dept. Entomology and Nematology, Gulf Coast Research and Education Center, 14625 CR 672, Wimauma, FL 33598

^2^Agronomy Department, Zamorano University, Francisco Morazan, Honduras

### Abstract

Honduras, a nation heavily reliant on agriculture, faces significant challenges posed by tropical plant-parasitic nematodes. With limited knowledge of their impact on various crops, particularly watermelon—an essential export generating over $17 million annually—there is a pressing need to evaluate effective management strategies. This study was conducted on a commercial watermelon field near Zamorano University in 2023. Different nematode management options include a cover crop, locally available soil amendments and chemical and biological nematicides. The field measured about 1 ha and was split in two with one half planted with a cover crop (sunn hemp ‘*Crotalaria juncea*’) three months before planting and the other half left fallow. After mowing and incorporating the cover crops, the following treatments were applied: fresh chicken litter (14.8 T/Ha), sugarcane molasses (14,031 L/Ha), sugarcane cachaca (14.8T/Ha), Nimitz ‘fluensulfone’ (2L/Ha), Agrocelhone NE ‘35% chloropicrin and 65% 1,3-dichloropropene’ (70L/Ha), and Pazam + Trichozam’ *Purpureocillium lilicanum* and *Trichoderma harzianium*’ (8L/Ha). The most common plant-parasitic nematodes (PPN) found were reniform (*Rotylenchulus* spp.), spiral (*Helicotylenchus* spp.), root-knot (*Meloidogyne* spp.) and lesion nematodes (*Pratylenchus* spp.). Root-knot nematode pressure was low and no differences in root gall rating or soil populations were found at the end of the watermelon crop. Other PPN also did not show significant differences among treatments. Watermelon growth and yield also was not significantly different among treatments, with the fumigant treatment Agrocelhone D demonstrating the highest fruit weights, while Nimitz exhibited the highest crop vigor. A second identical trial is currently ongoing in the same field, and treatments were applied to the same plots as in 2023. Our study is a first attempt to help implement practical and effective nematode management solutions to growers in regions where PPN are still largely unknown and not studied. Particularly in regions like Honduras, where agriculture is a cornerstone of the economy, sustainable nematode management practices are a valuable tool in bolstering agricultural resilience and livelihoods.

## Active plant nematologists (APN): cultivating collaboration, overcoming challenges, and shaping the future of nematology

Moreira, David^1^, D. Pires^2,3^, R. Collett^4^, V. Orlando^5^, E. Darling^6^ and J. Coburn^7^

^1^Dept. Entomology and Nematology, Gulf Coast Research and Education Center, University of Florida, Wimauma, FL 33598

^2^Instituto Nacional de Investigação Agrária e Veterinária, Av. da República, Quinta do Marquês, Edifício Florestal, 2780-159 Oeiras, Portugal

^3^Mediterranean Institute for Agriculture, Environment and Development & Global Change and Sustainability Institute (CHANGE), Institute for Advanced Studies and Research, University of Évora, Pólo da Mitra, 7006-554 Évora, Portugal

^4^Unit for Environmental Sciences and Management, North-West University, Potchefstroom 2520, South Africa

^5^Fera Science Ltd., Biotech Campus, Sand Hutton, York, YO41 1LZ United Kingdom

^6^Dept. Entomology, University of California, Riverside, Parlier, CA 93648

^7^Corteva Agriscience, Myakka City, FL 33594

### Abstract

Active Plant Nematologists (APN) is a global scientific community established during the 2020 coronavirus pandemic to address the disruption of physical conferences and meetings in the field of Plant Nematology. Committed to fostering collaborations among nematology professionals and enthusiasts, APN provides a safe space for individuals worldwide, irrespective of their position, location, or background, emphasizing the vital role of nematodes in soil health and crop protection. Initially founded with a SLACK workspace, APN has grown to include 564 registered members globally, with diverse channels covering topics such as nematode taxonomy, phylogenetics, student and professional opportunities, and more. Expanding its presence across various social media platforms, APN has successfully co-organized two virtual seminars, generously sponsored by ADAMA US, attracting over 500 participants from numerous countries. These events featured parallel student contests, including a 5-minute thesis competition and a poster competition. In a recent initiative, APN collaborated with the Young Nematologists Network of the European Society of Nematologists to host the second edition of the Virtual Nematology Conference. The group is continuously exploring alternative communication methods within Nematology and engaging a broader audience. Notably, APN conducted a comprehensive survey on “the future of nematology,” seeking insights from nematologists worldwide about their motivations and aspirations within the field. This survey provided valuable data to assess the current state of nematology and identify challenges faced by the newer generation of nematologists. The results of this groundbreaking survey will be presented in a dedicated 2-hour symposium. The first hour will feature 4–5 short talks by academic and industry representatives, providing diverse perspectives on the survey findings. The second hour will host a panel Q&A with the speakers, combining prepared template questions and audience queries to facilitate in-depth discussions. This symposium aims to offer a comprehensive look into the challenges that nematologists need to focus on in the future, fostering the growth of the field and providing expanded career options for professionals. Join us as we delve into the essence of nematology, share insights, and collectively shape its promising future.

## Why I hate Tobrilidae

Mullin, Peter

University of Nebraska-Lincoln, Dept. Plant Pathology, Lincoln, NE 68583

### Abstract

If you were looking for the “typical freshwater nematode”, you wouldn’t go far wrong in selecting a member of the family Tobrilidae. Found on all continents and notable for their ability to survive in a range of challenging habitats – from pH and salinity extremes to anoxic sediments – these remarkable creatures would seem to be ideal subjects of study for nematode taxonomists, ecologists, and physiologists. Yet very little detailed research has been conducted on members of this group outside of Lake Baikal, which seems to be a center of diversity for one main branch of the family. In North America, a likely region of origin for another major lineage, almost nothing is known about the distribution, diversity, or life history of tobrilid nematodes. In part, this is due to a lack of interest, with research efforts being focused on more economically important taxa. But study is also hamstrung by the challenges and frustrations that arise when collecting, examining, and attempting to identify members of this group. Living animals are vigorously active and difficult to manipulate and morphological characters are confusing and inconsistent, varying with life stage, maturity and even the position of a mounted specimen. Identification using DNA sequences is hampered by a lack of reference sequences, inaccuracies in the naming of the sequences that do exist, and the apparent ability of these nematodes to hybridize in nature. Insights gained during recent exploration of several freshwater habitats in Nebraska and study of the members of Tobrilidae recovered from those habitats definitely underscore these difficulties, but also provide some glimpses of better understanding and indicate some areas for future work.

## Understanding the interplay and influence of abiotic and biotic stress on plant-parasitic nematodes: perspectives and future directions from an ecological standpoint

Mundim, Fabiane

Utah State University, Dept. of Biology, Logan, UT, 84322

### Abstract

Environmental change, whether driven by natural phenomena or human activities, disrupts soil conditions and alters the delicate ecological balance within host plant and nematode interactions. These changes have profound implications for both free-living and plant-parasitic nematodes. For instance, while plant-parasitic nematodes generally have their infection distribution profoundly affected by soil temperature, free-leaving nematodes’ responses to soil stress vary among trophic groups, with those having shorter generation times and high fecundity showing a positive response while those with longer generation times and lower fecundity being negatively affected. Collectively, changes in soil physical properties and the presence of herbivores and microbial pathogens directly impact plant growth, development, and defenses against antagonistic organisms, ultimately altering their susceptibility to parasitic nematode infestation. As host plants serve as breeding grounds for some parasitic nematodes, abiotic and biotic changes can lead to direct and indirect variations in plant-parasite relationships, including modifications in host immune responses, parasite virulence, and interaction specificity. These changes have the potential to alter the evolutionary trajectories of both hosts and parasites, potentially facilitating speciation. Furthermore, environmental changes affect other trophic groups, including predators, hyperparasites, and symbionts, which in turn influence plant-parasitic nematodes. Therefore, the increasing focus on the influence of biotic and abiotic stressors on interactions between plants and nematodes is critical, especially in the context of global changes. Plant-parasitic nematodes pose significant biotic stress on crops globally, with over 4000 species threatening plant health and food security. Management and control of parasitic nematodes not only plays a crucial role in agricultural systems but also impacts the biodiversity of natural environments. As such, integrating concepts from nematology and evolutionary ecology enhances our ability to predict disease outbreaks, develop improved crop plants, design effective control measures, and comprehend soil community dynamics, local adaptation, speciation, and genetic diversity. This interdisciplinary approach will pave the way for addressing critical agroecological issues with increased problem-solving capabilities. Ultimately, by formally bridging the fields of nematology and ecology we can enhance their theoretical frameworks and develop more precise and efficient approaches to manage nematode-related challenges in both agricultural systems and natural environments.

## Description and molecular characterization of a new dorylaimid nematode (nematoda: dorylaimidae) from Korea

Mwamula, Abraham Okki^1^, S. M. Lee^2^, Y. H. Jung^2^, Y. S. Kim^1^ and D. W. Lee^1,3^

^1^Research Institute of Invertebrate Vector, Kyungpook National University, Sangju 37224, Republic of Korea

^2^SM Biovision Co., Jinju, 52849, Republic of Korea

^3^Dept. Entomology, Kyungpook National University, Sangju, 37224, Republic of Korea

### Abstract

During an ecological survey in the pine forest ecosystem in Korea, an undescribed species belonging to *Mesodorylaimus* was recovered from the bark and cambium layer of dead black pine (*Pinus thunbergii*) tree stands. The population is herein described using integrative taxonomy, considering both morphological and molecular phylogenetic analyses of the 18S- and 28S-rRNA. The new *Mesodorylaimus* species is characterized by having a medium sized body *ca* 1.50–1.89 mm long, lip region angular and offset by a depression, a relatively long odontostyle (17.0–19.5 *μm*), vulva opening transverse slit, positioned slightly posteriorly (V = 50–55), *pars refringens vaginae* with two elongate drop-shaped to spindle-shaped sclerotizations, intestine-prerectum junction with a long anteriorly directed conical or tongue-like projection, a relatively long female tail (115–187 *μm*; c = 10.0–13.6; c’ = 4.9–7.0), spicules 49.0–57.0 *μm* long, and the regularly spaced 7–8 ventromedian supplements. It is closest to *M. subtilis*, especially in having similar body length and number of ventromedian supplements but can be differentiated from *M. subtilis* by the longer odontostyle, tongue-like projection and longer spicules. The phylogenies based on the 18S- and 28S-rRNA sequences showed a well-supported sister relation of the new *Mesodorylaimus* species with *M. subtilis, M. japonicus, M. bastiani, M. pseudobastiani, Calcaridorylaimus castaneae, C. heynsi,* and other member species of the group.

## The population structure of *Bursaphelenchus xylophilus* and insect-associated nematodes occurring in natural pinewood nematode-infested dead pine tree stands in island areas

Mwamula Abraham Okki^1^, S. M. Lee^2^, Y. H. Jung^2^, Y. S. Kim^1^ and D. W. Lee^1,3^

^1^Research Institute of Invertebrate Vector, Kyungpook National University, Sangju 37224, Republic of Korea

^2^SM Biovision Co., Jinju, 52849, Republic of Korea

^3^Department of Entomology, Kyungpook National University, Sangju, 37224, Republic of Korea

### Abstract

The pine wood nematode (PWN), *Bursaphelenchus xylophilus*, is a well-known devastating pathogen of economic importance causing pine wilt disease (PWD) on susceptible pine tree species in Korea and other countries. This disease involves complicated interactions between the host pine species, the nematode, vector beetle, and fungi in dead host trees. Insect-associated, and other free living nematodes also have complex trophic associations with fungi and other organisms in dead trees. In this study, the structure and population density of *B. xylophilus* in relation to insect-parasitic and/or insect-associated nematode species were investigated in dead black pine tree stands (*Pinus thunbergii*) on an island that is naturally infested by PWN. Nematodes were recovered from 123 dead pine tree samples taken at diameter at breast height level. *Bursaphelenchus xylophilus* was detected in 82.9% of the sampled trees. The insect-associated nematodes, *Diplogasteroides* sp. and *Parasitorhabditis terebranus* showed detection rates of 50.4% and 18.7%, respectively. Coexistence of two or more species of nematodes was recorded in 51.2% of the tree samples. However, in all cases of coexistence, there was a negative correlation between PWN population and insect-associated nematode groups, especially *Diplogasteroides* sp.

## Management of wood-boring beetles by injection of *Heterorhabditis indica* in *Persea americana* and *Theobroma cacao*

Myers, Roxana and C. Mello

USDA-ARS, Daniel K. Inouye U.S. Pacific Basin Agricultural Research Center, Hilo, HI 96720

### Abstract

Invasive insect pests are increasingly becoming a problem to the agricultural industry and delicate ecosystem of the Hawaiian Islands. A recent introduction, *Acalolepta aesthetica*, the Queensland long-horned beetle (QLB) is a large polyphagous wood-boring cerambycid that attacks a wide range of tropical tree species. Preliminary trials using a native isolate of *Heterorhabditis indica* demonstrated that the nematode could navigate galleries created by QLB larvae, infect, and cause mortality within trunks and branches of infested trees. A field trial was initiated to manage this pest in avocado, *Persea americana*, and cacao, *Theobroma cacao*, orchards by injecting the nematodes in an aqueous suspension into openings in the trunk caused by QLB larval feeding and tunneling. Inoculum rates ranged from 20,000 to 240,000 IJs/tree, depending on the levels of QLB infestation and the amount of the aqueous nematode suspension the galleries could hold. Initial evaluation of the treatment’s efficacy was established by observing for new signs of larval activity such as fresh frass, wood shavings, and oozing sap one month after application of *H. indica*. Treatments were conducted monthly in trees that continued to show signs of QLB larval feeding. In avocado, four weeks after the initial application 33% of the nematode treated trees showed no signs of recent QLB activity. This increased to 75% one month after the second application. Following the third treatment of *H. indica,* 85% of trees no longer appeared to have active infestations. In the cacao orchard, where the infestation was more severe prior to starting the nematode applications, 50% of the trees no longer had signs of QLB larval feeding after the first treatment. However, signs were still observed on the remaining infested trees after two additional treatments of *H. indica* possibility due to the large number of QLB larvae initially colonizing those trees. Due to the cryptic habitat of this pest, it cannot be confirmed that the absence of signs of QLB activity is entirely the result of mortality caused by *H. indica* without felling and dismembering the trees. Additional data will be collected on the number of QLB adult emergence holes that occur on nematode inoculated trees when adult beetles exit their host trees during mating season.

## Integrated nematode management strategies in FL small fruit and vegetable crops: how the integration occurs

Noling, Joseph^1^ and J. Desaeger^2^

^1^Citrus Research & Education Center, IFAS, University of Florida, Lake Alfred, FL 33850

^2^Gulf Coast Research and Education Center, Wimauma, FL 33598

### Abstract

Effective, economical, ecologically based, integrated management of nematodes continues to be a key component of sustainable food and fiber production. Given the growing demand for more sustainable management strategies, descriptions of what constitutes an integrated nematode management (INM) system, including those needed to manage other prominent soilborne pests, weeds, and diseases will be discussed. Integrated strategies now include various biological and chemical pest control products, inclusion of genetic resistance to nematodes and disease where possible, and cultural practices, all of which require a greater knowledge of specific nematode biology, particularly with multiple pest species, to achieve satisfactory results. This requires timely monitoring; proper identification and assessments of population densities of plant pathogenic nematodes present within the field. The focus of this presentation will be to describe the importance of INM components, and why farmers have adopted these and not others to resolve their production problems. Since nematodes don’t ever operate alone, farmers have to choose or sacrifice preferred resistant varieties and other production practices based on prioritized needs to address the entire pest complex present. We will address how the true integration of INM (adoption by growers) involve overcoming certain barriers or constraints, i.e., costs and profitability (cost benefit analysis), risks (such as preplant decisions- vs -the availability of efficacious post plant remediations), requirements for new equipment, significant changes in the production system (new equipment, changes in bed geometry, rotations, crop diversification). The diversity of soilborne pest and disease problems within a field usually demands a site-specific mix of management practices to be used. Some management practices require an assessment of the depth distribution of nematodes within the soil profile, which will determine the site-specific prescription for nematode treatment. Successful crop production in an INM system also relies heavily on use of certified pest-free plants to avoid unresolvable problems after planting. Although genetic resistance is a preferred strategy for nematode management, resistance genes are not always available for a given crop and nematode species. Cover cropping is another important INM consideration. Inclusion of Sunn hemp as a warm season cover crop in Florida is extensively used as a legume to add nutrients and organic matter and to suppress weeds and a variety of plant parasitic nematodes between crop cycles. However, the list of suitable alternative cover crops capable of reducing a broad range of nematode species is still limited. In many crops, nematodes cannot be effectively managed without simultaneous consideration of weed management. Within INM, the causes for treatment inconsistency can be diverse and is in need of further clarification to improve grower adoption and utility. For example, with such limited horizontal spreading, making use of the drip irrigation system is hard to include into an overall integrated nematode management plan when water movement within the soil profile is so fast and so spatially limited. Protocols to maximize drip chemigation of any chemical or biological product to be more locally and site specifically developed.

## Draft genome of the hop cyst nematode, *Heterodera humuli* from the pacific northwest

Núñez-Rodriguez, Lester A.^1,2^, C.L Wram^3^, C. Hesse^4^ and I. A. Zasada^2^

^1^Dept. Botany and Plant Pathology, Oregon State University, Corvallis, OR 97331

^2^USDA-ARS Horticultural Crops Disease and Pest Management Research Unit, Corvallis, OR 97331

^3^USDA-ARS Mycology and Nematology Genetic Diversity and Biology Unit, Beltsville, MD 20705

^4^USDA-ARS Horticultural Crops Production and Genetic Improvement Research Unit, Corvallis, OR 97331

### Abstract

The hop cyst nematode, *Heterodera humuli*, is the most common plant-parasitic nematode found in hopyards. However, limited information is available about *H. humuli*. The aim of this study was to generate a high-quality reference genome of *H. humuli* and implement a genome skimming strategy to analyze the potential presence of endosymbionts. To achieve this, 10,200 nematodes (eggs and second-stage juveniles) were sent to the Roy J. Carver Biotechnology Center, University of Illinois at Urbana-Champaigne, Illinois for DNA extraction. The PacBio Ultra-Low DNA input method was used for sequencing using the PacBio Sequel IIe system. Then, PacBio HiFi reads were filtered with GNU Awk v4.0.2 and reads less than 3 kb were removed. The meta-assembly was performed with Hifiasm v.0.16.1 with default parameters. The GC content and read coverage were used to visualize the *de novo* assembled contigs with Blobtools v.1.0. Contigs were given a phylum identity based on BLAST similarity (E-value < 10e^−25^) to sequences in the NCBI “nt” database or other plant-parasitic nematode genomes. Contigs identified as Nematoda or with no assign identity (no-hit) that have a coverage of ≥ 10x were used to re-assemble the *H. humuli* genome using Hifiasm v.0.16.1. Blobtools v.1.0. was used again to visualize the re-assembly following the process described above. In a separate analysis, the bacterial endosymbiont, *Wolbahcia* was found in the meta-assembly and coded as *wH.hum*. Statistics and completeness of both genomes were assessed using QUAST and BUSCO v.5.5.0, respectively. The genome of *H. humulis* and *Wolbachia* were annotated with BRAKER3 v 3.0.3 and the prokaryotic genome annotation pipeline, respectively. To perform phylogenetic analysis, the partial *cox1* gene of *H. humuli* and the 16S gene *Wolbachia* were retrieved using usearch11 and ConEst16S, respectively. The assembled genome size of *H. humuli* is 90,806,450 bp in a total of 1,487 contigs with an average 112X coverage. The genome of *wH.hum* was found in a single contig of 1,051,007 bp. The BUSCO completeness of the genomes of *H. humuli* and *wH.hum* was 43.9 % and 98.1 %, respectively. A total of 15,428 coding genes were predicted for *H. humuli.* A total of 987 CDS were predicted for *wH.hum*. The phylogenetic analysis placed the *cox1* sequence from our genome of *H. humuli* with other sequences of *H. humuli*. In the case of *wH.hum*, the 16S sequence was placed in the group L, which contains the *Wolbachia* strains associated with plant-parasitic nematodes. To our knowledge, this is the first high-quality reference genome of *Heterodera humuli*. Additionally, this is the first report of *Wolbachia* associated with a cyst-forming nematode.

## *Purpureocillium lilacinum* strain PL11 (NemaClean™ 10% WP): a bionematicide for the control of plant-parasitic nematodes in fruit and vegetable production

Oliveira, Clemen and K. Medina

Certis Biologicals, Columbia, MD 21046

### Abstract

NemaClean™ 10% WP, which contains *P. lilacinum* strain PL11, is a new bionematicide launched in 2024 by Certis Biologicals and is now available to growers. This fungus is a biocontrol agent that occurs naturally in the rhizospheres of many crops and is one of the most used biological nematicides commercially available globally. Increased demand for sustainable food production and benefits from using biological products in integrated nematode management have been observed in many countries. In the United States, few bionematicide alternatives are available to growers and researchers, and therefore efficacy evaluations of bionematicides against plant-parasitic nematodes are important to better understand their role in an integrated nematode program. This study focuses on learning the fit of *P. lilacinum* strain PL11 on fruit and vegetable production and its efficacy against plant-parasitic nematodes. We collaborated with private contractors and University faculty across the US to perform efficacy data from greenhouse and field trials. Over the past growing seasons, *P. lilacinum* strain PL11 demonstrated to be a promising bionematicide for reducing nematode population densities and increasing yield. The efficacy of *P. lilacinum* strain PL11 was observed when used as a standalone and in a program with other management tools. The data mainly focused on root-knot nematode, but efficacy was also observed against other genera of plant-parasitic nematodes. Generating field and lab data on *P. lilacinum* strain PL11 will continue to expand into other crop systems and nematodes across different regions of the United States.

## Potato cyst nematodes (PCN) *Globodera pallida* and *G. Rostochiensis* in Africa: current status and sources of host resistance

Onditi, John O. ^1,2^ and J. L. Whitworth^1^

^1^USDA-ARS, Aberdeen, ID 83210

^2^Kenya Agricultural and Livestock Research Organization (KALRO), Food Crops Research Institute (FCRI), Kitale, Kenya

### Abstract

Potato (*Solanum tuberosum* L.) is one of the major crops in Africa with the potential of improving food and nutritional security. Potato cyst nematodes (PCN), *Globodera pallida* and *G. rostochiensis* have more recently been reported as a new pest challenging production of the crop in Africa. Knowledge of prevalence and distribution of the pest and the available sources of resistance can provide an immediate option of PCN control that can be recommended to the farmers. The aim of the study was to analyze the status of prevalence and distribution of the pest in the region and identify sources of host resistance in existing cultivars which can be used in its control. The review revealed *G. rostochiensis* as the most widespread PCN species reported in eight African countries as compared to *G. pallida* only found in four countries. *G. rostochiensis* Ro1/4 and *G. pallida* Pa/2/3 were the only PCN pathotypes that have been reported in Africa. Multiple PCN resistances to the most predominant PCN species and pathotypes were found in each of the PCN infested countries particularly among cultivars originally sourced from Europe. This review proposed identification and utilization resistant cultivars already adopted by farmers as an immediate strategy for PCN control while waiting for development of new resistant cultivars as a long-term solution to the problem.

## Nematode community structure in the rhizopsheres of Southern California creosote (*Larrea tridentata*)

Pagan, Chris and S. A. Nadler

Dept. Entomology and Nematology, University of California, Davis, CA 95616

### Abstract

Creosote bush (*Larrea tridentata*) is widely distributed across the arid regions of the southwestern United States. These resilient shrubs restructure and enrich the soil beneath them, creating islands of fertility that support relatively dense and diverse nematode communities. We employed a metabarcoding approach to survey nematode diversity and abundance in the rhizospheres of creosote bushes across 11 desert habitats in Southern California. Nematodes were extracted from 64 soil samples using Baermann funnels, and pooled nematodes from each sample were PCR-amplified with 5’ 28S barcoding primers. The amplified products were sequenced on the PacBio Sequel II platform. The resulting sequence data were denoised using the dada2 plugin within the Qiime2 bioinformatic pipeline. Using the sklearn naïve Bayes classifier and a custom 28S reference sequence database, we identified 2319 amplified sequence variants (ASVs) and assigned 92 taxonomic classifications across all samples. There were 61 species-level classifications, although ASVs from some groups, such as Panagrolaimomorpha, are likely overclassified due to underrepresentation in our custom reference sequence database. Patterns in the geographic distribution of ASVs and site-specific drops in the confidence values associated with their classification suggest the presence of additional species or distinct populations. Additionally, we measured 14 soil physicochemical properties to determine whether nematode community composition correlates more strongly with specific abiotic and environmental factors or with geographic location. Preliminary results suggest that geographic location is a weak predictor of community composition, while factors that affect soil texture and water retention are more significant.

## Assessing the impact of organic composted amendments on soil suppressiveness and microbial dynamics in potato early die disease complex

Palmisano, Abigail, L. Parrado and M. Quintanilla

Dept. Entomology, Michigan State University, East Lansing, MI 48823

### Abstract

The synergistic interaction between the root lesion nematode *Pratylenchus penetrans* and the fungal plant pathogen *Verticillium dahliae* causes the devastating Potato Early Die (PED) complex. This detrimental collaboration leads to premature vine senescence, severely diminished yield, and reduced nutrient uptake in potato plants, culminating in yield losses of up to 50%. Our recent laboratory and field trials have indicated that layer ash blend and poultry manure hold promise as effective amendments for controlling PED. It is hypothesized that manure-based amendments from different sources with varying feedstock compositions can promote diverse microbial community profiles and alter nutrient cycling rates in the soil over multiple consecutive growing seasons. This could lead to long-term resistance against pest and pathogen pressures through the development of suppressive soils. To investigate this hypothesis, we conducted a greenhouse experiment in 2023 focusing on the potato early die disease complex. The experiment utilized a randomized block design with six replications. The study evaluated the impact of four organic composted amendments (Morgan’s Compost Poultry manure, Morgan’s layer-ash blend, Vermont Compost Co. blend, Purple Cow compost blend) against the commercially available nematicide Velum, as well as an untreated control population on soil bacterial and fungal communities across two growing consecutive seasons. The incorporation of amendments in soil affects the chemical changes occurring within the soil microbiome, which in turn significantly impacts the fitness and thriving ability of plants, while concurrently reducing nematode populations. Compost and manures led to an increased production of plant hormones that stimulate growth and development. Our laboratory research findings indicate a noteworthy rise in the levels of insecticidal agents, salicylic acid, and metabolic acids in plants that were treated with composted amendments. This highlights the interdependence of the soil microbiome and plant growth, thereby emphasizing the importance of understanding the dynamics of soil microbial communities for successful agriculture. Soil samples were collected at regular intervals to assess the impact of manure-based amendments on soil suppressiveness using various techniques, including targeted-amplicon sequencing, DNA soil extraction, heavy metal analysis, metagenomics, and nematode population counts.

## Sustainable strategies for management of PED (*Pratylenchus penetrans* and *Verticillium dahliae*) in potato production

Parrado, Luisa and M. Quintanilla

Dept. Entomology, Michigan State University, East Lansing, MI 48824

### Abstract

In potato production, the root-lesion nematode, *Pratylenchus penetrans,* can synergistically interact with the wilt-inducing fungus, *Verticillium dahliae*, causing a disease known as Potato Early Die (PED). This disease complex causes plants to senesce 4 to 6 weeks early, thereby causing yield losses between 30% to 50%. The industry standard for PED management is soil fumigation and post-planting pesticides, nonetheless, current management practices are not sustainable. PED remains the top potato industry management priority in Michigan. Different sustainable management strategies to manage PED were examined with a focus on manure-based amendments and biological control agents while emphasizing the importance of preserving soil health. Theffirst experiment examined the effectiveness of integrated management of PED with manure-based amendments and biological control agents in the field. The results show that the most effective management alternatives for *P. penetrans* control are raw poultry manure, both standalone and in combination with a singular application of Vydate^®^ or MeloCon^®^ (a.i. *Purpureocilium lilacinum*), and Compost A in combination with a singular application of MeloCon^®^. Compost A also resulted in higher potato yields, underscoring the necessity of selecting amendments with potential to mitigate pathogen prevalence. The second experiment examined the effectiveness of non-fumigant nematicides, fungicides, and seed treatments against PED under field conditions. Vydate^®^ was the most effective nematicide to control *P. penetrans*, whereas combination of Velum^®^+Velum^®^+Movento^®^+Movento^®^+ Vydate^®^ also slightly decreased *V. dahliae* stem infection. The third experiment examined commercially available biological control agents in Michigan for *Verticillium dahliae* management. *In-vitro* test showed that Tenet^®^, Actinovate^®^, and Elatus^®^ were highly antagonistic to *V. dahliae* isolates tested. However, greenhouse trials suggested that Actinovate^®^ delivers the most effective control. The fourth objective examined the relationship between soil microbiome affected by manure amendment and *P. penetrans* abundance on the potato crop. The results from the greenhouse trials showed that *P. penetrans* abundance in manure-treated soils was significantly lower than the control in both autoclaved or non-autoclaved soils, and the soil microbiome changed in response to both autoclaved and non-autoclaved manure amendments. In particular, the relative abundance of the Firmicutes bacteria phyla significantly increased with the addition of manure-based amendments and negatively correlated with *P. penetrans* abundance. Disease complexes often require an integrated pest management (IPM) approach to ensure maximum control of the primary pests. The results from this research suggested one IPM framework that could be recommended to the potato production industry. This includes soil incorporation of raw poultry manure before planting, early season application of Vydate^®^, and periodic applications of biological control agents like *P. lilacinum* and *S. lydicus* coupled with crop rotation with non-hosts, and meticulous weed, irrigation, and nutrient management. Such IPM practices can foster soil health and ensure sustainable agricultural practices.

## Assessing soil food web dynamics through nematode metabolic footprints in a low-till Sorghum cover cropping system: does variety matter?

Paudel, Roshan and K.-H Wang

Department of Plant and Environmental Protection Sciences, University of Hawaii at Manoa, Honolulu, HI 96822

### Abstract

Sorghum and Sorghum-Sudangrass hybrids (SSgH) possess versatile soil health and pathogen suppression benefits. Harnessing these benefits of SSgH in the tropics is difficult due to the slow soil carbon sequestration rate and rapid reproduction rate of soil-borne pathogens. This is exacerbated by frequent soil tillage preceding every cycle of annual crops. While the general ecosystem benefits of SSgH cover crops are known, the temporal effects of different SSgH varieties on soil food web functions remain largely unexplored. This study aimed to examine seven SSgH varieties for their efficiency in improving soil food web structure over time through the use of nematode community indices and nematode metabolic footprints. Two cycles of SSgH-eggplant (*Solanum melongena*) in successional field trials were conducted at two locations at Poamoho Experiment Station, i.e. a total of 4 field trials were conducted. In each trial, 7 SSgH varieties were grown for 3 months, terminated by strip- and low-till, followed by growing eggplants for 5 months. Nematode metabolic footprints were monitored throughout each cropping cycle from initial sampling, termination of cover crops, mid-term, and termination of eggplant to track changes in soil food web conditions. At the first location, ‘NX2’ and ‘5355’ resulted in the greatest improvement of nematode community structure and metabolic footprint, with ‘NX2’ exhibiting the highest structure footprint at the end of the first cycle of SSgH-eggplant (*P < 0.05*). In the second cycle, all SSgH increased total nematode metabolic footprints, including enrichment and structure footprints, compared to the bare ground control. At the second location, ‘BK’, ‘5355’ and ‘LA’ sorghum planting showed the optimal nematode community footprint after the first cycle, although all sorghum performed better in terms of nematode metabolic footprint compared to bare ground control by the end of the first cycle. In the second cycle, ‘NX2’ and ‘5355’ had the highest enrichment footprint compared to bare ground control (*P < 0.05*), with all sorghum varieties except ‘Piper’ and ‘53514’ exhibiting improved metabolic footprints than bare ground. Overall, ‘NX2’ and ‘5355’ sorghum were most consistent and effective in improving nematode community structure and function across locations. In general, continuous crop rotation of SSgH-eggplant in a conservation tillage system transitioned soil food web conditions from nutrient-depleted and structurally disturbed conditions in Location 1 to nutrient-enriched and more structured conditions after two cropping cycles. Whereas in Location 2, the soil food web started in nutrient-enriched conditions, the planting of SSgH varieties except ‘53514’ transitioned soil conditions towards a more structured food web compared to BG after two cycles. In conclusion, most SSgH varieties outperformed the bare ground at both locations after two cropping cycles, and a combination of different SSgH varieties could achieve maximum soil health benefits in a low-till cropping system.

## Overexpression of *AtPROPEP6* enhances resistance of Arabidopsis against root-knot nematodes

Payal^1^, Sanadhya, K. Minor^1^, J. Kud^1^, A. Huffaker^2^ and F. L. Goggin^1^

^1^Dept. Entomology and Plant Pathology, University of Arkansas, Fayetteville, AR

^2^Dept. Cell and Developmental Biology, University of California, San Diego, CA

### Abstract

Root-knot nematodes (RKNs) are major disruptive pests for worldwide agricultural crop production. The most effective nematode management relies on plant resistance, crop rotation and or chemical nematicides. However, very few sources of resistant germplasm are available for cultivated plants and use of most chemical nematicides is banned due to poor specificity and high toxicity. Thus, there is a pressing need to explore sustainable strategies to enhance nematode resistance in crop plants. Plant elicitor peptides (Peps) derived from precursor protein (PROPEPs) are damage-associated molecular patterns (DAMPs) which activate and amplify the innate immunity of plants against pathogens. There are eight PROPEP genes in *Arabidopsis thaliana* (PROPEP1-PROPEP8) which generate eight Peps (Pep1-Pep8), involved in response to biotic and abiotic stresses. The role of AtPeps is to activate and amplify plant immunity that has been widely established against different microbial pathogens. A previous study using transgenic *Arabidopsis* plants with high constitutive expression of the AtPep1 precursor gene *PROPEP1* indicated an enhanced resistance toward the root pathogen *Pythium irregulare*. In this study, we tested infection by the RKN, *Meloidogyne incognita,* on transgenic lines that over-express *AtPROPEP1*, *AtPROPEP2*, *AtPROPEP3*, and *AtPROPEP6*. All the tested lines showed a reduction in total nematode counts compared to untransformed controls. The strongest reduction was observed in plants overexpressing *AtPROPEP6*, which were further investigated for nematode resistance. Three independent events of *AtPROPEP6* overexpression lines were screened for RKN infection and nematode development. Plants were grown *in vitro* in MS media for 12 days and then infected with RKN J2 juveniles. Thirty days after infection, roots were harvested and stained with acid fuchsin, and the number of galls and juveniles (J3, J4, young females and adult females) were counted for each plant. *AtPROPEP6* expression levels were also compared in the 3 transgenic lines and wild-type controls by RT-qPCR. Nematode infection and development appeared to be inversely related to expression levels of *AtPROPEP6*. Overexpression of *AtPROPEP6* resulted in a statistically significant reduction in the number of galls and total number of nematodes compared to wild-type plants. The percentage of fully developed females was substantially higher in control plants in comparison to transgenics overexpressing *AtPROPEP6*. Further experiments will aim to study gall morphology in transgenic *AtPROPEP6* plants by confocal imaging. This study will help us to explore the potential of Peps to impart resistance against RKN in crop plants.

## Efficacy of nematophagous fungi to control root-knot nematodes

Peetz, Amy^1^, B. Jumbam^2^, V. S. Kunwar^3^, L. Zhang^3^, C. Aime^3^ and I. Zasada^1^

^1^USDA-ARS Horticultural Crops Disease and Pest Management Research Unit, Corvallis, OR 97330

^2^University of Maryland, College of Agriculture and Natural Resources, College Park, MD 20742

^3^Dept. Botany and Plant Pathology, Purdue University, West Lafayette, IN 47907

### Abstract

The search for alternative methods to control plant-parasitic nematodes has been ongoing and many methods are promising. The use of fungi for biological control offers one promising alternative management strategy for crop protection. To identify potential new fungal species with activity against plant-parasitic nematodes, cyst nematode (*Globodera rostochiensis, G. pallida, G. ellingtonae, Heterodera schachtii, H. glycines, H. avenae* and *H. carotae*) populations were collected from North America (Oregon, Idaho, Indiana, North Dakota, Michigan), Africa (Kenya), Europe (France and England), and South America (Peru). Initially, 44 fungal cultures isolated from these cyst nematodes were used to generate filtrates and then tested against *H. glycines* and *G. ellingtonae* eggs. The 26 most promising fungal cultures were then evaluated for ability to suppress *H. glycines* and *G. ellingtonae* J2 viability. Four of the most promising fungi, including species of *Fusarium, Alternaria, Trichoderma*, and *Purpureocillium* were further pursued to determine efficacy against *H. glycines* eggs after 2, 3, and 5 days of exposure. *Alternaria alternata* and *Fusarium acaciae mearnsii* had significant nematicidal effects against *H. glycines*, with 81% and 74% reduction in egg viability, respectively, when compared with the PDA control. Efficacy against second-stage juveniles (J2s) of *Meloidogyne chitwoodi, M. hapla,* and *M. incognita* were also examined after 24 h exposure to these fungi. *F. acaciae mearnsii* had significant nematicidal effects against all *Meloidogyne* J2, with greater than 98.5% reduction in *Meloidogyne* spp. J2 survival compared to the water control. *Alternaria tenuissima* also had significant nematicidal effects, however only on *M. chitwoodi* and *M. hapla*, with 98.9% or greater reduction in survival compared to the water control. *Trichoderma* sp. and *P. lilacinum* did not have any significant effects against any of the *Meloidogyne* spp. tested. Therefore, there is a potential to use *A. tenussima* and *F. acacia mearnsii* for the management of diverse plant-parasitic nematode populations.

## Detection and evaluation of nematode-induced plant stress using remote sensing in red raspberry (*Rubus idaeus*)

Phipps, Savannah^1,2,3^, J. DeLong^4^, I. Zasada^2^, M. H. Hardigan^1^, W. Hoashi-Erhardt^5^, B. Strimbu^6^, D. Bryla^1^ and S. Orr^1^

^1^USDA ARS Horticultural Crops Production and Genetic Improvement Research Unit, Corvallis, OR 97330

^2^USDA ARS, Horticultural Crops Disease and Pest Management Research Unit, Corvallis, OR 97330

^3^Dept. Botany and Plant Pathology, Oregon State University, Corvallis OR 97331

^4^USDA ARS Horticultural Crops Disease and Pest Management Research Unit, Mount Vernon, WA 98273

^5^Washington State University Puyallup Research and Extension Center, Puyallup, WA 98371

^6^Dept. Forest Engineering, Resources, & Management, Oregon State University, Corvallis, OR 97331

### Abstract

Root lesion nematode (RLN), *Pratylenchus penetrans*, is an important pest challenging production of red raspberry (*Rubus idaeus*) in the Pacific Northwest. Chemical treatments have historically been the most effective form of management. However, environmental and public health concerns have reduced the availability of nematicides to growers. Certain alternative chemical control methods such as biofumigation have also proven to be uneconomical or ineffective. While host resistance for RLN can be one of the most economical and sustainable methods of control, the genetic basis for RLN resistance in red raspberry is not well understood. To achieve this, destructive root sampling for nematode quantification and whole-plant pruning at the end of the growing season for aboveground biomass measurements are required to evaluate plant resistance; this is time consuming and detrimental to the plant. High-throughput phenotyping (HTP) using ground-based or aerial imaging may be a promising alternative method for assessing plant resistance to this pathogen. A collaborative effort between the USDA-ARS/Oregon State University, Washington State University, British Columbia Berry Cultivar Development Inc. breeding programs, and the National Clonal Germplasm Repository aims to 1) evaluate the use of mobile and unmanned aerial vehicle imaging for the detection and assessment of RLN-induced stress, and 2) the use of spectral data generated from these technologies for identifying resistance and/or tolerance. Data on fresh weight of aboveground biomass, RLN population densities, and spectral data are being collected from a diversity panel of 270 red raspberry genotypes from the PNW breeding programs and historic germplasm from the National Global Germplasm Repository. To date, two years of biomass data and the first year of RLN population densities and spectral data have been collected. Each raspberry genotype will be sequenced for genome-wide association and genomic prediction in 2025 using three years of field data. Single and multiyear models can then be developed and assessed.

## Nematode communities under stress of extreme alkalinity and changing climate

Porazinska, Dorota L.^1^, K. Gattoni^1^, P. Mullin^2^, K. Powers^2^ and T. O. Powers^2^

^1^Dept. Entomology and Nematology, University of Florida, FL 32611

^2^Dept. Plant Pathology, University of Nebraska-Lincoln, Lincoln NE 68583

### Abstract

The history of life, evolution, and diversity is intricately linked to environmental change and stress. However, in the past century, major global change stressors driven by human activities have been rapidly and irreversibly transforming organisms, communities, and ecosystems. Nematodes are a major component of Earth’s biodiversity. They are the most abundant and diverse multicellular animals. Through their ubiquitous presence, diversity of lifestyles and feeding habits, and positioning at all trophic levels, nematode play important roles in ecosystem functioning. A better understanding of their diversity and function under different environmental conditions and stressors has been increasingly considered critical to predicting their responses, and ecosystems in general, to global change scenarios. The Sandhills Region of Nebraska is one of the largest intact temperate grasslands in the world and biodiversity hotspot. The landscape of the Western Sandhills consists of grass-covered shifting sand dunes interspersed with lakes, both characterized by high alkalinity driven by high-potassium concentrations. The Sandhills are highly sensitive to shifts in climate, especially temperature and precipitation, that can lead to extended periods of drought and result in dune mobilization. We used the Sandhills as a model ecosystem to examine nematode diversity across different habitats under the influence of extreme alkalinity and warming climate. In October of 2019, 2020, and 2021, we collected replicate samples from five lake basins spanning an alkalinity gradient ranging from neutral to highly alkaline (pH 7 – 11). Within each lake basin, samples were taken along a habitat gradient ranging from aquatic (lake and shoreline sediments) to terrestrial (prairie soils). Samples were processed and analyzed for nematode communities, microbial communities, and biogeochemistry. We expected that nematode communities from the three habitats would be distinct, that the drivers shaping these communities would be distinct, and that communities and assembly factors would be moderated by temporal shifts in climate. Our work has indicated that the Sandhills support highly diverse and unique communities, with the consistent presence of many taxa known for their physiological and morphological plasticity. More importantly, the assembly of these nematode communities might be highly context dependent and vary with habitat types, stress levels, as well as nematode functional traits. Moreover, the assembly factors might be strongly moderated by interactions between nematodes and specific microbes and plants, as well as temporal climate driven shifts in precipitation and plant productivity, together complicating our ability to predict these communities in the future at broad scales.

## Unraveling host resistance in dry edible beans against virulent populations of soybean cyst nematode (*Heterodera glycines*) in North Dakota

Poudyal, Prabhat^1^, G. Yan^1^, J. M. Osorno^2^ and H. Kaur^1^

^1^Department of Plant Pathology

^2^Department of Plant Sciences, North Dakota State University, Fargo, ND

### Abstract

North Dakota currently stands as the highest producer of dry beans (*Phaseolus vulgaris* L.) in the U.S. Soybean cyst nematode (SCN; *Heterodera glycines)* has been reported in several counties of North Dakota (ND) and Minnesota (MN) where dry bean and soybean (*Glycine max*) production coincide. Dry bean is a suitable host for SCN and the prevalence of several SCN HG types in North Dakota poses a severe threat to the bean production. The nematode can result in a seed yield reduction of 30 to 56% depending on egg density in soil, environment, and market class of dry beans. Employing host resistance is the ideal strategy to manage SCN, given its dual benefits of cost-effectiveness and environmental sustainability. “ND Falcon”, a pinto bean cultivar developed at NDSU has shown resistance to commonly present SCN populations, HG type 0 (FI = 16.4%) and HG type 7 (FI = 20.2%) in this region. To explore host resistance to the newly reported and highly virulent HG type 2.5.7. in ND, 140 dry bean breeding lines from the NDSU dry bean breeding program were screened twice in growth chamber conditions for 30–40 days depending upon the nematode reproduction in susceptible check. The experiments followed a Randomized Complete Block Design with four replicates. Seeds germinated for 3–4 days were planted in plastic cone-tainers and each plant was inoculated with 2,000 SCN eggs collected from a field in Traill County, ND. Soybean Barnes was used as a susceptible check. The female index (FI = average number of females on test line/average number of females on susceptible check × 100%) was calculated and disease resistance ratings were assessed. Dry bean genotypes belonging to the Andean gene pool, which consists of kidney beans had FI ranging from 63.0% to 92.5% across two trials. The genotypes from the Middle-American gene pool which includes popular market classes such as black, pinto, and navy beans had FI ranging from 39.2% to 84.0%. Among the four market classes screened, black beans exhibited the lowest susceptibility to SCN, followed by pinto, navy, and kidney beans. However, no sources of resistance were identified among these genotypes to HG type 2.5.7. To broaden the search for resistance, a comprehensive literature review was conducted, resulting in the selection of 60 additional accessions from the USDA common bean core collection. This collection contains beans from Latin America, where dry beans first originated. Trials involving these accessions are currently underway. The outcomes of these trials will offer new insights into SCN reproduction in dry beans, laying the foundational knowledge for identifying and integrating resistance traits into new cultivars. This research holds significant information for selecting dry bean breeding lines and cultivars with lower susceptibility to combat this damaging disease in SCN-infested fields.

## Tracking persistence in native and commercial strains of entomopathogenic nematodes

powers, Thomas^1^, J. Peterson^2^, A. Lyons^2^, T. Harris^1^, C. Matlock-Carter^1^ and K. Powers^1^

^1^University of Nebraska, Dept. of Plant Pathology, Lincoln, NE 68583

^2^ West Central Research and Extension Center, North Platte, NE 69101

### Abstract

The western corn rootworm (WCR), *Diabrotica virgifera virgifera*, is a major insect pest of corn production in the United States. Annual costs in yield loss and management in the US are estimated to exceed 1 billion dollars. In Nebraska, WCR has developed resistance to a wide range of insecticides, including pyrethroids and multiple Bt proteins. EPNs are now receiving renewed attention as a WCR biological control. As part of a four-state USDA NIFA project, we are addressing questions of EPN persistence, using commercial strains of *Heterorhabditis* and *Steinernema*. DNA barcoding of the mitochondrial COI gene is being used to identify and monitor EPN strains. Early observations from field trials and surveys indicate that there are three native strains of EPNs existing across a six-county region in central and southwestern Nebraska.

The native strains consist of two *H. bacteriophora* strains and one undescribed species of *Steinernema*. These strains were initially observed based on their presence in unapplied nematode control plots, conservation grasslands (CRP fields), and surveys of fields with no history of EPN application. Both native strains of *H. bacteriophora* are distinct from GenBank accessions of the species and did not match strains screened from a USDA collection of *H. bacteriophora* or commercial products from Persistent BioControl or Arbico Organic’s product NemaSeek. The undescribed *Steinernema* species is distinct from any GenBank accession and does not match USDA reference strains of *S. carpocapsae* or *S. feltiae*, nor does it match Persistent Biocontrol’s *Steinernema* products or Arbico Organic’s product NemAttack. Evidence of persistence of the native strains of *H. bacteriophora* and *Steinernema nsp*. stems from their recovery in production fields since 2015. Evidence of persistence of the commercial *Steinernema feltiae* product from Persistent BioControl results from their recovery in two inoculated fields 500 days after application. In the 2024 field season, these studies are being extended across a four-state region.

## Investigating nematode biodiversity in the irish boglands

Pulavarty, Anusha^1^, T. Klappauf^1^, D. McMillan^2^ and T. Kakouli-Duarte^1^

^1^Molecular Ecology and Nematode Research Group, enviroCORE, Department of Applied Science, South East Technological University, Carlow, Ireland

^2^Green Restoration Ireland Cooperative Society Ltd, Carlow, Ireland

### Abstract

Natural bogs and peatlands play a major role in reducing global warming, as they are natural carbon sinks. Approximately 1.2 million of Ireland’s 1.5 million hectares of peatlands are damaged to different degrees, disturbing the natural flora and fauna of these ecosystems and releasing sequestered carbon dioxide back into the atmosphere contributing to climate change. Numerous rewetting and restoration programs have been implemented throughout Ireland and Europe with the goal of rehabilitating degraded bogs. The project’s enterprise partner, Green Restoration Ireland (GRI) cooperative, is directly working to reestablish Irish natural heritage, help fight climate change and restore biodiversity and ecosystem services. The SETU’s Molecular Ecology and Nematode Research Group are assisting GRI in evaluating their peatland restoration program by studying the nematode diversity in intact, restored and non-restored peatlands. Overall, 14 different peatland habitats including raised bogs and fens have been sampled to extract nematodes from the peat soils. A specific extraction procedure has been optimized and standardized to extract nematodes from the peat soils. The extracted nematodes have been permanently fixed on to glass slides using formaldehyde, ethanol and glycerin. The fixed nematodes were studied using a high-power light microscope to morphologically identify the families, genera and species present in Irish peatlands. Nematode analysis was also done using molecular tools. The peatland habitats, irrespective of their ecological status, were dominated by bacterivores, however, the next most prevalent trophic groups in the wasted peat are fungivores and herbivores. The healthy peatland sites were dominated by bacterivores, followed by predatory species but had a very low percentage of herbivores. As per the morphological analyses so far, there are no significant changes noted in the ecological status of rewetted areas compared to non-rewetted sites. This implies that the rewetted habitats will require additional time for restoration. However, according to NINJA analysis, both molecular and morphological data reveal that both healthy and degraded raised bog habitats have a suppressive, fertile and a matured soil environment, whereas wasted peat is a degraded, depleted and conducive habitat. Alongside the main research findings, eight plant-parasitic nematode families were found in peatland sites, including those of economically significant pests, such as Meloidogynidae, Pratylenchidae, and Heteroderidae. This finding might be useful for the policymakers and farmers to consider the potential impacts of the plant-parasitic nematodes while diversifying Ireland’s agriculture. These preliminary findings provide an overall idea of nematode biodiversity in various bog habitats. The study is in progress and these initial observations will further help to evaluate the ecological status of the restored and non-restored Irish peatland sites.

## Intragenomic sequence variations in the small subunit (SSU) ribosomal DNA of *Tylenchus arcuatus* (nematoda: tylenchida)

Ramsay, Kimberly^1^, C. Grenier^1^, B. Pearce^1^, R. Alfeche^1^, I. Gymninova^1^, Q. Yu^2^ and F. Sun^1^

^1^Canadian Food Inspection Agency, Ottawa, ON, Canada

^2^Ottawa Research and Development Center, Agriculture and Agri-Food Canada, Ottawa, ON, Canada

### Abstract

The genomic region for the small subunit ribosomal RNA (SSU or 18S) is widely used in the phylogenetic reconstruction and the identification of closely related nematode species. Understanding the levels of intragenomic variation in the region among the rDNA repeats, which are tandemly arranged in the genome, is crucial for accurate species delimitation. In an attempt to characterize a population of *Tylenchus arcuatus*, a 902 bp PCR product corresponding to the 5-prime portion of SSU region was amplified from the DNA extracted from a single nematode. However, direct sequencing of the PCR product resulted in an ambiguous DNA sequence chromatogram, suggesting the presence of intragenomic variations in the region. To assess the magnitude of these variations, the PCR product was cloned and sequenced. Among the 52 clones analyzed, 48 unique haplotypes were observed. The pairwise divergence of these haplotypes ranged from 0.1% to 15.7%. Approximately 64% of the haplotypes exhibited a variation greater than 5%. These variations were caused by substitutions (136 nucleotides) and/or indels (88 nucleotides). Over 50% of the total change events occurred in the V2 and V4 domains, with 32.5% in V2 domain and 36.4% in V4 domain. The implication of the significant intragenomic variation in the use of the SSU region as a molecular marker for *Tylenchus* species delimitation or phylogenetic analyses is discussed.

## Genetic specificity between *Meloidogyne arenaria* and *Pasteuria penetrans* is altered by temperature

Ramirez, Abbey and A. K. Gibson

Dept. Biology, University of Virginia, Charlottesville, VA

### Abstract

Plant-parasitic nematodes in the genus *Meloidogyne* cause billions of dollars per year in damage to crops worldwide. We are in substantial need of new control methods as resistant crop cultivars are failing, and nematicides are increasingly restricted due to environmental damage. *Meloidogyne* has a natural parasite that offers a promising solution as a biological control for these destructive nematodes. *Pasteuria penetrans* is an endospore-forming bacteria that resides in the soil and attaches to the cuticle of *Meloidogyne. P. penetrans* then invade the host body, ultimately destroying the nematode’s reproductive system. As promising as this sounds for potential biological control, field trials have had mixed results. These variable results may stem from the genetic specificity of the interaction: each *Meloidogyne* genotype is only compatible with a subset of bacteria genotypes. Furthermore, this genetic specificity may be altered by environmental conditions, with repercussions for the effectiveness of control mechanisms over a growing season. While the genotypic specificity of this system is clear, we do not know how stable it is to environmental conditions. Using *Meloiodogyne arenaria* and *Pasteuria penetrans,* we specifically tested whether the observed genetic specificity is robust to variation in temperature. We tested the genetic specificity of six isofemale lines of *M. arenaria* and five sources of *P. penetrans*. Each combination was tested at three temperatures. Infection probability was then estimated as attachment rate of *P. penetrans*. We found that temperature does significantly affect genotype specificity within this system. Hosts become more susceptible at high temperature, collapsing variation in infection. These results suggest that higher genetic variation of *P. penetrans* will need to be applied to crop fields at cooler temperatures, and that it will need to be applied at multiple time points in the nematodes’ reproductive season.

## Developing a real-time PCR assay for direct detection and quantification of the new root-lesion ematode, *Pratylenchus dakotaensis* from soybean plant roots

Ranabhat, Siddant, A. Plaissance and G. Yan

Dept. Plant Pathology, North Dakota State University, Fargo, ND 58108

### Abstract

Root-lesion nematodes (*Pratylenchus* spp.) pose a significant threat to soybean crops because they can cause yield losses by infecting and damaging plant roots. *Pratylenchus dakotaensis*, a newly named root-lesion nematode species found in a soybean field of North Dakota exhibited high infection and reproduction in certain soybean cultivars through greenhouse bioassays. Early and rapid detection of *P. dakotaenesis* is essential for managing this nematode. However, traditional methods for identification and quantification are laborious and time consuming. Detection assays that can identify *P. dakotaenesis* directly from infected soybean roots are important as more than 50% of the *P. dakotaensis* population was found to reside in roots. Thus, this research aims to develop a sensitive and specific SYBR Green based real-time PCR assay for the rapid detection and quantification of *P. dakotaenesis* from soybean roots. The assay utilized specific primers (IC-ITS1F/IC-ITS1R) that target the internal transcribed spacer (ITS) region of the ribosomal DNA, previously designed in our lab for detecting this species in DNA extracted from nematode individuals. This assay differs from previous assays as it utilizes DNA extracted directly from soybean roots using FastDNA Spin Kit. The specificity of the assay was assessed using plant root DNA extracts, three other *Pratylenchus* spp. and five common plant-parasitic nematodes present in soybean fields of North Dakota and Minnesota. The specificity test showed that only *P. dakotaensis*-inoculated root DNA extracts were amplified and none of root DNA extracts with other control species were amplified. The sensitivity test was conducted, and its analysis revealed the assay could detect up to 1/128^th^ of a single nematode inoculated into 0.2 g of uninfected soybean roots. A standard curve was generated relating quantification cycles (Cq) with log number of *P. dakotaensis* from artificially inoculated soybean roots, showing a strong correlation (R^2^=0.97) between the Cq values from real-time PCR and nematode numbers, with a high amplification efficiency (E=98.1%). Furthermore, the assay was able to detect *P. dakotaensis* in the DNA extracted from the roots of eleven soybean cultivars grown in infested field soil and harvested from a greenhouse screening trial. The developed assay is being validated using different numbers of nematodes inoculated into uninfected plant roots and more infected soybean roots from a repeated greenhouse trial. Overall, the assay was found to be specific and sensitive with high efficiency. This new assay will not require expertise in morphological identification of this species and has the great potential to help in rapid detection and quantification of *P. dakotaensis* directly from infected soybean roots, thereby facilitating timely management of this new nematode pest.

## Votivo, *Bacillus firmus* I-1582, fifteen years of commercial success

Riggs, Jennifer

BASF, Global R&D Management Seed Treatment, RTP, NC 27709

### Abstract

*Bacillus firmus* strain I-1582 (BFI), Votivo®, is a natural plant growth promoting rhizobacteria isolated from cultivated soils in Israel. Votivo is registered as a pesticide that can provide protection against a range of plant pathogenic nematodes through multiple modes of actions. Votivo was launched in 2010 as a seed treatment and soil applied product in the USA, followed by registrations in Canada, Europe, Mexico and Brazil as a biocontrol microbe. *B. firmus* I1582 colonizes the plant root and the number of bacteria colonies per root system increases during the root development. The bacteria minimize the attractiveness of the roots for nematodes by consumption of root exudates, which are used by the nematodes for orientation. The development of BFI following soil introduction results in production of phytohormones that can influence plant growth above and below the soil, as well as production of hydrolytic enzymes that can have direct impact on nematode eggs. After 15 years, millions of agriculture acres are still being treated with this important biocontrol microbe that is compatible with traditional fungicides and insecticides on multiple seeds. The long-term commercial success of the product can be contributed to the biology as well as the formulation science that allows for long term stability of BFI as a liquid product in a container as well as a stable product that once applied to the seed can remain viable for multiple years.

## Investigating management strategies for the seed gall nematode, *Anguina funesta*, parasitizing annual ryegrass seed (*Lolium multiflorum*) in oregon

Rivedal, Hannah^1^, T. Temple^1^, R. Starchvick^2^, E. Braithwaite^2^, T. Benedetti^2^, J. Gallagher^1^, W. Davis-Hinze^2^, A. Peetz^3^ and I. Zasada^3^

^1^USDA-ARS Forage Seed and Cereal Research Unit, Corvallis, OR 97331

^2^Oregon State University, Dept. of Botany and Plant Pathology, Corvallis, OR 97331

^3^USDA-ARS Horticultural Crops Disease and Pest Management Research Unit, Corvallis, OR 97330

### Abstract

Oregon’s grass seed industry specializes in producing forage grasses including annual ryegrass (ARG, *Lolium multiflorum*), a host for the seed gall nematode (SGN, *Anguina funesta*). SGN caused yield-limiting seed galls and are strictly regulated in trade. From 2019 to 2020, over 1 million pounds of seed were rejected from international ports due to SGN detection. Galls contain hundreds of nematodes, a source of infection for next year’s crop. Harvested galls and those that shatter to the soil allow for the spread and continuation of the disease cycle. A 2022 field survey of ARG grown in the Willamette Valley of Oregon resulted in *A. funesta* detection in 11 of 22 fields. Investigations into the control of SGN are challenging due to an inability to successfully culture SGN in the lab, and a lack of control resources. Several approaches to culturing *A. funesta* are under evaluation. First, to efficiently generate galls, plants are being grown in tissue culture, or the greenhouse, under growth regulation to keep the plants small, inoculating with infective stage juveniles (J2), followed by the addition of gibberellic acid (GA3) to induce flowering. Second, to mimic field conditions, outdoor soil beds have been seeded with ARG and successively inoculated with J2s. Successful culturing of SGN will provide a source of inoculum for future experiments. To date, no nematicides are labeled for use for control of SGN. Several nematicides with unknown efficacy against SGN were tested in repeated plate bioassays against J2s liberated from a seed gall. Three chemistries were nematicidal after 48 hours, fluopyram, fluensulfone, and cyclobutrifluram, killing all the exposed juveniles at 50, 100, and 150% of the labeled rate. Two chemistries were nematistatic after 48 hours, fluazaindolizine and abamectin, killing between 15 and 63% of exposed juveniles at 100% of the labeled rate.

Phytotoxicity tests resulted in no damage to ARG for both fluopyram and cyclobutrifluram.

These two nematicides, as well as a no nematicide control, are being evaluated in two production fields with histories of SGN, in a trial established April 2024. Fields were sampled for SGN before nematicide application and two weeks post application to determine the effects on in-plant detections of SGN. Yield and galled seed data will be collected at harvest in July 2024. Alternative control methods are also being considered, including utilizing high energy pulses on seed galls. Preliminary data suggests that this could be a viable treatment to include in a seed cleaning production setting. Successful control options for the SGN in ARG seed production is important to reduce the spread of this nematode globally and maintain healthy forage production.

## Using a community-level sampling approach for detecting *Meloidogyne enterolobii* and other pathogens in sweetpotato storage roots

Rutter, William^1^, J. Culbreath^1^, C. Khanal^2^, P. A. Wadl^1^, S. L. Kai^1^ and J. Mueller^3^

^1^USDA-ARS United States Vegetable Laboratory, Charleston, SC, 29414, USA

^2^Dept. Plant and Environmental Sciences, Clemson University, Clemson, SC, 29634

^3^Edisto Research and Education Center, Dept. Plant and Environmental Sciences, Clemson University, Blackville, SC, 29817

### Abstract

The recent outbreak of *Meloidogyne enterolobii* in the sweetpotato industry provides a startling example of how nursery stocks can vector the spread of a plant-parasitic nematode. *M. enterolobii* has been found in sweetpotato production fields in 11 counties in North Carolina and in sweetpotato producers’ fields in four other states. Preventing the spread of this nematode to new sweetpotato fields is a major challenge because growers often use storage roots as “seeds” to propagate their crop each year, and a single nematode-infected seed root can permanently infest a field. In the past, discriminating *M. enterolobii* from other *Meloidogyne* species that infect sweetpotato has relied primarily on PCR conducted by trained personnel using individual nematodes. However, these methods are limited in their ability to survey for the presence of a specific species in large batches of infected roots that can easily contain thousands of individual nematodes in mixed species populations. We developed a community-level sampling method that allows us to detect even low levels of *M. enterolobii* infection in large batches of infected sweetpotato roots, without extracting and testing individual nematodes. We tested this method using a variety of controls including positive controls in the form of artificially *M. enterolobii* infected sweetpotato roots, negative controls in the form of uninfected sweetpotato roots, and spike in controls in the form of *M. enterolobii* eggs added directly to processed uninfected sweetpotato skin samples. Using qRT-PCR we were able to detect the presence of *M. enterolobii* in all the positively infected control batches, even when no visible signs of root-knot nematode infection were present. Moreover, we were also able to detect as few as two *M. enterolobii* eggs spiked into a 10-mL sweetpotato skin sample. We also used this method to successfully survey for *M. enterolobii* and other *Meloidogyne* species in fresh market sweetpotatoes. Our results show that this new survey method could be a useful tool to help slow the spread of *M. enterolobii* to new sweetpotato growing regions. We are currently working to adapt this method to help detect additional viral and nematode pathogens that infect sweetpotato, and perhaps use similar community-level approaches to survey for *M. enterolobii* in other nursery plants.

## Conspecific and heterospecific interactions between two plant-parasitic nematodes on sugar beets

Sadic-Ulu, Busra^1,3^, T. C. Ulu^1,2^, L. Ripa^1^, G. Stevens^1^ and E. Lewis^1^

^1^College of Agricultural and Life Sciences, Dept. Entomology, Plant Pathology and Nematology, Moscow, ID

^2^Faculty of Agriculture and Natural Sciences, Department of Plant Protection, Bilecik Seyh Edebali University, Bilecik, Turkiye

^3^Transitional Zone Agricultural Research Institute, Department of Plant Health, Eskisehir, Turkey

### Abstract

In nature, plant-parasitic nematodes are attracted or repelled from the host by certain chemical signals. Although many signals affect plant-parasitic nematode (PPN) behaviors, signals emitting from the host and pathogens near the host have huge impact on interactions of PPN. This study aimed to investigate how exudates and volatiles (VOCs) from PPN-exposed and non-exposed sugar beet roots affect orientation behaviors of two important PPN species, root knot nematode (RKN) *Meloidogyne incognita* and root lesion nematode (RLN) *Pratylenchus neglectus*. Moreover, heterospecific and conspecific impact of exometabolomes on PPN behavior was assessed. Nematode preferences were determined to root exudates and exometabolomes by setting up two-choice petri dish experiments. To test the impact of VOCs, olfactometer experiments were conducted by placing 7-day control and nematode-exposed plants at the end of olfactometer arms to assess the nematodes’ choices over an 18-hours for RKN and 48-hours for RLN. For root exudate experiments, no significant trend was observed in the preferences of RLN. Conversely, RKN exhibited a significant preference for the clean plant root exudates over roots expose 1 day’s post RKN inoculation (DPI), clean versus RLN exposed, 2 DPI and empty versus clean. In the exometabolome tests, no significant results were observed for either nematode species. In olfactometer trials, RKN consistently preferred non exposed option over PPN-exposed plants, whereas RLN displayed no preferences. This study underscores that chemical signals from root exudates and VOCs, have a crucial impact on the conspecific and heterospecific behavior of plant-parasitic nematodes.

## Past, present and future of nematode indicators of soil health under stress conditions

Sánchez-Moreno, Sara

Laboratory of Nematology. Dept. Biodiversity and Evolutionary Biology, National Museum of Natural Sciences (MNCN-CSIC), Madrid, Spain

### Abstract

In the last 30 years, nematode-based indices (NBIs) have been extensively used to assess the effects of varying soil perturbation on soil nematodes, and the number of published articles per year has significantly increased over time. While early works focused on the effects of pollution and conventional agricultural management on nematode community structure at a local scale, the focus has gradually changed towards large-scale studies and the inclusion of natural ecosystems. Nematologists have been able to establish a solid body of knowledge that explains the response of nematode functional diversity to a wide range of environmental filters and the relation between nematode community structure and ecosystem functions, especially (but not only) under anthropogenic impacts. Nematode ecology has evolved and new scientific challenges have appeared: 1) The efficient integration of molecular and morphological identification processes, and the application of NBIs to molecular data; 2) Their use as indicators of ecosystem services in new ecological scenarios; 3) The applicability of global nematode data; 4) The exploration of undiscovered nematode diversity (especially in the global South) and 5) The effects of the ongoing global extinction process on soil nematodes. Nematodes are widely recognized as a key biological component of soils, and a crucial element in the scientific assessment of soil quality, with emergent implications on public policies. In recent years, new social and political questions in relation to soil biodiversity conservation have arisen. As nematologist, we should use the accumulated knowledge on nematode bioindication to help solving such questions, including the mitigation and adaptation strategists to protect soil and nematode diversity under climate change.

## Soil health as affected by winter cover crops on sweetpotato yield in Southern U.S.

Schloemer, Claire^1^, K. S. Lawrence^1^, S. H. Graham^1^, B. S. Sipes,^2^ and K. H. Wang^2^

^1^Auburn University, Auburn, AL

^2^University of Hawai’i at Manoa, Honolulu, HI

### Abstract

Common production practices for sweetpotato are destructive to soil health due to the needs of raised bed planting and digging at harvest. Replenishing soil organic matter through winter cover cropping offers an option to manage soil health in the Southern U.S. where most sweetpotato production occurs. Strategies to select cover crops for soil health management are mixed between high biomass production, high biodiversity, crop yield improvement vs plant-parasitic nematode suppression. Two field trials were conducted in 2022 and 2023 at Brewton, AL and Dobson, NC to determine the effect of winter cover crops on soil health. The winter cover crop treatments included: crimson clover (*Trifolium incarnatum*), daikon radish (*Raphanus sativus*), wheat (*Triticum aestivum*), ‘Elbon’ rye (*Secale cereale*), 4-way mix of crimson clover, daikon radish, ‘Elbon’ rye, and wheat, compared to a fallow control. ‘Beauregard’ sweetpotato was then planted into all plots for 3 months prior to harvest. Nematode community analysis and microbial phospholipid fatty acid (PLFA) assay were performed as bioindicators of soil health throughout each cropping cycle in both locations but only data from NC are presented in this abstract. Crimson clover increased abundance of bacterivorous nematodes throughout the crop (*P* ≤ 0.05), omnivorous and predatory nematodes numerically (*P* > 0.05) and structure index (SI, *P* ≤ 0.05) at the end of the crop compared to fallow. These results indicated crimson clover stimulated more bacterial decomposition and a more structured soil food web. The mix cover cropping (MIX) increased abundance of fungivorous nematodes initially, but crimson clover later had the highest abundance of fungivorous nematodes at the peak season of sweetpotato growth (*P* ≤ 0.05), indicative of successional of decomposition from bacterial to fungal by crimson clover. Microbial PLFA assay revealed that MIX initially increased G-bacteria and saprophytic fungi biomass (*P* ≤ 0.05), indicative of stress and resilient residues more difficult for decomposition. However, MIX eventually reduced stress and increased arbuscular mycorrhizal fungi biomass throughout 2023 (*P* ≤ 0.05). MIX also enhanced (*P* ≤ 0.05) soil microbial respiration based on Solvita Burst test. On the other hand, wheat had the highest saprophytic fungi and G+ bacteria biomass (*P* ≤ 0.05), indicative of stressful soil conditions. Canonical correspondence analysis among soil health indicators and sweetpotato yield in 2023 (first two canonical axes explained 82.38% of the variation) showed that sweetpotato yield was positively related to SI, abundance of omnivorous nematodes, nematode diversity, S/U (saturated/unsaturated microbial PLFA indicative of less stressful conditions). These data suggested that sweetpotato yield better when soil food web was less stressed or disturbed but was reduced by abundance of root-knot nematodes. While crimson clover improved soil health within two cropping cycles, its susceptibility to root-knot nematodes hinders its benefit to sweetpotato production. Therefore, mix planting of crimson clover with three other winter cover crops was more profitable for sweetpotato.

## Winter cover crops and biological products to manage *Meloidogyne incognita* and promote soil health in sweetpotato

Schloemer, Claire^1^, K. S. Lawrence^1^, S. H. Graham^1^, B. Lawaju^1^, B. S. Sipes^2^ and K. -H. Wang^2^

^1^Auburn University, Auburn, AL

^2^University of Hawai’i at Manoa, Honolulu, HI

### Abstract

Organic production is increasing across the Southeast, but there is a need to develop effective organic integrated nematode management for sweetpotatoes. To address this, field trials were established in Brewton, Alabama in a Benndale fine sandy loam soil and Dobson, North Carolina in a Fairview cobbly sandy clay loam to determine the effect of selected winter cover crops and biological products in the suppression of nematode and insect pests. This was accomplished by planting sweetpotatoes into the footprints of selected winter cover crops. Entomopathogenic nematodes (*Steinernema feltiae*, *S. carpocapsae*, and *Heterorhabditis bacteriophora*), and fungi *Beauveria bassiana* and OMRI Majestene bionematicide were applied to half of each plot to determine their combined ability to suppress pests. Ultimately, the cover crop mix of crimson clover, daikon radish, elbon rye, and wheat was associated with high marketable yields (+ 2000 lb/A increase over fallow), low insect damage, and lower *Meloidogyne incognita* populations. The three-way mix of biological products numerically increased marketable yields in both locations and significantly reduced internal *M. incognita* damage in Alabama. In Alabama, the legume winter cover crops crimson clover and field peas supported higher *M. incognita* populations at sweetpotato planting with 97 and 368 J2/100 cm^3^ soil, respectively. The remaining cover crops supported only 1–78 J2/100 cm^3^ soil. Total *M. incognita* populations throughout the seasons increased on crimson clover (454 J2/100 cm^3^ soil) and field peas (863 J2/100 cm^3^ soil) but was lowest on daikon radish (345 J2/100 cm^3^ soil). In North Carolina, *M. incognita* populations were lower at sweetpotato planting ranging from 28 to 72 J2/100 cm^3^ soil for fallow and crimson clover. Total *M. incognita* populations across the season were significantly lower on elbon rye, wheat, and the cover crop mix compared with crimson clover. All cover crops in North Carolina increased free-living nematodes compared to the fallow. Across cover crops in both locations, bacterivores were most numerous followed by fungivores and predators. Soil microbial respiration estimated by CO_2_ Burst (Solvita test) increased over the season, and in Alabama the highest soil respiration was recorded near harvest. In North Carolina, the highest soil microbial respiration was observed in the cover crop mix and lowest in the fallow. Total living microbial biomass estimated by phospholipid fatty acid (PLFA) assay ranged from 14,076 to 16,105 ng/g in North Carolina but was lower in Alabama ranging from 2,925 to 3,495 ng/g. Soil textural differences between locations probably contributed to these differences. Higher soil microbial activities in the North Carolina cover crop mix resulted in higher sweetpotato yield, lower *M. incognita* populations, higher soil microbial respiration, and higher total microbial PLFA biomass, though these cover crop effects were not obvious in the Alabama Trial. None-the-less, the biological control products significantly reduced internal *M. incognita* damage in Alabama and numerically increased yields across both locations.

## *Solanum sisymbriifolium* soil amendment effect on *Globodera pallida*

Schulz, Lindsay L.^1^, H. V. Baker^2^, I. A. Zasada^2^ and L. M. Dandurand^1^

^1^Department of Entomology, Plant Pathology and Nematology, University of Idaho, Moscow, ID 83844

^2^USDA-ARS, Corvallis, OR 97330

### Abstract

*Globodera pallida* is a quarantined pest of potato in Idaho, USA. This economically devastating pest of potato was first found in Idaho in 2006. The goal of the eradication program is to contain and eradicate infestation in Idaho. Many different nematicides including methyl bromide have been banned due to environmental and health hazard concerns. Therefore, new methods for control that are effective for eradication and more environmentally friendly are needed. *Solanum sisymbriifolium* also known as litchi tomato, is a trap crop for *G. pallida*, inducing hatch but reducing reproduction by 99% in potato when planted after litchi tomato. However, producers are hesitant to use it as a trap crop because *S. sisymbriifolium* seeds are largely unavailable for field use and *S. sisymbriifolium* does not produce an economically viable crop. Previous research indicates that glycoalkaloids found in *S. sisymbriifolium* may be the cause of this toxic effect. Additionally, extracts from stem/leaf material of 8-week-old *S. sisymbriifolium* was toxic to *G. pallida, in vitro*. This research evaluated *S. sisymbriifolium* dried stem/leaf and root material applied as a soil amendment at 0%, 2.5%, and 5% in a greenhouse trial planted with the potato cultivar ‘Russet Burbank’. Infection was calculated through root staining 6 weeks post planting via acid fuchsin staining. This research shows that amending soil with *S. sisymbriifolium* dried stem/leaf plant material at 5% significantly reduced root infection by 87% compared to non-treated controls. Additionally, stem/leaf material at 2.5% amendment reduced infection by 79%. Root material did not significantly reduce root infection. The discovery of novel methods for the eradication of *G. pallida* is essential for Idaho producers and soil amendments using *S. sisymbriifolium* may be a promising strategy.

## *Solanum sisymbriifolium* extract effects on *Globodera pallida*

Schulz, Lindsay and L. M. Dandurand

Dept. Entomology, Plant Pathology and Nematology, University of Idaho, Moscow, ID 83844

### Abstract

*Globodera pallida* is an economically devastating pest of potato first found in Idaho in 2006. As a regulated pest, the goal is to contain and eradicate the infestation. Environmentally hazardous nematicides have been used as control measures, but new environmentally friendly methods of control are needed. By inducing hatch and inhibiting reproduction, *Solanum sisymbriifolium* is a trap crop for *G. pallida*. However, because *S. sisymbriifolium* is largely unavailable for field use and it is not an economically viable crop, growers are hesitant to use it as a trap crop. Previous research indicates that glycoalkaloids found in *S. sisymbriifolium* may have a toxic effect on *G. pallida*. This research tested chemical extracts of *S. sisymbriifolium* created with solvents of increasing polarity for their toxicity to *G. pallida* via hatch assays. Butanol fractions of the 8-week-old stem and leaf of *S. sisymbriifolium* were most toxic causing 72% hatch reduction. Four- and six-week-old plants that were extracted via the same method did not cause a significant difference in hatch. However, extracts were also made from *S. sisymbriifolium* infected with *G. pallida*. When infected, *S. sisymbriifolium* stem and leaf butanol extracts from 4-week-old plants reduced hatch by 40% and butanol extracts from roots reduced hatch by 52% compared to the potato root diffusate control. Extracts from infected 6-week-old *S. sisymbriifolium* plants did not cause a significant difference in hatch. This research indicates that plant secondary metabolites are being produced and appear to be toxic to *G. pallida*. Additionally, these secondary metabolites appear to be produced later in uninfected plants. This research has the potential to contribute to the discovery of novel environmentally friendly nematicides for use in *G. pallida* infested fields in Idaho and around the world.

## The influence of irrigation, crop rotation, and fluopyram nematicide on peanut yield and the nematode community

Schumacher, Lesley^1^, I. Small^2^ and Z. Grabau^3^

^1^USDA-ARS, Tifton, GA 31793

^2^University of Florida, Dept. of Plant Pathology, Quincy, FL 32351

^3^University of Florida, Dept. of Entomology and Nematology, Gainesville, FL 32611

### Abstract

Peanut (*Arachis hypogaea*) is an important cash crop in the southeastern United States and suffers from yield losses due to plant-parasitic nematodes. Peanuts are rotated with two years of cotton (*Gossypium hirsutum*) or one year of cotton and two years of sod (*Paspalum notatum*) in conventional and sod-based crop rotation, respectively. Little is known about how three common agronomic practices – irrigation, crop rotation, and fluopyram nematicide application – collectively influence peanut yield and nematode community structure. Therefore, objectives of this research were to determine effects of irrigation (with or without), crop rotation (conventional or sod-based peanut), and fluopyram nematicide application (with or without) on various nematode feeding groups, ecological indices, and peanut yield. Soil samples were collected before planting, at midseason, and at harvest in 2018–2019 at a long-term research site in Quincy, FL, USA. Free-living and ring nematodes (*Mesocriconema ornatum*) were extracted from a subsample using sucrose-centrifugation and nematode ecological indices (structure, maturity, channel, enrichment, and basal) were calculated. Overall, ring nematode population densities were greater in sod-based peanut than conventional peanut. Conventional peanut had greater yield than sod-based peanut plots. Fluopyram nematicide application did not improve peanut yield compared to untreated plots. We observed consistent trends with sod-based peanut increasing fungivores relative to conventional peanut. Yet, other nematode feeding groups and ecological indices were not consistently impacted by our factors. Therefore, nematode ecology based on feeding groups was not heavily influenced by irrigation, crop rotation, or fluopyram nematicide in this research.

## Loop-mediated isothermal amplification (lamp) based rapid identification and sensitive detection of potato cyst nematodes

Shah, Chandni and L. M. Dandurand

Dept. Entomology, Plant Pathology and Nematology, University of Idaho, Moscow, ID 83844

### Abstract

The regulated pests, *Globodera pallida* and *Globodera rostochiensis*, have acquired significant importance globally due to their devastating effect on potatoes. *Globodera ellingtonae*, recently reported in Idaho and Oregon (USA), and although, potato is a good host, it appears to be non-pathogenic on US potato varieties. Early detection of *Globodera* spp. at the species level is crucial to prevent further spread of the disease, and to execute and adopt the necessary management strategies. Cysts of all *Globodera* species after maturation look similar and are often difficult to distinguish morphologically. In the present study, we have developed a highly specific, sensitive and rapid loop-mediated isothermal amplification (LAMP) assay which accurately discriminates *G. pallida* from other *Globodera* species in less than 30 min. LAMP assay is a newly developed species-specific gene amplification method, which uses four primers to amplify the Chorismate mutase genes (*CM*) and internal transcribed spacer region (ITS) of rDNA. For development of the LAMP assay, the reagent concentration of MgSO4, dNTPs, and Bst DNA Polymerase and reaction condition such as amplification time and temperature were optimized. The optimized LAMP assay efficiently detects *G. pallid*a at 10 fg μL^−1^, 1000 times lower in concentration compared to conventional PCR 10 ng μL^−1^ and the amplified product can be analyzed through visible fluorescent green color using SYBR Green I dye on 1.5% agarose gel electrophoresis within 30 min. This assay is cost-effective, target specific, reliable, and provides insights in the early detection of *Globodera* spp.

## Surveillance, characterization and ecosafe management of root-knot nematodes in tunnel grown cucumber in two districts of Balochistan, Pakistan

Shamsullah, A. Abbas^1^, M. D. Gogi^2^, S. Siddique^3^ and M. A. Ali^1^

^1^Dept. of Plant Pathology, University of Agriculture, Faisalabad-38040, Pakistan

^2^Dept. of Entomology, University of Agriculture, Faisalabad-38040, Pakistan

^3^Dept. of Entomology and Nematology, University of California Davis, Davis, CA

### Abstract

Root-knot nematodes (*Meloidogyne* spp.) are major agricultural pests causing significant yield losses in vegetable crops. Cucumber (*Cucumis sativus*) is an important vegetable that is consumed as fresh and in processed form in Pakistan. The area under cucumber cultivation in Pakistan is 3367 ha with 68664 tons of production annually. Cucumber is highly infested by root-knot nematodes (RKNs) worldwide. In Balochistan, cucumber is cultivated under tunnels. In a pilot survey, we found a huge infestation of RKNs in cucumber grown in plastic tunnels in Balochistan. However, the information on which RKN species are associated with tunnel grown cucumber in Balochistan Province is lacking. Therefore, a detailed survey was conducted to determine and document the prevalence and severity of RKNs on cucumber from the plastic tunnels in the districts of Pishin and Quetta. The study from the two districts showed differences in root-knot nematode prevalence, severity, and incidence. The higher prevalence was found in district Pishin (57.5%), whereas that in Quetta was 46.6%. The disease severity was measured in terms of the galling index, the maximum average disease severity in Pishin was (5.71), in Quetta, the maximum average disease severity was (4.73). The disease incidence of RKN in Pishin was 66%, while that in Quetta was 59%. We performed morphometric and molecular identification and characterization of different RKN isolates based on morphological characters which include (female perineal pattern, shapes and lengths of total body and stylet) and molecular identification (ITS 18S, Cox1 and Cox2 amplification). We further designed an experiment to manage the RKNs by using sole and combined application of biocontrol agents (*Bacillus subtilis* and *Trichoderma harzianum*) and biochar along with nematicide (Fosthiazate) as control under the commercial tunnel cultivation conditions. Plant growth parameters and nematode severity characteristics of cucumber were recorded. Overall, this research can identify RKN infestation in a particular region and ecosafe management approach against RKN to improve plant health and reduce the reliance on chemical nematicides.

## Anaerobic soil disinfestation for enhanced sweetpotato yield and profitability: evaluating cultivar specific responses and carbon source effectiveness

Sharma, Sanjeev^1^, K. Churamani^2^, S. Simardeep^3^, K. Puskar^1^ and C. Matthew^3^

^1^Dept. of Forest Resources and Environmental Conservation, Clemson University, Clemson, SC, 29634

^2^Dept. of Plant and Environmental Sciences, Clemson University, Clemson, SC, 29634

^3^Coastal Research and Education Center, Clemson University, Charleston, SC, 29414

### Abstract

Plant-parasitic nematodes and weeds adversely affect the quantity and quality of sweetpotato yield, and they are becoming increasingly challenging to manage in organic production which is valued at over $77 million annually in South Carolina. In response to these challenges, anaerobic soil disinfestation (ASD) has emerged as a crucial technique, offering a chemical-free solution to effectively manage the soil-borne issues and sustainably enhance crop productivity. However, economic feasibility studies of ASD are currently lacking as implementation of this technique requires more cost, labor, and monitoring and management. A field study was conducted to evaluate cultivar specific response and carbon source effectiveness to investigate the impact of ASD on plant-parasitic nematode abundance and sweetpotato production. The treatments included three carbon amendments (cotton seed meal, chicken manure plus molasses, and brassica waste) and a no carbon amendment control. Sweetpotato lines employed were ‘Bayou Belle’, ‘Murasaki, Monaco’, and ‘USDA 18-040’. Each carbon source, along with the control was replicated four times, and four cultivars were replicated sixteen times in a randomized complete block design. There were four rows with four main plots ten feet apart, and each main plot had all four cultivars five feet apart from each other. After five months, the yield from each cultivar and carbon source was harvested, weighted, and multiplied based on the current price sheet of sweetpotato grading from the South Carolina State Farmers Market. Partial budget analysis revealed that the combination of chicken manure plus molasses yielded the highest profit of $21,560 per hectare, followed by profit of $17,252 per hectare from cotton seed meal and loss of $1,819 per hectare from brassica waste, which were all compared to the controls and with ‘Bayou Belle’ cultivar. The average profit from ‘Bayou Belle’ cultivar was $23,644 per hectare, from ‘Murasaki’ cultivar was $12,692 per hectare, from ‘USDA 18-040’ cultivar was $7,679 per hectare and from ‘Monaco’ cultivar was $1,200 per hectare. The yield from the ‘Bayou Belle’ cultivar was 70,273 kg per hectare, from ‘Murasaki’ was 31,523 kg per hectare, from ‘USDA 18-040’ was 24,004 kg per hectare and from ‘Monaco’ was 36,136 kg per hectare. The abundance of plant-parasitic nematodes per 100 cm^3^ soil was 232 in plots receiving chicken manure plus molasses, 273 in plots receiving brassica waste, 718 in plots receiving cotton seed meal and 1028 in the control. Chicken manure plus molasses showed its potential to reduce nematode populations while increasing profitability of sweetpotato. These findings underscore the importance of cultivar selection and carbon source to optimize ASD performance for sweetpotato production, offering valuable guidance for farmers seeking to enhance yields and profitability while promoting soil health.

## Reklemel™ active (fluazaindolizine, salibro™) findings from the development of a novel nematicide against key plant-parasitic nematodes

Shirley, Andy^1^ T. Sunil^1^ and T. Thoden^2^

^1^Corteva Agriscience™, Indianapolis, IN

^2^Corteva Agriscience™, Munich, Germany

### Abstract

Reklemel™ active (fluazaindolizine, Salibro™) is a novel, non-fumigant, sulfonamide nematicide discovered and developed by Corteva Agriscience. It has demonstrated its ability to control economically important plant-parasitic nematode species infesting a wide range of annual and perennial crops in North America. Reklemel™ has a unique mode of action, a favorable soil mobility profile and can be used in a range of different application methods. Reklemel has a favorable environmental profile and is compatible with beneficial soil biologicals, positioning it as a key partner in an integrated nematode management program. The development of Reklemel™ has been driven by its selectivity and efficacy against economically relevant *Meloidogyne* spp., as well as other endo- and ecto-plant parasitic nematodes. The discovery, development, and characterization of this chemistry comprises over 15 years of extensive research at the global level, encompassing thousands of laboratory, greenhouse, and field studies. This developmental work has defined key attributes of this novel chemistry, including nematode activity, soil behavior, and soil health compatibility. A US-based summary of the key insights gained from the development of Reklemel™ as well as label and product registration updates will be presented.

## Precision EPN applications - infected cadavers as an optimized tool for integrated pest management

Silvester, Nicco P. and B. S. Sipes

Dept. Plant and Environmental Protection Sciences, University of Hawai'i at Mānoa, Honolulu, HI 96822

### Abstract

Hawai'i presents a unique agricultural and regulatory environment that can be both opportune and difficult for the utilization of entomopathogenic nematodes (EPN). Under Hawai'i law, importation of commercial EPN products is administratively challenging. In response, we have pursued a program that includes the use of local EPN isolates, reducing the numbers of EPN required for applications, and protecting EPNs applied in the field from harsh environmental conditions. Recommendations for application of most commercial EPN are to apply 2 billion IJ/ha at dusk using foliar sprays for above ground insect pests. These recommendations are challenging in Hawai'i as the rate is difficult to achieve without access to commercial EPNs as well as grower reluctance to apply later in the day avoiding Hawaii’s tropical sunlight and temperatures. Applying EPNs following traditional methods during the daylight hours directly exposes the IJs to UV radiation and desiccating conditions, lowering EPN survival. In the laboratory, we have found 1 IJ/cm^2^ can be effective against pests such as the cabbage moth. Laboratory experiments have also demonstrated that spray adjuvants that protect against desiccation and UV radiation increase IJ infection of insect larvae. EPN-infected cadavers protect the IJs from adverse environmental conditions, provide a grower-friendly handling method, and may also allow reduced application rates. Reducing the number of EPNs required per treatment, protecting the EPNs from desiccation and UV radiation exposure, either through adjuvants or use of infected cadavers, is paramount to providing EPN tactics that are both sustainable and adoptable for growers. We have demonstrated that it is feasible to use EPNs for insect pest control under the constraints imposed by the Hawai'i regulatory environment. EPNs should not be discounted as a tool of IPM, as they can be optimized as a precise resource conserving application method.

## Utilization of anaerobic soil disinfestation for managing nematodes and weeds in organic sweetpotato

Singh, Simardeep^1^, C. Khanal^2^ and M. Cutulle^1^

^1^Clemson University, Dept. of Plant and Environmental Sciences, Coastal Research and Education Center, Charleston, SC 29414

^2^Clemson University, Dept. of Plant and Environmental Sciences, Clemson, SC, 29634

### Abstract

Anaerobic soil disinfestation (ASD) is a promising pest management strategy and is an alternative to chemical-led approaches that has shown potential to manage soil-borne pathogens and weeds in vegetable production systems. ASD is facilitated by incorporating carbon sources into the soil, tarping the soil with plastic mulch, and irrigating to the soil saturation. To evaluate the impact of ASD on weed and nematode management in organic-grown sweetpotato, greenhouse studies were conducted at Clemson University, Clemson, South Carolina. Experiments were laid out in a randomized complete block design in 23-cm-top diameter plastic pots containing 13.8 kg soil (microcosms) with two carbon sources [ASD (soil amended with chicken manure + molasses as carbon source) and non-ASD (non-amended control)] in the main plot and twenty sweetpotato genotypes in subplots. Three-week-old seedlings of tomato (cv. Rutgers) were planted in each microcosm followed by inoculation with 10,000 eggs of the southern root-knot nematode (*Meloidogyne incognita*). ASD was initiated one month post inoculation to allow nematodes to complete one life cycle. At the time of the ASD initiation, each microcosm was also inoculated with weed seeds [yellow nutsedge (10 tubers) and carpet weed (100 seeds)]. ASD was conducted for three weeks, followed by the transplantation of sweetpotato slips after one week of ASD termination. Weed counts, abundance of nematode second stage juveniles (J2) in soil and eggs in root, and sweetpotato above and below ground biomass data were collected. Our results suggested that the microcosms receiving the carbon amendment spent the most time under anaerobic conditions (<200 mvh). The soil abundance of J2 varied among the sweetpotato lines, with the lowest number observed in sweetpotato cultivar Ruddy (23/100 cm^3^ of soil) under ASD treatment. However, the commercial cultivar Beauregard supported the greatest nematode population under non-ASD treatment (163 J2/100 cm^3^ of soil). Significantly lower nematode eggs (207/g root) were observed under ASD treatments as compared to non-ASD treatments (35,081 /g root). Employment of ASD reduced overall weed cover percentage by 78% compared to the control. Individual weed count of yellow nutsedge and carpet weed were suppressed by 75% and 70%, respectively by ASD as compared to the non-ASD treatments. Sweetpotatoes in ASD treated microcosms had significantly higher above-ground biomass (9.1 g) as compared to the non-ASD (4.2 g) while the belowground biomasses were statistically similar. The results of this study demonstrated that the ASD has the potential to managing nematode and weeds in sweetpotato production systems.

## Diversity and phylogenetics of *xiphinema* endosymbionts in North American ecosystem

Sirengo, Kihoro-David, T. Harris, K. Powers, B. Higgins, P. Mullin and T. Powers

Department of Plant Pathology, University of Nebraska, Lincoln, NE 68583

### Abstract

Research into bacterial endosymbionts, particularly those associated with plant-parasitic nematodes (PPNs), has revealed crucial ecological relationships. Endosymbionts enhance their hosts’ survival by providing vital nutrients, especially where these are scarce. Among the PPNs, the dagger nematodes of the *Xiphinema americanum* group, with approximately 60 nominal taxa, are notable for their symbiosis with intracellular bacteria *Candidatus Xiphinematobacter* species. These nematodes are of agricultural concern as they transmit nepoviruses that severely affect a wide range of crops. Identification of *X. americanum* group species is challenging due to their overlapping morphological characteristics, which complicates the differentiation of their associated endosymbionts as well. To date, the use of fluorescence in situ hybridization and molecular markers have been used to characterize *Ca. Xiphinematobacter* species. Most reports of *Ca. Xiphinematobacter* have been from *X. americanum* sensu stricto, *X. brevicolle* complex and *X. rivesi* species. Other reports of these bacterial endosymbionts are limited to less widely distributed species across the globe. A recent study has suggested synthesis of essential amino acids and vitamins for *X. americanum* as the primary function of these endosymbionts. In our study, we characterized *X. americanum* group and their endosymbionts across North American habitats, from native prairies to forests, utilizing the mitochondrial cytochrome c oxidase I (COI) gene for nematodes and 16S rRNA marker for endosymbionts. We analyzed 278 COI and 104 16S rRNA including both newly generated and GenBank sequences. Bayesian inference and maximum likelihood trees were used to assess relationships. Overall, we observed a high level of diversity within both the nematode and bacterial endosymbionts trees. In prairies and rangelands, we discovered three unique bacterial clades: one clade supported by posterior probability PP=93% was comprised of samples from Great Plains cottonwood trees (*Populus deltoides*) that was identical in sequence to *Ca. X. rivesi* reported from Spain. Other members of this clade included Nebraskan samples from horticultural chestnut trees that closely matched GenBank *Ca. Xiphinematobacter* sp. 1 from California. The second distinct clade included samples from Nebraska tallgrass prairie that groups with *Ca. X. rivesi* from California. Both clades reveal that the nematode *X. rivesi* includes multiple strains of *Ca. Xiphinematobacter*. The third clade comprised of native prairie and agricultural samples, formed a genetically tight group (PP=100%) with *Ca. X. americani* from *X. americanum* sensu stricto from across North America. To date, we lack strong evidence of phylogenetic congruence between endosymbionts and their nematode hosts. Coevolutionary studies are underway to better understand the nature of this symbiotic relationship.

## Soil health insights for soybean cyst nematode management: a case study approach

Small, Ambria and H. D. Lopez-Nicora

Dept Plant Pathology, The Ohio State University, Columbus, OH 43210

### Abstract

Soybean cyst nematode (SCN), or *Heterodera glycines*, is the most economically important pathogen of soybean in North America. Infection by SCN can result in up to 30% yield loss without any observable above-ground symptoms in soybean plants. Evidence suggests that soil texture and other edaphic factors may affect SCN population changes throughout the growing season. There is limited information regarding the relationship between SCN population dynamics and indicators of soil health, including nutrient recycling and aggregate stability. This study’s main object is to quantify the relationship between SCN population dynamics and soil health status during the soybean growing season in Ohio. Specifically, the study will investigate the influence of soil health parameters (soil aggregate ratio, organic carbon content, nutrient profile), texture, and management practices in the proliferation and suppression of SCN populations. The intended impact of this study is to contribute case study data to the development of integrated soil management practices to reduce SCN populations and optimize soybean production in a sustainable approach. Samples were submitted during spring and postharvest timepoints. This will allow us to assess how these soil characteristics interact to affect SCN survival and reproduction. In 2023, 112 Ohio agronomic fields were sampled in the spring. Field sites generally follow the corn-soybean rotation, but this study also allowed for locations that grow wheat and forage. Seventy-nine fields were sampled post-harvest. Reproduction factor (RF = SCN post-harvest / SCN spring) could be calculated for fields that were sampled in both spring and post-harvest (N = 66), and ultimately represented 16 of the 88 Ohio counties. The average RF for the 2023 growing season was 307, with a spread of 0 to 15,560. Of these sites, 48% witnessed either a sustained or increased SCN population for the 2023 growing season, and 25% had no detection of SCN. Soil health and texture data were collected by an independent laboratory. Additionally, field management information was collected from each site. Management strategies, such as crop rotation, cover cropping, manure application, and tilling practices have a direct influence on soil chemical profiles and structure. Preliminary analysis from the 2023 dataset showed that SCN reproductive factor was not influenced by individual or combined effect of historical tilling, cover-cropping, or manure fertilization practices (*P* > 0.05). Data collection for post-harvest soil nutrient, texture, and soil health parameters are ongoing and will be analyzed for relationships with RF values.

## Impact of organically managed perennial Kernza on nematode community composition and food web indices

Smychkovich, Alexandra^1^, S. Tajik^2^, L. Deiss^2^, S. Culman^3^ and C. Sprunger^1^

^1^W.K. Kellogg Biological Station, Dept. of Plant, Soil and Microbial Sciences, Michigan State University, Lansing, MI 49060

^2^School of Environment and Natural Resources, The Ohio State University, Wooster, OH, 44691

^3^Dept. of Crop and Soil Sciences, Washington State University, Pullman, WA, 99164

### Abstract

Kernza (*Thinopyrum intermedium*), a perennial grain with an extensive fibrous root system, is emerging as a climate-smart alternative to annual wheat with the potential to improve soil health and increase the agronomic efficiency of grain production. Furthermore, Kernza can be planted as a dual-purpose crop that can be used as a forage source following grain harvest. The specific soil health benefits, best management practices, and impacts of Kernza production on soil biology are still being quantified. The objectives of this study were to 1) evaluate the impact of forage harvests (aboveground biomass removal) at varying frequencies on nematode community composition and food web indices (maturity index, enrichment index, structure index, channel index), and 2) quantify relationships between nematode indices and PLFA biomarkers in an organically managed, dual-purpose Kernza cropping system. Replicated experiments (RCBD) were implemented at 5 sites across the United States, located in Minnesota, New York, Kansas, and Ohio. Harvest removal did not have an impact on soil food webs (*p* > 0.05), demonstrating that intensive forage removal has little negative impact on soil health. Nematode community composition drastically shifted by site (*p* < 0.05), which illustrates how soil type and texture largely influence soil food web dynamics. Nematode trophic composition and food web indices – in particular, structure index - demonstrated significant relationships with PLFA biomarkers across sites. These results allow for higher resolution interpretations of biological soil health and demonstrate strong potential for nematode indices to be integrated into the soil health framework.

## Exploring miRNA dynamics in the soybean cyst nematode as a first step towards unraveling potential cross-kingdom interactions

Ste-Croix, Dave T.^1^, R. R. Bélanger^2^ and B. Mimee^1^

^1^Saint-Jean-sur-Richelieu Research and Development Center, Agriculture and Agri-Food Canada

^2^Département de Phytologie, Université Laval, Québec, Canada

### Abstract

The soybean cyst nematode (SCN), *Heterodera glycines*, represents the most significant threat to soybean cultivation in North America, causing annual losses exceeding 1.3 billion U.S. dollars. This nematode employs a sophisticated strategy, involving the secretion of various stylet-associated effector proteins, to transform soybean root tissues into a highly active feeding structure known as the syncytium. Precise control of effector expression is believed to be pivotal for the successful formation of this syncytium. So far, there have been numerous proposed mechanisms to control the expression of these proteins, among which post-transcriptional regulation stands out as a prominent example. In particular, microRNAs (miRNAs), which usually comprise non-coding RNA molecules ranging from 20 to 22 nucleotides in length, are increasingly recognized as a crucial group of native gene regulators functioning post-transcriptionally. Operating at this level, these small RNAs execute their function by binding to the 3′ untranslated regions of genes, resulting in either cleavage or transcriptional repression of corresponding mRNAs. Of note, there exists a hypothesis proposing that these small RNAs could potentially cross kingdom boundaries and modulate genes in diverse species, a concept termed cross-kingdom interaction. In this study, we embarked on characterizing the miRNAome of SCN. This involved sequencing small RNAs extracted from entire nematodes and exosomes representing different developmental stages. Our research resulted in the detection of 121 miRNA loci spanning 96 distinct miRNA families. Intriguingly, more than 75% of the newly uncovered miRNAs showed evolutionary connections within the *Heteroderidae* family, with the remaining newly identified candidates being unique to SCN. Moreover, utilizing a blend of miRNA target prediction tools customized for both plants and animals, we compiled a first repertoire of miRNA:mRNA interaction partners within the nematode and its host plant. Additionally, we identified a set of nine potential candidates for cross-kingdom miRNA regulation.

## Development of *Rotylenchulus reniformis* differs on Resistant *gossypium arboreum* lines a2-354 and a2-690

Stetina, Salliana R.

USDA Agricultural Research Service, Crop Genetics Research Unit, Stoneville, MS 38776

### Abstract

*Gossypium arboreum* lines A2-354 and A2-690 were previously reported to be resistant to the reniform nematode (*Rotylenchulus reniformis*). In two repeated growth chamber experiments, with susceptible *G. hirsutum* cultivar Deltapine 16 as a control, the effects of these lines on reniform nematode development and fecundity were examined. Genotypes were compared based on the number of nematodes on plant roots in four stages of development (vermiform, swelling, reniform, gravid) at 2-day intervals from 4 to 8 days after inoculation (DAI), and at 5-day intervals from 10 to 30 DAI. Egg production by individual females parasitizing each genotype was measured at 25 and 30 DAI. Throughout the experiment, fewer nematodes developed on the two *G. arboreum* lines than on the susceptible *G. hirsutu*m cultivar, confirming earlier reports of resistance. Progression to the gravid stage of development occurred first on the susceptible genotype Deltapine 16 (6 DAI), followed by A2-690 (8 DAI), and finally A2-354 (10 DAI). The rate of progression from the swelling to the reniform stage of development differed among the genotypes. Most individuals in the root-associated nematode population were classified in the reniform stage of development at 8 DAI for Deltapine 16 and A2-354, but this wasn’t true for A2-690 until 15 DAI. Progression from the reniform to the gravid stage of development was more synchronous, with most of the nematode population on all three genotypes classified in this stage at 25 DAI. There were no consistent significant differences in egg production by nematodes infecting the three genotypes. In the first test, there were no differences among the genotypes at 25 DAI, but Deltapine 16 produced more eggs per female (46.6) than A2-690 (28.9) at 30 DAI. In this test, there were no gravid females observed in the subset of A2-354 plants harvested at 30 DAI, so no egg counts were possible for this line. However, in the second test all three genotypes had gravid females at both 25 and 30 DAI, and A2-690 supported production of the most eggs per female (33.4) at 25 DAI. Deltapine 16 and A2-354 produced fewer eggs per female at 25.9 and 23.0, respectively, although these two genotypes did not differ from each other. No significant differences in egg production occurred at 30 DAI in the second test. The slower development could work in conjunction with reduced numbers of infections to help suppress the reniform nematode population. This study is the first report of delayed development associated with *G. arboreu*m lines A2-354 and A2-690.

## Group dynamics of entomopathogenic nematodes: followers, joiners and leaders

Stevens, Glen^1^, D. Shapiro-Ilan^2^ and E. Lewis^1^

^1^Dept. Entomology, Plant Pathology and Nematology, University of Idaho, Moscow ID, 84844

^2^USDA-ARS, SEFTNRL, Byron, GA 31008

### Abstract

Animals form groups to accomplish actions that singletons cannot. Entomopathogenic nematodes (EPNs) are generally found in groups in the field, whether in natural populations or soon after application for pest management. EPN infective juveniles (IJs) do little more than finding a host, but only in rare circumstances can an individual establish a successful infection. Thus, traveling, and then infecting, in groups makes evolutionary sense. We have documented several instances of group behaviors by EPNs. EPNs exhibit trail-following behaviors that are species-specific, and individual IJs are more likely to be followed after they have had contact with hosts. IJs disperse in an aggregated manner in the absence of environmental cues and disperse differently when under single-species vs. mixed-species conditions. IJs also are attracted to remote conspecific groups of IJs, and groups that have been exposed to hosts are more attractive than unexposed groups. Modelling group infection dynamics of EPNs suggests that infections are initiated by a small number of IJs in a group that are “risk-prone” and then followed by other IJs once the risks associated with infecting a healthy host are mitigated. Recent research also shows that those IJs that tend to disperse more are also more likely to infect hosts. All of these aspects of group behaviors require a degree of communication amongst EPN IJs, so that like pods of whales, packs of wolves and murders of crows, EPNs accomplish more in groups than could an individual.

## Impacts of root-knot nematodes (*Meloidogyne chitwoodi* and *M. hapla*) on potato yield and quality in the Pacific Northwest

Studebaker, Gabrielle^1,2^, I. Zasada^1,2^ and S. Sathuvalli^3^

^1^Oregon State University, Dept. of Botany and Plant Pathology, Corvallis, OR 97331

^2^USDA-ARS, HCRPMRU, Corvallis, OR 97331

^3^Oregon State University, Hermiston Agricultural Research and Extension Center, Hermiston, OR 97838

### Abstract

The root-knot nematodes *Meloidogyne hapla* and *M. chitwood*i are important pests in the potato production regions of the Pacific Northwest. *M. chitwoodi* is known to cause galling and blemishes on the tuber flesh, resulting in crop rejection. Given the low tolerance to these nematodes in the potato industry, our work aims to understand the relationship between tuber quality and yield and initial nematode density in soil. A fumigated field was inoculated with three initial densities of either *M. hapla* or *M. chitwoodi* (0, 50, and 200 eggs/250 cc of soil) on three varieties of potato (‘Russet Burbank’, ‘Ranger Russet’, and ‘Clearwater Russet’). At harvest, tubers were sorted and assessed for weight, total number, and external and internal quality. External quality assessed shape uniformity, growth cracks, galls, and knobs, Internal quality assessed tubers for hollow heart, brown center, internal brown spot, black spot bruise, vascular discoloration, and translucent end. A subset (n = 10) from each treatment group was peeled and graded for damage caused by *M. chitwoodi* and *M. hapla*. Our results, based on one year of data, indicate that *M. chitwoodi* reduced yield of ‘Clearwater Russet’ by approximately 40% at the highest initial density compared to the non-inoculated control (*P* = 0.025). *M. chitwood*i also cause increased severity of galling at high initial population densities. Further, it was observed that ‘Ranger Russet’ when inoculated with the highest initial density of *M. hapla* was more likely to have greater than 20 nematode infections in a tuber compared to the other varieties. Overall, *M. hapla* had no impact on total yield but we found that ‘Ranger Russet’ had reduced yield and tuber numbers in size classes below 6 ounces compared to ‘Clearwater Russet’ and ‘Russet Burbank’. This trial will be repeated with a broader range of initial densities. Results from this research will allow for a better understanding of the relationship between root-knot nematode densities in soil and their impacts on tuber yield and quality in the region.

## Entomopathogenic nematodes and entomopathogenic fungi as a potential biological control of spotted-wing drosophila (*Drosophila suzukii*)

Thapa, Rambika, M. Usman, R. Isaacs and M. Quintanilla

Michigan State University, Dept. of Entomology, East Lansing, MI 48823

### Abstract

Spotted-wing drosophila is an invasive pest that has caused significant yield loss to small fruit cropping systems in North America and Europe since its introduction in 2008. Its preference for soft-skinned fruits and polyphagous nature have led to significant yield loss. Larval feeding causes considerable damage and increases susceptibility to secondary infection by pathogenic fungi and bacteria. Management of SWD is complex due to its short generation time of only 2–3 weeks and high reproduction rate. Currently, insecticides are the primary control for SWD, but resistance development is a concern. Biological-based pest management using insect-pathogenic nematodes and fungi could be a promising alternative to conventional chemical insecticides. We conducted a study to evaluate the effectiveness of nine different entomopathogenic nematodes (EPNs) and three different entomopathogenic fungi in controlling soil-dwelling stages of SWD. The objective was to assess the strength of different strains of EPNs against larval and pupal stages of SWD and to explore the effectiveness of EPFs against soil-dwelling stages of SWD. The experiment was conducted in a completely randomized design with three replications, which were repeated three times. The bioassay arenas comprised transparent plastic cups (1 oz) with 20g of play sand. The initial moisture content of the soil was 0%, and it was air-dried at room temperature before autoclaving at 121°C for 45 minutes. To achieve a final soil moisture content of 10%, 1 ml of a solution containing four different concentrations of infective juveniles (IJs) of EPN (25 IJ/cm^2^, 50 IJ/cm^2^, 75 IJ/cm^2^, 100IJ/cm^2^) was piped onto the soil surface with 1 ml of distilled water. The control group received 2 ml of distilled water only. For the larval assay, ten 3^rd^ instar larvae were introduced, and for the pupal assay, ten pupae were added. After 8 days, the number of emerging adults was counted. A similar procedure was followed for entomopathogenic fungi, and three different concentrations of fungi were used to evaluate their efficiency. After testing several fungi and nematodes, two of the most promising were selected for further experimentation. They were then applied separately or in combination against late instar larvae and young pupae to assess their combined efficacy. While entomopathogenic nematodes and fungi may not provide 100% control of SWD populations, they could be a valuable addition to the IPM toolbox. However, another season of fieldwork is required to determine the impact of EPNs on SWD population dynamics.

## The compatibility of Salibro™ with non-target nematode species of different feeding habits

Thapa, Sita^1^ and T. Thoden^2^

^1^Corteva Agriscience^TM^, Indiana, IN 46268.

^2^Corteva Agriscience^TM^, 81677 München, Germany

### Abstract

Salibro™ (Reklemel™ active, fluazaindolizone) is a novel, non-fumigant, chemical nematicide developed by Corteva Agriscience. It has excellent activity on root-knot nematodes and in previous studies has shown to be gentle to various non-target nematode species. Approximately 30 thousand nematodes have been described, of which most of them have an important role in soil health and sustainability. The main objective of this project was to extend our understanding on the compatibility of Salibro with additional species and feeding behaviors of non-target nematode species in comparison to other commercially available nematicides. Three species of bacterial feeders (*Acrobeloides butschlii*, *Mesorhabditis spiculigera* and *Panagrellus redivivus*); one fungal feeder (*Aphelenchus avenae*), one predator (*Mononchus aquaticus*) and two entomopathogenic nematodes (EPNs), *Heterohabditis bacteriophora* and *Steinernema feltiae*, were studied. Novel data from various testing methods (*in vitro* vitality studies, horizontal movement studies, etc.) will be shared to confirm the overall gentleness of Salibro to non-target beneficial nematodes.

## Field evaluation of organic composts and nematicides against hop cyst nematode, *Heterodera humuli* in hop

Usman, Muhammad^1^, E. Darling^1^, L. Forsberg^1^, H. Chung^1^, C. Malmstrom^2^, D. Shapiro-Ilan^3^ and M. Quintanilla-Tornel^1^

^1^Department of Entomology, Michigan State University, East Lansing, 48824, MI

^2^Department of Plant Biology, Michigan State University, East Lansing, 48824, MI

^3^USDA-ARS, Southeastern Fruit and Tree Nut Research Station, Byron, 31008, GA

### Abstract

Hop cyst nematode (HCN), *Heterodera humuli* is one of the most important nematode pests of hop. It is unfortunate that currently growers have no available options to manage HCN in hop. The objective of this study was to assess suppressive efficacy of organic composts and nematicides against HCN. The trial was conducted at the Michigan Hop Alliance. We selected two adjacent rows of variety cascade for the trial because we found an average of 53 cysts per 100 cm^3^ of soil (unpublished data). For treatment application, six different plants were selected (three plants in row one and three plants in row two) and one plant was left untreated in each row as a buffer between treatments. We applied the following treatments in a randomized complete block design with five replications; treatment 1: layer ash blend (12 pounds per plot), treatment 2: poultry manure (6 pounds per plot), treatment 3: urea (0.5 pound per plot), treatment 4: Majestene (182 ml per plot), treatment 5: half rate of Velum Prime (0.5 ml per plot), and treatment 6: full rate of Velum Prime (1 ml per plot), and untreated control. To assess efficacy, we collected soil samples at three-time intervals, (1) pre-treatment application, (2) mid-season (40 days of post treatment application), and (3) end season (40 days of post mid-season sampling). At the end of the season, we selected two random plants at the height of five feet and removed all cones manually by hand in one feet area and determined their weight with weight balance. Nematodes were extracted using modified centrifugation-flotation technique from 100 cc of soil and the number of HCN juveniles, cysts, and beneficial nematodes were determined. At pre-treatment application, there were no significant differences among the treatments in HCN juveniles, cyst, and beneficial nematodes. At the mid-season sampling, there is a significant difference between treatments in HCN juveniles (*P* = 0.07) and beneficial nematodes (*P* = 0.008), but non-significant difference observed against cysts. The maximum suppressive efficacy was exhibited by treatment 5 against juveniles and cysts while the lowest was observed in treatment 3 and treatment 4 against juveniles and cysts respectively compared with the untreated control. At the end of the season, relatively lowered HCN juveniles and cysts were observed in treatment 5 and 6 respectively compared with untreated control. Regarding cones yield, maximum yield (145.2±18.9 g) observed in treatment 6 compared with untreated control plot (90.2±13.7 g). Results from this study revealed that using composts and nematicides for suppression of HCN in hop may be efficacious, but more research is needed before concrete recommendations can be made.

## A two-year survey for presence of plant-parasitic nematodes in hopyards of Midwest

Usman, Muhammad^1^, E. Darling^1^, A. Palmisano^1^, L. Núñez-Rodríguez^2^, I. A. Zasada^2^, H. Chung^1^, D. Shapiro-Ilan^3^ and M. Quintanilla-Tornel^1^

^1^Dept. Entomology, Michigan State University, East Lansing, 48823, MI

^2^Dept. Botany and Plant Pathology, Oregon State University, Corvallis, 97331, OR

^3^USDA-ARS, Southeastern Fruit and Tree Nut Research Station, Byron, 31008, GA

### Abstract

Hop is an important crop in the United States with a total cultivated area of about 55,710 acres in 2023. The Pacific Northwest including Washington, Idaho, and Oregon are major producing areas, producing more than 97.5% of total U.S. hop. In 2023, 475 acres were harvested from the Midwest (Michigan, Ohio, and Minnesota). Among different limiting factors, the plant-parasitic nematodes (PPNs) are also responsible for yield reduction in hop. Knowledge on abundance and distribution of PPNs is very important for effective management strategies for better hop production. To date, literature is lacking in the abundance and distribution of PPNs in hop yards of the Midwest (Michigan, Ohio, and Minnesota). The objective of this study was to determine the abundance and distribution of PPNs in the Midwest. To accomplish our goal, we designed two-year surveys of hop yards of the Midwest in the summer of 2021 and fall, 2022. In 2021, 28 fields were sampled across Michigan with seven samples collected from each field (n=196). In 2022, we extended our survey to Minnesota and Ohio along with Michigan and visited 85 fields with two samples collected from each field (n=170). Samples were collected close to the base of hop plants with soil sampler in a W shape pattern. Nematodes were extracted from 100 cm^3^ of soil by modified centrifugation-flotation technique and identified and quantified with an inverted microscope using 20× lens. All PPNs were identified at genus level except HCN identified at species level with aids of morphological and molecular tools. For both years, hops fields were found positive with root lesion (*Pratylenchus* spp.), American dagger (*Xiphinema* spp.), hop cyst (*Heterodera humuli*), root knot (*Meloidogyne* spp.), spiral (*Helicotylenchus* spp.), stunt (*Tylenchorhynchus* spp.), ring (*Mesocriconema* spp.), stubby root (*Trichodorus* spp.), lance (*Hoplolaimus* spp.), and pin (*Paratylenchus* spp.) nematodes. Among economically important PPNs, root lesion was found to be the most abundant nematode with positive fields (96.2% and 86%), followed by American dagger (86% and 87%), hop cyst (50% and 35.3%) and root knot (10.71% and 24.7%) in 2021 and 2022 respectively. The highest numbers were recorded for root lesion (46 and 304), dagger (258 and 205), hop cyst (190 and 338), and root knot (204 and 224) nematodes in 100 cm^3^ of soil in 2021 and 2022 respectively. The results from these surveys concluded that hops of the Midwest were found to be infested with economically important PPNs, and further research is required to idnetify these PPNs on a species level using molecular tools. Increasing our knowledge of these pests will help growers to mitigate the effect of PPNs on hop production via effective management strategies.

## History, distribution and regulatory impact of the peach root-knot nematode, *Meloidogyne floridensis*, in Florida, USA

Vau, Silvia, M. R. Moore and J. A. Brito

Florida Department of Agriculture and Consumer Services, Division of Plant Industry, Gainesville, FL 32608

### Abstract

The peach root-knot nematode, *Meloidogyne floridensi*s (Handoo et al, 2004), is known to occur only in the USA. It was first found in 1991 in Gainesville, FL infecting root-knot nematode resistant peach (*Prunus persica*) rootstock ‘Nemared’ and ‘Guardian’. Later in 1996, it was also reported infecting two other root-knot nematode resistant peach rootstocks, ‘Nemaguard’ and ‘Okinawa’. In addition to the Gainesville isolates, seven additional isolates of *M. floridensis* have been identified in seven Florida counties on different host plants: snap bean (*Phaseolus* sp.) in Alachua County, lilac tassel flower (*Emilia sonchifolia*), and tomato (*Solanum lycopersicon*) in Hendry County, eggplant (*Solanum melongena*) in Hillsborough County, and tomato in Charlotte, Indian River, Seminole, and St. Lucie Counties. The importance regarding *M. floridensis* as a pathogen of regulatory importance is due to the ability this nematode has to overcome root-knot nematode resistance genes. This nematode is presently on the list of nematodes regulated in the state of California for the ornamental nursery certification program. All plant material exported to California should be free of this nematode in agreement with the QC-390 Master permit for the shipment of nursery stock from Florida to California.

## First report of sting nematode (*Belonolaimus longicaudatus*) in Maryland

Waldo, Benjamin^1^, Z. Handoo^1^, A. Skantar^1^, A. Habteweld^1^, S. Li^1^ and F. Shahoveisi^2^

^1^USDA-ARS, Mycology and Nematology Genetic Diversity and Biology Laboratory, Beltsville, MD 20705

^2^University of Maryland, Dept. of Plant Science and Landscape Architecture, College Park, MD 20742

### Abstract

A soil sample collected in July 2023 from an athletic field in Baltimore County, Maryland part of a turfgrass nematode survey, contained *Belonolaimus longicaudatus*. The density was 4 individuals per 100 cm^3^ soil, and no visual symptoms were observed in the bermudagrass field. Morphological features and morphometrics of males and females were consistent with *B. longicaudatus* and placed the Maryland population in a subclade that was geographically represented by populations from north and west Florida, Texas, and South Carolina. Sequencing of the internal transcribed spacer region ITS1 and 2 and 28S large ribosomal subunit D2-D3 expansion region confirmed the species identity. Phylogenetic trees and parsimony network analysis placed the Maryland isolate in a large grouping of *B. longicaudatus* populations including those from Alabama, Delaware, Florida, Indiana, Mississippi, South Carolina, and Texas. To our knowledge, this is the first detection of *B. longicaudatus* in Maryland.

## From discovery to development of host resistance: the story of *Meloidogyne enterolobii* in Louisiana

Watson, Tristan T., D. Galo and J. S. Rezende

Dept. Plant Pathology and Crop Physiology, LSU AgCenter, Baton Rouge, LA 70803

### Abstract

*Meloidogyne enterolobii* is an emerging root-knot nematode species in Southeastern United States and is particularly damaging in sweetpotato production due to the aesthetic damage caused to the marketable product. In Louisiana, *M. enterolobii* was first intercepted in 2018 on a shipment of contaminated sweetpotato storage roots. A second interception on storage roots in 2020 resulted in a state ban on importing sweetpotato planting material from out-of-state. This nematode species has subsequently been intercepted entering Louisiana on imported ornamental and nursery plants, suggesting a second major dissemination pathway also exists. In response to this threat, nematode management strategies for this new species are actively being researched. The sensitivity of *M. enterolobii* to new and existing nematicides has been evaluated using *in vitro* laboratory and potted greenhouse experiments, and our data suggest that nematicides that are effective against the common southern root-knot nematode (*Meloidogyne incognita*) will also work on *M. enterolobii*. The host status of rotation crops grown in Louisiana to *M. enterolobii* has been evaluated, and although many crops were susceptible, corn and grain sorghum supported minimal nematode reproduction and have been successfully implemented as rotation crops in two production fields contaminated with *M. enterolobii*. Many of the currently grown commercial sweetpotato varieties in the United States are highly susceptible to *M. enterolobii*; however, breeding lines in the LSU AgCenter Sweetpotato Breeding Program have shown a high level of resistance. A time-course nematode development study was conducted to further characterize the mechanism of resistance in select genotypes, and our data suggest that *M. enterolobii* resistance in sweetpotato is associated with a hypersensitive response that prevents the establishment of nematode feeding sites. Commercial release of sweetpotato varieties with combined resistance to *M. enterolobii* and *M. incognita* is forthcoming; however, the utility of deploying host resistance for *M. enterolobii* management in sweetpotato remains unknown.

## Managing plant-parasitic nematodes in grape using combinations of pre-and post-plant nematicide applications

Westphal, Andreas, T. Buzo and Z. T. Z. Maung

University of California Riverside, Dept. of Nematology, Parlier, CA 93648

### Abstract

California vineyards are often infested by different assemblages of plant-parasitic nematodes. The extended lifespan of vineyards resulting in longtime exposure of grape roots with defined nematode susceptibilities possibly contributes to concomitant infestations of multiple species including *Meloidogyne* spp., *Mesocriconema xenoplax*, *Tylenchulus semipenetrans, Paratylenchus hamatus, Xiphinema americanum and X. index*. Preplant soil fumigation with 1,3-dichloropropene-containing materials (1,3-D), e.g., Telone, can reduce initial population densities, thereby delaying significant nematode damage. However, recently modified application protocols impede the economic feasibility and efficacy of preplant soil fumigation, so alternative nematode management strategies are needed. In a ‘Sauvignon Blanc’ wine grape (*Vitis vinifer*a L.) vineyard with *Paratylenchus* sp., preplant drench applications of two low-volume materials (fluensulfone, Nimitz: 3.5 or 7.0 L/ha; fluazaindolizine, Salibro: 9 L/ha) and one experimental high-volume material (468 L/ha) were tested along with a non-treated control and a Telone EC treatment (224.5 L/ha). All plots received 150 L/m^2^ drench water via a drip irrigation system spiked with the respective treatment material. In the following years, treatments of fluensulfone and fluazaindolizine were followed by the respective postplant applications. The high-volume and 1,3-D treatments were treated with spirotetramat (Movento: 457 ml/ha) spray applications. In this trial, the high nematicide intensity in the 1,3-D and high-volume treatments plus the postplant application of Movento resulted in improved plant growth compared to the non-treated control. In a second experiment with wine grape ‘Chardonnay’ in the presence of *Meloidogyne* sp., *M. xenoplax*, *T. semipenetrans, Paratylenchus* sp., and *X. americanum*, four preplant soil treatments received fluazaindolizine (Salibro) at 9 L/ha in 150 L/m^2^ drench water. Controls of non-treated or Telone EC at 328 L/ha were included as controls. A pulsed 9 L/ha application of fluazaindolizine delivered in 60 L/m^2^, followed by 60 L/m^2^ and 30 L/m^2^ after initial treatment was also included. Beginning in the second year, postplant applications of 9, 4.5, 2.24 or 1.13 L/ha Salibro were made in plots following the 9-L fluazaindolizine (Salibro) preplant soil drench. Management programs had strong effects on plant growth. In the second year in several of the preplant treatments, central trunks and in some treatments, cordons on the single-wire system were trained. In the controls, vines were too weak to be trained up the trellis. In the third growth year, total sugar yields (fruit fresh weight × % soluble solids) after 1,3-D and preplant Salibro drench plus 2.25 L/ha as postplant treatment were higher than in the non-treated control. In the fourth year, total sugar yields were higher than in the non-treated control in all treatments, not significantly different from the 1,3-D treatment except in the pulsed preplant treatment. Combining 1,3-D or the experimental high-volume material with Movento or the use of fluazaindolizine as pre-and postplant remedy was beneficial in increasing plant growth and yield. This suggested that less material amount-demanding nematode management strategies can be identified for grape production.

## Developing strategies for sustainable walnut production during times of dwindling management tools

Westphal, Andreas^1^, Z. T. Z. Maung^1^, T. Buzo^1^, G. T. Browne^2^, D. A. Kluepfel^2^, M. Nouri^3^, B. Holtz^3^, C. A. Leslie^4^ and P. J. Brown^4^

^1^Dept. of Nematology, University of California Riverside, Parlier, CA 93648

^2^USDA-ARS, Crops Pathology and Genetics Research Unit, Davis, CA 95616

^3^UCANR, San Joaquim County, Stockton, CA, 95206

^4^Dept. Plant Sciences, University of California Davis, Davis, CA 95616

### Abstract

Edible walnuts are a crucial agricultural commodity in California. On the deep-rooting and fertile soils of the Central Valley, soil-borne pathogens, including *Pratylenchus vulnus, Meloidogyne* spp., *Agrobacterium tumefaciens* (crown gall, CG), and *Phytophthora* spp. (Phytophthora root and crown rot, PHY) threaten sustainable productivity. Damage by the wide-spread *P. vulnus* has been mitigated by the application of preplant soil fumigation with 1,3-dichloropropene. Recent increased regulatory stringency plus expected further restrictions encumber this tool. For the development of walnut rootstocks with reduced susceptibility and sensitivity to *P. vulnus* and other soil-borne pathogens, a consortium of plant scientists, plant pathologists, breeders, molecular biologists, and farm advisors has formed. To phenotype two breeding populations of *Juglans microcarpa* × *J. regia* ‘Serr’, clonal plants of ~300 accessions each were produced from tissue culture and planted to nematode-infested field plots or tested for CG and PHY susceptibilities. In three accessions, the favorable trait of some nematode resistance and tolerance was coupled with reduced susceptibility to the other two pathogens. These three accessions grew vigorously under nematode-infested conditions. Comprehensive testing in field trials with grafted trees is ongoing before their commercial use can be considered. For the development of alternative preplant soil treatment protocols, soil drenches with non-fumigant nematicides (i) allyl-isothiocyanate (AITC) used at high volumes, (ii) anaerobic soil disinfestation (ASD) at adjusted watering schedules, and (iii) drenches with fluazaindolizine to some extents were identified as useful soil treatments. With contemporary rootstocks, the combination of these pre-plant treatments with post-plant remedies was necessary. The planting of selected experimental rootstocks with favorable nematode response to plots of different preplant treatments demonstrated potential benefits of this strategy when fostering the sustainability of walnut production.

## Model-mania! exploring differences in soil health management between four tropical cover crops in a sweetpotato agroecosystem

Wiseman, Benjamin, M. Pitiki and K. -H. Wang

Dept. Plant and Environmental Protection Sciences, University of Hawaii at Manoa, Honolulu, HI 96822

### Abstract

Soil disruption inherent to sweetpotato bed formation and harvesting potentially damages soil health. Multiple soil health parameters such as nematode community indices, microbial phospholipid fatty acid analysis (PLFA), and soil physical and chemical properties were measured during two cover crop-sweetpotato cropping cycles to better understand the effect of cover crops on promoting soil health and suppressing nematodes in a sweetpotato agroecosystem. Four cover crops were examined: marigold, sorghum, sunn hemp, and velvet bean. Measurements were analyzed using a mixed model with cover crop and period as fixed effects and plot as a random effect. In Cycle I, plots with velvet bean (VB) had higher soil carbon, and plots with either VB or sunn hemp (SH) had higher ammonia nitrogen compared to bare ground (BG). Compared to BG, at termination of Cycle I (5 months after sweetpotato planting), VB was the only treatment that increased various soil health indicators including nematode and microbial PLFA diversity, Gram negative (G−) bacteria, arbuscular mycorrhizal fungi (AMF) and total fungal biomass (*P* < 0.05). At the end of Cycle II, VB and sorghum (SG) increased G−, AMF, total fungal biomass but only VB increased abundance of bacterivorous nematodes compared to BG (*P* < 0.05). Data were then subjected to partial redundancy analysis, PERMANOVA, and canonical correspondence analysis (CCA) to compare various models of analyzing soil health, sweetpotato yield, and plant-parasitic nematode suppression. In Cycle I, at mid-season of the sweetpotato crop, reniform nematode number was negatively related to G+/G− bacteria ratio and only VB was segregated from BG in the CCA scatter plot (first two Canonical axes=77.8% variation). However, towards the end of Cycle I, abundance of reniform nematodes was negatively related to nematode diversity, bacterivorous nematode numbers, AMF, and fungi/bacteria ratio (F/B), whereas sweetpotato yield was enhanced by nematode enrichment index (EI) and structure index (SI). At this time, VB and SH were most segregated from BG in the CCA scatter plot (axes=99.87% variation). Progressing to Cycle II following soil disruption from sweetpotato harvesting and bed formation, CCA depicted a strong negative relationship between reniform nematode abundance and SI, nematode diversity, microbial soil respiration, and cover crop biomass. VB, SG, and SH were all segregated from BG in the CCA scatter plot (68.99% variation). Towards mid-season, abundance of reniform nematodes was negatively related to total microbial PLFA, and G+, and G− biomass. Abundance of predatory nematodes, SI, protozoa, AMF, and G− bacteria, along with microbial respiration rate, cover crop biomass, and soil water infiltration rate all showed positive relations with sweetpotato yield. At this point, VB and SG continued to segregate from BG in the CCA scatter plot (90.02% variation). The various statistical techniques bring different perspectives to data modeling with unique strengths and shortcomings. None-the-less, this study showed gradual progress of cover crop benefits on soil health over time.

## Organic approaches to manage sweet potato weevil (*Cylas formicarius*) using entomopathogenic nematodes and entomopathogenic fungi in Hawaii

Wong, Landon, K. -H Wang, and B. S. Sipes

Dept. Plant and Environmental Protection Sciences, University of Hawaii, Honolulu, HI, 96822

### Abstract

Sweet potato weevil (SPW, *Cylas formicarius*) is a key pest on sweet potato in Hawaii. The objective of this research was to evaluate the efficacy of 3 species of entomopathogenic nematode (EPN) from Hawaii, 3 strains of entomopathogenic fungi (EPF) against SPW. *In vitro* screenings on SPW larvae demonstrated *Steinernema feltiae* MG-14, *Oscheius tipulae* OA-12, and *Heterorhabditis indica* OM-160 caused 60%, 30% and 30% SPW mortality, respectively. Another *in vitro* assay examining efficacy of the EPF Botanigard^®^ (a.i. *Beauveria bassiana* GHA strain) and Met Master^®^ (a.i. *Metarhizium anisopliae*) revealed 30% and no mortality on SPW larvae compared to the untreated control, respectively. Six field trials were conducted to evaluate the efficacy of EPN and EPF. Four field trials were conducted to evaluate EPF. Botanigard^®^ and Met Master^®^ did not reduce SPW population densities or SPW damage. However, an indigenous *Metarhizium* sp. isolated from Koko Head, Hawaii (KO-002) amended into mill run and incorporated into the soil at sweet potato planting reduced SPW damage (*P* ≤ 0.05), reduced SPW numbers inside the sweet potato tuberous roots (*P* ≤ 0.05), and improved plant vigor (P ≤ 0.05) compared to unamended soil. Two of the field trials evaluating EPNs showed that *S. feltiae* MG-14 and *O. tipulae* OA-12 reduced SPW population densities (*P* ≤ 0.05) in sweet potato tuberous roots. When *S. feltiae* and Met Master^®^ were applied together, the effectiveness of *S. feltiae* was reduced. Literature suggests that volatile organic compounds in *Metarhzium* may reduce infectivity of EPN. Overall, commercial EPF was ineffective in suppressing SPW population densities in the field, but the local isolate *Metarhizium* KO-002 did reduce SPW population and damage. Therefore, for organic management of SPW, integrating EPN or *Metarhizium* KO-002 with pheromone traps can be effective at controlling SPW.

## Monitoring fluazaindolizine efficacy using qPCR in *Meloidogyne hapla*

Wram, Catherine^1^ and I. A. Zasada^2^

^1^USDA-ARS, Mycology and Nematology Genetic Diversity and Biology Laboratory, Beltsville, MD 20705

^2^USDA-ARS Horticultural Crops Disease and Pest Management Research Unit, Corvallis, OR 97331

### Abstract

*Meloidogyne hapla* is present in 26 states in the United States and is an important pest of carrot, alfalfa, sugar beets, strawberries, potatoes, and grapes. Chemical controls are the most widespread means of managing this pest, however, there has been a reduction in available nematicides. Ensuring longevity of limited chemical controls is paramount for long-term nematode management. A new nematicide, fluazaindolizine, appears to have limited effects on beneficial nematodes and has the greatest toxicity in *Meloidogyne* spp. In a previous transcriptomic study, a cytochrome p450 *M. incognita* gene was differentially expressed across treatments with oxamyl, fluazaindolizien, fluensulfone, and fluopyram. The ortholog of this cytochrome p450 in *M. hapla*, MhA1_Contig149.frz3.fgene2, is the target for this current study, where the main goal is to develop a qPCR assay that can monitor nematicide efficacy overtime by relating detoxification gene expression in *M. hapla* after fluazaindolizne exposure to nematode inactivity. The aforementioned transcriptomic study also provided potential targets for the mode-of-action for fluazaindolizine, including a *M. incognita* putative V-type ATPase (*M. hapla* ortholog MhA1_Contig147.frz3. gene67). In *in vitro* and soil assays, expression of both gene targets in *M. hapla* second-stage juveniles (J2) was monitored over time after exposure to fluazaindolizine. *Meloidogyne hapla* J2 were exposed to 0, 20.75, 95.45 ppm of fluazaindolizine for 24 hr, 3 days, or 7 days at room temperature in microcentrifuge tubes or glass vials containing soil. At each time point, 50–60 *M. hapla* J2 from the *in vitro* assay we examined to determine the number of inactive and active. After the same time points, a modified decant/sieve method was used to extract nematodes from the soil. In both assays after each time point *M. hapla* J2 were frozen in liquid nitrogen and RNA isolated. Treatments were replicated four times and both experiments were conducted twice. To assess gene expression, cDNA was synthesized, and qPCR was performed using actin as the housekeeping gene control. The activity level of *M. hapla* J2 treated with 20.75 and 95.45 ppm of fluazaindolizine went from 75% and 60% relative to the water control after 1 day to 17.8% and 2.7% after 7 days exposure. Cytochrome p450 was upregulated across time points in *M. hapla* J2 exposed to fluazaindolizine in both the *in vitro* and soil assays. The highest relative expression was in the 95.45 ppm 1 day exposure soil treatment with >15-fold expression. The V-type ATPase was consistently downregulated in both assay types. This study demonstrates a new approach to monitoring impacts of nematicides in plant parasitic nematodes and the impacts of long-term use of nematicides in the field.

## Evaluation of sugar beet varieties and rotational crops for managing *Heterodera schachtii*

Yaghoubi, Ali, R. Yazdani and M. Quintanilla

Dept. Entomology, Michigan State University, East Lansing, MI, 48824

### Abstract

*Heterodera schachtii*, the beet cyst nematode (BCN), is one of the most detrimental plant-parasitic nematodes affecting sugar beet growth and production. BCN infections can severely reduce sugar beet yield and quality, with reported yield losses of up to 60% and decreased beet sugar content. To mitigate these impacts, the host status of different sugar beet varieties and rotational crops was evaluated for managing BCN in rotation systems. A greenhouse trial was conducted using a randomized complete block design with five replications. After consulting with Michigan Sugar Company to select the most commercially relevant varieties, eight commercial sugar beet varieties from SESVanderHave (SX2296N and SV101N), Hilleshög (2332NT, HM9865, HM2361, and HILL2238NT), and ACH Seeds (G932NT and G151) companies were evaluated, along with soybean (cultivar DF155), red clover (cultivar Canadian Mammoth), and cucumber (cultivar Expedition) as potential rotational crops. Three seeds of each sugar beet variety and rotational crop cultivar were sown in 1.5-liter pots containing a mixture of three parts sand and one part field soil. Eleven days after planting, each pot was inoculated with approximately 2000 BCN eggs. Two months post-inoculation, plant growth parameters and BCN population densities were assessed. Among the sugar beet varieties, G932NT exhibited significantly higher root weight compared to HM2361 and G151. Regarding nematode reproduction, HM9865 and G151 had significantly higher cyst numbers compared to SX2296N and G932NT, indicating their susceptibility to BCN. Similarly, HM9865 and G151 exhibited significantly higher egg production than SX2296N, G932NT, HILL2238NT, and 2332NT, further demonstrating their poor resistance against BCN multiplication. In contrast, SX2296N and G932NT exhibited relatively lower cyst and egg counts, suggesting potential resistance to BCN infestation. For rotational crops, soybean rhizosphere had 8.8 cysts and 417 eggs per 100 cm^3^ soil, while clover and cucumber were confirmed as non-hosts, with no cysts or eggs detected. This study identified promising sugar beet varieties and non-host rotational crops with potential for managing BCN populations in rotation systems. Further field evaluations are warranted to validate the efficacy of these varieties and crops for sustainable BCN management and provide growers with effective integrated pest management recommendations.

## Developing a reverse transcription-quantitave polymerase chain reaction (RT-qPCR) assay for detection of *Tobacco rattle virus* in vector stubby root nematodes

Yan, Guiping and B. Lawaju

Dept. Plant Pathology, North Dakota State University, Fargo, ND 58108, USA

### Abstract

Stubby root nematodes (SRN) are ectoparasitic nematodes known to affect host plants either by directly feeding on the roots or by vectoring plant viruses. *Tobacco rattle virus* (TRV), along with *Pea early browning virus* (PEBV), and *Pepper ringspot virus* (PRSV), is documented to be transmitted by nematodes. *Paratrichodorus allius* is the predominant SRN species found in the major potato-growing regions of the USA and is a known vector of TRV which can cause corky ringspot disease in potatoes. Detection of TRV in this vector nematode is crucial for understanding the epidemiology of the virus and designing effective strategies for managing the disease. In this study, a SYBR green-based reverse transcription-quantitative PCR (RT-qPCR) assay was developed to quantitatively detect TRV in RNA extracted from *P. allius.* A primer set targeting a region of the 16kDa protein of RNA1 of the viral genome was used in this assay. Its specificity was confirmed by testing the target TRV as well as multiple non-target potato viruses, including *Potato mop-top virus* (PMTV), *Potato leafroll virus* (PLRV), *Potato virus Y* (PVY), and *Tomato spotted wilt virus* (TSWV). Additionally, TRV was assessed in RNA samples extracted from single viruliferous nematodes in the presence of varying numbers of non-viruliferous nematodes. This assay was sensitive and could detect TRV in a single viruliferous nematode, even when present in a 1: 10 ratio of viruliferous to non-viruliferous nematodes. Furthermore, standard curves were generated using two-fold serial dilutions of RNA extracted from five *P. allius* nematodes, showing good amplification efficiency. To validate the assay, 25 soil samples were collected from a potato field previously identified with viruliferous nematodes in North Dakota. Nematodes were extracted from soil, and RNA from individual nematodes were assayed for TRV in triplicates. Eighty percent of the samples gave positive results while 20% gave negative results. Conventional PCR with a different set of primers confirmed the test results from the developed RT-qPCR. This new assay represents the first development of a RT-qPCR assay for testing TRV in *P. allius* nematodes, offering enhanced sensitivity, quantitative capability, and reduced testing time compared to conventional PCR method. It will significantly contribute to understanding the epidemiology of TRV and implementing effective disease management strategies.

## Application of artificial intelegence on nematode identification system development

Yang, Jiue-in^1^, Y. Lin^2^, H. Lai^2^, M. Hsieh^3^ and S. Chen^2^

^1^Dept. of Nematology, University of California, Riverside, Riverside, CA 92521

^2^Dept. Biomechatronics Engineering, National Taiwan University, Taipei, Taiwan

^3^Dept. Plant Pathology and Microbiology, National Taiwan University, Taipei, Taiwan

### Abstract

Effective management of plant-parasitic nematodes hinges on accurate and timely species identification. Traditional methods, predominantly reliant on morphology characteristics and morphometrical measurements, are labor-intensive, time-consuming and require skilled personnel. Consequently, the speed and scalability of analysis were limited and the locations where identifications can be performed are confined. In this study, a novel diagnostic system utilizing the Faster Region-based Transformer (Faster R-Transformer) neural network for rapid and precise identification of plant parasitic nematodes was developed. A total of 9,723 images was included in the experimental dataset used, belonging to 10 target plant-parasitic nematode species, and 3 species that characterize other feeding habits as controls. The images were annotated with 18,156 bounding boxes for head region, tail region, and whole-body. In addition, to enhance the decision output of the model, a two-stage optimization was further implemented. First, a weighted voting mechanism was utilized to ensure consistent predictions across the head, tail, and whole-body categories for the same nematode. Second, sub-models were concatenated to strengthen the discriminative ability for differentiating the easily confused species. As a result, the model achieved the mean average precision (mAP) of 0.9149 and the overall identification accuracy was improved to 0.9602. To provide further interpretation of the features used in the model, the occlusion sensitivity method was applied to identify critical recognition areas indispensable for the model. Finally, a user-friendly interface was deployed to provide a fast-screening tool to meet the practical use by plant health professionals.

## Occurrence of plant-parasitic nematodes in soybean fields of Michigan

Yazdani, Razieh, A. Yaghoubi, L. Forsberg and M. Quintanilla

Dept. Entomology, Michigan State University, East Lansing, MI, 48824

### Abstract

Soybean (*Glycine max*) is a globally significant legume crop that contributes substantially to both agriculture and the world economy. Michigan is a significant soybean-producing state in the U.S. In 2020, Michigan farmers harvested approximately 83.4 million bushels of soybeans from 2.27 million acres of land, making it the 16th largest soybean-producing state in the country, and one of the leading producers in the Midwest. Plant parasitic nematodes pose a significant challenge to soybean production systems, contributing substantially to yield reductions. Among various nematodes affecting soybean plants, the soybean cyst nematode stands out as one of the most damaging examples. It causes greater yield losses than any other soybean disease, resulting in an estimated annual reduction of $469 to $818 million. To better understand the distribution of plant parasitic nematodes in soybean production in Michigan, a survey was conducted in Fall 2023. A total of 108 soil samples were collected from 27 soybean fields. Nematodes were extracted from the soil via centrifugation method, identified at the genus level, and quantified using an inverted microscope. In this study, the most prevalent and abundant plant-parasitic nematode genera identified were *Heterodera* (incidence: 78%, abundance: 1,537 eggs and juveniles/100 cm^3^ of soil), *Helicotylenchus* (85.84%, 155 nematodes/100 cm^3^ of soil), *Merlinius* (76.41%, 33 nematodes/100 cm^3^ of soil), and *Pratylenchus* (61.32%, 17 nematodes/100 cm^3^ of soil). Less frequent and lower-density genera included *Criconemoides* (16.03%, 18 nematodes/100 cm^3^ of soil), *Xiphinema* (20.75%, 7 nematodes/100 cm^3^ of soil), *Hoplolaimus* (9.4%, 7 nematodes/100 cm^3^ of soil), and *Longidorus* (1.8%, 3 nematodes/100 cm^3^ of soil). These findings provide valuable insights into the distribution and impact of plant parasitic nematodes in Michigan soybean fields, emphasizing the need for effective management strategies to mitigate their effects on crop yield and promote sustainable soybean production.

## Pinewood nematode assay status and challenges in North Carolina Department of Agriculture & Consumer Services

Ye, Weimin

Nematode Assay Section, Agronomic Division, North Carolina Department of Agriculture & Consumer Services, Raleigh, NC 27607

### Abstract

*Bursaphelenchus xylophilus*, the pinewood nematode (PWN), is the causal agent for pine wilt disease. In North America it causes relatively minor damage to native conifers but is labeled as an EPPO-A2 pest and a quarantine nematode for many countries because of its potential for destruction of conifers. The Agronomic Division of the North Carolina Department of Agriculture & Consumer Services has a publicly operated nematode assay lab providing nematode diagnostic services and control recommendations. In the past 12 years, due to stricter regulations on PWN, a high volume of pinewood samples has been submitted to the lab from 26 states. From fiscal year 2012 to 2024, 67,225 pinewood samples were analyzed, and 7,527 reports were generated in connection with the issuance of phytosanitary certificates by USDA/APHIS/PPQ for export of pine logs. Unfortunately, trade issues with China and new USDA sampling protocols effective February 1, 2022, have had a negative impact on sample volume submitted to the lab. A large industrial funnel (30-cm diameter, 32-cm high, 6800-mL water capacity) and a movable rack (2.2-m L × 1-m W × 1.3-m H, with 18 samples fit in a rack) were designed to extract PWN. The required sample size is a minimum of 200 g of wood-drilled shavings shipped overnight to the lab. Identification is accomplished using traditional morphology, primarily the female tail shape and vulva flap and male spicule and bursa. The identification is confirmed by DNA sequencing and real-time-PCR if necessary to comply with the zero-tolerance export regulations. Overall, PWN prevalence in submitted samples is 2.1%. These results indicate the low presence of PWN in exported pine logs and the importance of regulatory measures and laboratory testing.

